# Using polygenic risk scores to evaluate definitions of self-reported sleep phenotypes across cohorts

**DOI:** 10.1093/sleep/zsag200

**Published:** 2026-07-21

**Authors:** Annah B. Wyss, Michael Brown, Xiang Li, Brian W. Spitzer, Zhijie Huang, Heming Wang, Richa Saxena, Linda C. Gallo, Qibin Qi, Wassim Tarraf, Robert Kaplan, Melissa Lamar, Hector M. González, Charles DeCarli, Myriam Fornage, Jerome I. Rotter, Stephen S. Rich, Kent D. Taylor, Xiuqing Guo, Alexis C. Wood, Peter Y. Liu, Susan R. Heckbert, Chloé Sarnowski, Jan Bressler, Alanna C. Morrison, Bing Yu, Pamela L. Lutsey, Carmen R. Isasi, Susan Redline, Tamar Sofer

**Affiliations:** 1 CardioVascular Institute, Beth Israel Deaconess Medical Center, Boston, MA, United States; 2 Department of Medicine, Harvard Medical School, Boston, MA, United States; 3 Human Genetics Center, Department of Epidemiology, School of Public Health, University of Texas Health Science Center at Houston, Houston, TX, United States; 4 Division of Endocrinology, Diabetes, and Metabolism, Department of Medicine, College of Medicine, University of Illinois Chicago, Chicago, IL, United States; 5 Division of Sleep Medicine, Brigham and Women’s Hospital, Boston, MA, United States; 6 Center for Genomic Medicine and Department of Anesthesia, Critical Care and Pain Management, Massachusetts General Hospital, Boston, MA, United States; 7 Department of Psychology, San Diego State University, San Diego, CA, United States; 8 Department of Epidemiology and Population Health, Albert Einstein College of Medicine, Bronx, NY, United States; 9 Institute of Gerontology and Department of Healthcare Sciences, Wayne State University, Detroit, MI, United States; 10 Rush Alzheimer’s Disease Center and Department of Psychiatry and Behavioral Sciences, Rush University, Chicago, IL, United States; 11 Department of Neurosciences, University of California at San Diego, La Jolla, CA, United States; 12 Department of Neurology, University of California at Davis, Davis, CA, United States; 13 Institute of Molecular Medicine, McGovern Medical School, University of Texas Health Science Center at Houston, Houston, TX, United States; 14 The Institute for Translational Genomics and Population Sciences, Department of Pediatrics, The Lundquist Institute for Biomedical Innovation at Harbor-UCLA Medical Center, Torrance, CA, United States; 15 Department of Genome Sciences, University of Virginia, Charlottesville, VA, United States; 16 USDA/ARS Children’s Nutrition Research Center, Baylor College of Medicine, Houston, TX, United States; 17 Department of Epidemiology, University of Washington, Seattle, WA, United States; 18 Division of Epidemiology & Community Health, University of Minnesota, Minneapolis, MN, United States; 19 Department of Biostatistics, Harvard T.H. Chan School of Public Health, Boston, MA, United States

**Keywords:** genetic risk score, sleep, sleep duration, insomnia, excessive daytime sleepiness

## Abstract

**Study Objectives:**

Since genome-wide association studies (GWAS) of sleep phenotypes have been conducted in differing populations and definitions of sleep phenotypes vary across studies, we investigated associations between several polygenic risk scores (PRSs) and potential sleep definitions among multiethnic cohorts.

**Materials and Methods:**

Using data from four cohorts (HCHS/SOL, ARIC, MESA, BHS, N = 16 895), we considered multiple definitions of short and long sleep, insomnia, and excessive daytime sleepiness (EDS). Polygenic risk scores (PRSs) were developed based on summary statistics from GWAS in European ancestry individuals from the UK Biobank (UKB) and from GWAS conducted in a multiethnic population from the Million Veteran Program (MVP). Study-specific analyses estimated associations between sleep PRSs and corresponding sleep measures per 1 standard deviation increase in the PRS. Models were adjusted for age, sex, ancestral principal components, and center and race as appropriate. Results were meta-analyzed across studies.

**Results:**

PRSs based on European ancestry UKB GWAS had statistically significant associations with multiple definitions of the corresponding sleep phenotypes. Associations that were most consistent across studies included: short sleep PRS with ≤6 hours (OR = 1.23, *p* = 1.80×10^−7^, p_het_ = 0.97); long sleep PRS with ≥9 hours (OR = 1.09, *p* = 6.76×10^−4^, p_het_ = 0.77); insomnia PRS with the Women’s Health Initiative Insomnia Rating Scale (WHIIRS) ≥10 or a subset of three questions ≥6 in ARIC (OR = 1.17, *p* = 5.51×10^−10^, p_het_ = 0.69); and EDS PRS with Epworth Sleepiness Scale (ESS) ≥11 (OR = 1.23, *p* = 1.83×10^−13^, p_het_ = 0.83). PRSs based on multi-ancestry MVP GWAS had weaker associations compared to those based on European ancestry only.

**Conclusions:**

By evaluating several types of sleep PRSs and sleep phenotypes, we were able to highlight which sleep PRS performed well across diverse populations and which sleep definitions better captured genetic underpinnings.

## Graphical Abstract

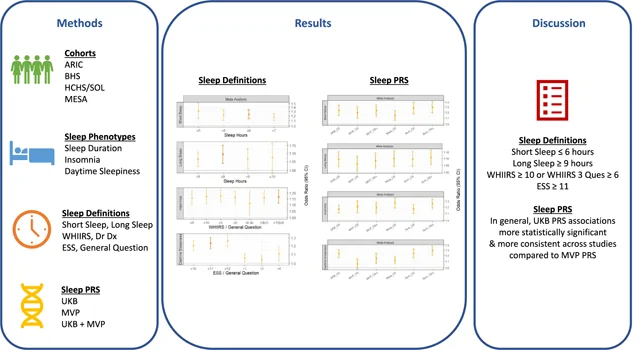

## Introduction

Approximately half of adults aged 55–85 years in the United States are estimated to have symptoms of a sleep disorder or disturbance, such as short and long sleep durations, insomnia or excessive daytime sleepiness (EDS) [1]. Sleep disorders are associated with a variety of cardiovascular- and age-related outcomes, including hypertension, myocardial infarction, stroke, diabetes, obesity, depression, and Alzheimer’s disease [1,2]. Despite the important role of sleep in the development of cardiovascular dis-eases and adverse cognitive aging, sleep disorders are not widely targeted in health interventions. This is likely in part because the biological mechanisms underlying the associations between sleep and chronic conditions are not fully understood. In addition, heterogeneity in characterizing sleep disorders across studies can hinder comprehensive assessment.

While polysomnography reliably measures many parameters of sleep, it does not capture all of its aspects [3,4]. It can also be cost and time prohibitive on a large-scale [3,4]. Often large cohort studies rely on self-reported sleep measures obtained from questionnaires to capture the multifactorial nature of sleep as well as the subjective perception of sleep [3,4]. However, sleep-related questions and their potential responses may vary between studies [3,4]. For example, sleep duration can be calculated as a continuous variable allowing for fractions of hours based on a series of questions about what time participants go to bed and wake-up on weekdays and weekends or as a discrete variable with only whole increments of hours based on a single question about how many hours of sleep participants usually get per night. Likewise, insomnia symptoms and daytime sleepiness can be ascertained based on a series of questions, such as the Women’s Health Initiative Insomnia Rating Scale (WHIIRS) [5], the Insomnia Severity Index (ISI) [6] and Epworth Sleep Scale (ESS) [7], or using single questions such as whether participants have ever been diagnosed with insomnia by a doctor or how often participants feel excessively tired during the day. Further, in the research context, it is unclear which thresholds are optimal for defining sleep disorders. For instance, meta-analyses of the associations between sleep duration and health outcomes revealed an array of study-specific thresholds to define short and long sleep [8,9]. For insomnia, Levine et al explored WHIIRS thresholds ranging from 7 to 10, suggesting a cut-point of 9 based on specificity and sensitivity but noting other cut-points may be more efficacious depending on the type of error investigators want to mitigate [10]. Studies utilizing the ESS to measure EDS have also used varying cut-points, with scores ≥10 or ≥11 frequently denoting sleepiness [11,12].

Prior genome-wide association studies (GWAS) of sleep phenotypes have relied on a variety of sleep definitions and primarily been conducted in two populations: populations of European ancestry from the UK Biobank (UKB) and a multiethnic population from the Million Veteran Program (MVP). In the UKB GWAS, self-reported sleep measures included sleep duration, short sleep (<7 hours), long sleep (≥9 hours), insomnia (single question) and daytime sleepiness (single question) [13–15]. In the MVP GWAS, short sleep (≤5 hours), long sleep (≥10 hours), and EDS (single question) were based on self-report while insomnia was based on International Classification of Diseases (ICD) codes [16,17]. Both UKB and MVP GWAS identified numerous genetic variants associated with these sleep measures. While some polygenic risk scores (PRS) developed from UKB GWAS have been associated with similar sleep traits in independent studies [18], PRS based on the MVP study remain to be developed and examined broadly.

Given the variation in sleep definitions and sleep PRSs across previous studies, we aim to build upon prior research by comprehensively assessing associations between multiple PRSs and multiple definitions of corresponding sleep phenotypes across several multiethnic cohorts. Specifically, we leveraged data from 16 895 participants in four large cohort studies (Hispanic Community Health Study/Study of Latinos [HCHS/SOL], Multi-Ethnic Study of Atherosclerosis [MESA], Atherosclerosis Risk in Communities [ARIC], and Bogalusa Heart Study [BHS]) with varying questions on sleep to estimate associations between PRS developed based on UKB and MVP GWAS and several definitions of short sleep, long sleep, insomnia and daytime sleepiness. The aims of our study were to compare PRS based on European ancestry population to PRS based on multiethnic populations to evaluate which has a stronger association with sleep measures and to distinguish the phenotype definitions with the strongest genetic associations (i.e. biologic basis) based on factors such as statistical significance and consistency across studies. We also assessed associations of sleep definitions selected based on strongest genetic associations with two additional traits available in the four studies to explore the utility of our study to inform future meta-analyses involving sleep and other health outcomes: a cognitive aging measure (Digit Symbol Substitution Test, DSST) and a cardiovascular disease-related trait (hypertension). Our approach may be especially informative for subsequent collaborative studies that rely on sleep-related genetic or non-genetic data.

## Materials and methods

### Cohort studies

#### The Hispanic Community Health Study/Study of Latinos.

The HCHS/SOL study is a longitudinal study comprising 16 415 individuals aged 18–74 years of Hispanic/Latino origin (Cuban, Puerto Rican, Dominican, Mexican, and Central/South American) [19,20]. A two-stage probability sampling design based on census-blocks groups and household addresses was employed, enrolling individuals from four geographic sites in the United States (Miami, San Diego, Chicago, and the Bronx area of New York). Individuals aged 45 to 74 years were oversampled. To account for the sampling design, sampling weights were constructed for analyses. Participants completed comprehensive clinical examinations and health questionnaires at baseline (2008–2011) and during follow-up visits (2014–2017, 2020–2024). Information on sleep [21] and genetic data were available on 12 182 individuals at baseline.

#### Multi-Ethnic Study of Atherosclerosis.

The MESA study is a longitudinal study comprising 6814 individuals aged 45–84 representing diverse self-described racial and ethnic backgrounds (approximately 38% of participants are White, 28% African American, 22% Hispanic, and 12% Asian) [22,23]. Participants had no known cardiovascular disease at time of enrollment and were recruited from six sites in the United States (Forsyth County NC, New York City NY, Baltimore City and County MD, St. Paul MN, Chicago IL, Los Angeles County CA). Baseline clinical examinations and health questionnaires were completed between 2000 and 2002, with multiple follow-up visits occurring between 2002 and 2024. We used data from MESA visit 5 for main analyses and data from MESA visit 4 in a sensitivity analysis since sleep questions were more thorough and similar to other studies in MESA visit 5 but MESA visit 4 had a larger sample size (5384 individuals at visit 4 and 2085 individuals at visit 5 with sleep and genetic data).

#### Atherosclerosis Risk in Communities.

The ARIC study is a population-based prospective cohort study of cardiovascular disease among 15 792 individuals, predominantly European Americans and African Americans, and has been described in detail previously [24]. Participants were aged 45–64 years at baseline (1987–1989) and chosen by probability sampling from four US communities (northwestern suburbs of Minneapolis, Minnesota; Washington County, Maryland; Forsyth County, North Carolina; and Jackson, Mississippi). Several follow-up visits were conducted between baseline and the present. In 1996–1998 (visit 4), 1920 ARIC participants from the Minnesota and Maryland field centers underwent sleep assessments as part of the Sleep Heart Health Study (SHHS) [25]. The SHHS Sleep Habits Questionnaire was used to elicit information about habitual sleep duration, insomnia, and EDS. Data collected during ARIC visit 4 and the SHHS was used for this analysis (1550 individuals of European ancestry with sleep and genetic data).

#### Bogalusa Heart Study.

The BHS is a community-based prospective cohort study of the natural history and risk factors of cardiovascular disease among individuals in Bogalusa, Louisiana and has been described in detail previously [26]. Approximately two-thirds of individuals are White (65%) and one-third are Black (35%) and were aged 4–38 years old at baseline in 1972. Several follow-up visits were conducted every 2–3 years between baseline and the present. In 2013–2016 (BHS Biceps), comprehensive sleep questionnaires were administered to 1298 individuals. Among individuals with sleep data, 1078 also had genetic data.

#### Ethics statement.

The HCHS/SOL was approved by the institutional review boards (IRBs) at each field center, where all participants gave written informed consent, and by the Non-Biomedical IRB at the University of North Carolina at Chapel Hill to the HCHS/SOL Data Coordinating Center. All IRBs approving the HCHS/SOL study are: Non-Biomedical IRB at the University of North Carolina at Chapel Hill, Chapel Hill, NC; Einstein IRB at the Albert Einstein College of Medicine of Yeshiva University, Bronx, NY; IRB at Office for the Protection of Research Subjects (OPRS), University of Illinois at Chicago, Chicago, IL; Human Subject Research Office, University of Miami, Miami, FL; and Institutional Review Board of San Diego State University, San Diego, CA.

For MESA, the UCLA IRB approved the field center activities. All MESA participants provided written informed consent, and the study was approved by the IRBs at The Lundquist Institute (formerly Los Angeles BioMedical Research Institute) at Harbor-UCLA Medical Center, University of Washington, Wake Forest School of Medicine, Northwestern University, University of Minnesota, Columbia University, and Johns Hopkins University.

For ARIC, written informed consent was provided by all study participants and the study design and methods were approved by IRBs at each of the collaborating medical institutions: University of Mississippi Medical Center Institutional Review Board (Jackson Field Center); Wake Forest University Health Sciences Institutional Review Board (Forsyth County Field Center); University of Minnesota Institutional Review Board (Minnesota Field Center); and Johns Hopkins Bloomberg School of Public Health Institutional Review Board (Washington County Field Center).

For BHS, all participants gave written, informed consent prior to data collection and study procedures were approved by the Tulane University Health Sciences Institutional Review Board.

All methods and analyses were carried out in accordance with human subject research guidelines and regulations. This work was approved by the Beth Israel Deaconess Medical Center Committee on Clinical Investigations.

### Sleep phenotypes

#### Sleep duration, short sleep, and long sleep.

Each study asked participants about what time they usually go to bed/fall asleep and what time they usually wake up on weekdays and weekends (Table 1). From these questions, the number of hours of sleep per night on a weekday and on a weekend were computed and an overall measure of sleep duration was calculated as (weekday_hours_sleep_per_night ×5 + weekend_hours_sleep_per_night ×2) / 7. Short sleep was defined as a binary outcome based on sleep duration using the following thresholds: ≤5, <6, ≤6, and <7 hours (Figure 1). Likewise long sleep was defined using the following thresholds: >8, ≥9, >9, and ≥10 hours.

#### Insomnia.

To ascertain insomnia, individuals in each study were asked questions from the WHIIRS (Table 1) [5]. Specifically, HCHS/SOL, MESA and BHS participants were asked to rate their overall typical night’s sleep (from “0. Very sound or restful” to “4. Very restless”) and how often they experienced the following situations (from “0. No, not in the past four weeks” to “4. Yes, 5 or more times a week”): trouble falling asleep, waking up earlier than planned, trouble getting back to sleep, and waking up several times at night. Reponses to these five questions were then summed to create an overall WHIIRS score between 0 and 20. In ARIC, three of the five WHIIRS questions were asked regarding how often individuals experienced (from “0. Never” to “4. Almost always (16–30/month)”): trouble falling asleep, waking up earlier than planned, trouble get-ting back to sleep. Responses to these three questions were then summed to create a modified score between 0 and 12. For HCHS/SOL, MESA, and BHS, we considered the following binary insomnia outcomes: WHIIRS (all 5 questions) ≥10 and ≥9 and WHIIRS (same 3 questions as ARIC) ≥6 and ≥5 (Figure 1). For ARIC, binary insomnia outcomes included WHIIRS (3 questions) ≥6 and ≥5. Par-ticipants in HCHS/SOL, MESA and BHS were also asked a general question about whether they had “ever been told by a doctor that you have insomnia?” so for these three studies we also considered a binary outcome for doctor diagnosed insomnia (yes vs no).

#### Daytime sleepiness.

To measure excessive sleepiness, all four studies used the Epworth Sleepiness Scale (ESS) [7] which asks participants how likely they are to fall asleep during the following activities (from “0. No chance” to “3. High chance”): reading, watching TV, in public, as a passenger in a car, laying down, talking, eating lunch, stopped in a car (Table 1). Responses to these eight questions were then summed to create an overall ESS score between 0 and 24 and binary versions of the outcome included ≥10, ≥11, and ≥12 (Figure 1). Participants were also asked a general question about how often in the last month “did you feel overly sleepy during the day?” with the following possible responses: “1. No”, “2. Yes, less than once a week”, “3. Yes, 1 or 2 times a week”, “4. Yes, 3 or 4 a week”, and “5. Yes, 5 or more times a week”. We then considered the following binary outcomes based on these responses: >1, >2, and >4.

### Sleep polygenic risk scores

#### Genetic data.

In HCHS/SOL, genotyping was performed using the Illumina HumanOmni2.5–8 BeadChip. Variants with missingness >5% or minor allele frequency (MAF) < 0.005, and individuals with >5% missingness across all SNPs, were removed from analysis. Imputation was then performed using the TOPMed2.0 reference panel and variants with R^2^ ≤ 0.4 or MAF < 0.01 after imputation were discarded. In MESA, whole genome sequencing data was available via TOPMed Freeze 10; details on sequencing methods and quality control are available here: https://topmed.nhlbi.nih.gov/data-resources/methods. In ARIC, genotyping was completed using Affymetrix GeneChip SNP Array 6.0. Quality control (QC) filtering was applied prior to imputation. Sample QC filtered out sex mismatch, first-degree relatives of an included individual, genetic outliers based on allele sharing and principal component (PC) analyses, and participants with low call-rate (<95%). Variant QC filtered out monomorphic SNPs, low call rate < 95%, minor allele frequency (MAF) < 0.01, and deviation from Hardy–Weinberg equilibrium (HWE, *p* < 1×10^−5^). Imputation of the genotype data using the TOPMed R2 reference panel was performed using the TOPMed Imputation server. In BHS, genotyping was completed using Illumina Human610 BeadChip [27–30]. Brief ly, sample level QC excludes individuals with sex discrepancies or a call rate below 95%. At variant level, SNPs were removed if they had a call rate < 95%, MAF < 0.01, or significant deviation from HWE (*p* < 1×10^−6^). Genotypes were imputed by the TOPMed Imputation Server [31–33]. Ancestral principal components were calculated in each study.

#### UKB PRS.

We developed PRSs for the following five sleep phenotypes: sleep duration, short sleep, long sleep, insomnia and daytime sleepiness (Figure 1). For sleep duration, short sleep and long sleep we used results from a GWAS of nearly 450 000 individuals of European ancestry from UKB which ascertained sleep duration using the single question “About how many hours of sleep do you get in every 24h?” and defined short sleep as <7 hours and long sleep as ≥9 hours [13] (Supplemental Table 1). Similarly, we used results from other large GWAS within UKB for insomnia [14] and daytime sleepiness [15], which defined insomnia based on the single question “Do you have trouble falling asleep at night or do you wake up in the middle of the night?” and EDS based on the single question “How likely are you to doze off or fall asleep during the daytime when you don’t mean to?” (Supplemental Table 1). Based on the GWAS estimates from the respective study for each sleep phenotype, we inferred posterior effect sizes of variants using PRS-CS [34], with the following settings: 1000 Genomes European panel as the reference panel, HapMap variants lifted over to hg38 as the target data, and all other parameters at the default settings. The PRS weights are then the computed posterior effect estimates. Throughout, PRSs for each sleep phenotype among participants in each study were constructed using PLINK—score [35,36] or PRSice [37] without clumping or thresholding. For analysis, PRS were scaled in each study so estimates for associations were per 1 SD increase in the PRS.

#### MVP PRS.

To compare with the PRS based on UKB data, we also constructed PRS based on recently published GWAS of sleep phenotypes using data from the MVP [16,17] (Figure 1). For short and long sleep, an MVP GWAS ascertained sleep duration using the single question “How many hours do you usually sleep each day (24-h period)?” and defined short sleep as ≤5 hours and long sleep as ≥10 hours [16] (Supplemental Table 1). This study stratified White and Black populations defined by patterns of genetic ancestry, referred to as EUR and AFR. In another GWAS of over 2000 traits in MVP, ICD codes were used to define insomnia and a single question about “feeling excessively sleepy during the day (does not include regular naps)” was used to define EDS [17] (Supplemental Table 1). GWAS summary statistics were available from four groups: EUR, AFR, AMR (primarily Hispanic ancestries), and EAS (primarily Asian ancestries). Given the multiethnic nature of the MVP data, we utilized two methods to construct PRS. First, we used PRS-CS [34] applied on the summary statistics from the EUR group only (the group with the largest sample size; using the same approach as before). Second, we used PRS-CSx [38] with the following settings: 1000 Genomes European, African, Asian, or Admixed American as reference panels corresponding to the MVP group available, same target data as before, and all other parameters at the default settings. Ancestry-specific posterior effect estimates were then used as ancestry-specific PRS weights. Ancestry-specific scaled MVP PRS-CSx were also summed to create a single multiethnic PRS.

#### UKB and MVP combined PRS.

We combined PRS across the UKB and MVP GWAS using three methods. First, we meta-analyzed GWAS summary statistics from White individuals from both studies using GWAMA v.2.2.2, with parameters left at their default settings. We used PRS-CS to develop a PRS. Second, we used simple summation to combine the scaled UKB PRS and scaled MVP PRS-CS EUR PRS. Third, we used simple summation to combine the scaled UKB PRS and the scaled MVP EUR ancestry-specific PRS extracted from the PRS-CSx analysis. The latter (PRS-CSx European ancestry) may perform better than the first (PRS-CS EUR) because it was developed while borrowing information from the other available ancestry groups. For analysis, all secondary PRSs, including PRS summations, were scaled in each study so estimates for association were per 1 SD increase in the PRS.

### Statistical analysis

Each cohort used logistic or linear regression to estimate associations between the sleep PRSs and corresponding sleep phenotypes adjusting analyses for age, sex, center, and the first five (HCHS/SOL and MESA) or 10 (ARIC and BHS) ancestral PCs. BHS models were also adjusted for race (White, Black) and HCHS/SOL models incorporated sampling weights to account for its two-stage sampling design. Analyses further adjusted for body mass index (BMI), which is often included in analyses of sleep conditions [39], and stratification by sex were also considered to qualitatively assess robustness of results (i.e. no formal criteria for change in estimates was defined). Results from study-specific analyses were meta-analyzed using a fixed effects inverse-variance weighted approach. For PRS-sleep definitions estimates with evidence of heterogeneity across studies (p_het_ < 0.05), results from random effects meta-analyses are also reported.

To evaluate various sleep PRS and definitions, we first consid-ered which associations were statistically significant at *p*-values <0.05 in meta-analyses, particularly definitions with the lowest *p*-value of each sleep phenotype. Among definitions significantly associated with sleep PRS, we next considered which definitions were most consistent across studies (i.e. highest *p*-value for heterogeneity). Finally, we considered sample size to ensure that definitions would not result in too few cases for reliable analyses.

We estimated the variance explained by each UKB PRS (on top of the other covariates in the model) and its respective consensus sleep phenotype definition using the r2_var function in the r2redux R package [40]. As it is recommended to use at least 2000 individuals in such analysis, it was performed using unrelated individuals from HCHS/SOL only.

To further substantiate the sleep definitions highlighted from the main association analyses of sleep PRSs with sleep phenotypes based on significance, consistency and sample size, we conducted secondary analyses of sleep phenotypes with the other complex and age-related traits, namely the DSST and hypertension. The DSST is a cognitive test in which participants replace a pattern of numbers with symbols based on a key [41]. Scores represent the number of correct digit-symbol pairs completed in 90 seconds (HCHS/SOL and ARIC) or 120 seconds (MESA and BHS), and scores for each study were then standardized using means and standard deviations (SDs). Hypertension was defined as systolic blood pressure (SBP) ≥140, diastolic blood pressure (DBP) ≥90 or use of hypertension medication. Study-specific analyses were conducted using “glm” (for MESA, ARIC, and BHS) and “surveyglm” (for HCHS/SOL) and meta-analyses were performed using “meta” in R [42].

## Results

### Study characteristics

Our analysis of sleep PRS and sleep phenotypes included 16 895 individuals from HCHS/SOL visit 1 (N = 12 182), MESA visit 5 (N = 2085) ARIC visit 4 (N = 1550), and BHS BiCEPS visit (N = 1078; Table 2). In each study slightly more women participated than men (50%–60% women vs 40%–50% men). Individuals from HCHS/SOL and BHS tended to be younger at the time of sleep measurements (average age 46.1 years and 48.2 years, respectively) than individuals from MESA and ARIC (average age 68.7 years and 62.7 years, respectively).

### UKB PRS

#### Short and long sleep.

The short sleep UKB PRS was significantly associated with the short sleep phenotype across studies at several thresholds. In meta-analyses, the short sleep PRS was significantly associated with all four definitions (≤5, <6, ≤6 and <7 hours) with *p*-values below 5×10^−4^. While the association with sleep duration <7 hours was most significant, it was less consistent across studies than other thresholds (OR = 1.17, 95% CI = 1.11 to 1.24, *p* = 1.13×10^−7^, p for heterogeneity = 0.57, N cases = 3701) (Figure 2, Supplemental Table 2). Estimates for the other three definitions were very similar, though the association for sleep duration ≤6 hours appeared more consistent across studies (OR = 1.23, 95% CI = 1.14 to 1.33, *p* = 1.80×10^−7^, p for heterogeneity = 0.97, N cases = 1720). For long sleep, associations tended to be slightly weaker, with the long sleep PRS significantly associated with three definitions (>8, ≥9, and >9 hours) at *p* < .05 in meta-analyses (Figure 2, Supplemental Table 2). Of these, the association with sleep duration ≥9 hours was most significant and consistent across studies (OR = 1.09, 95% CI = 1.04 to 1.15, *p* = 6.76e10^−4^, p for heterogeneity = 0.77, N cases = 3404). Finally, the sleep duration PRS was significantly and consistently associated with continuous sleep duration (*β* = 0.11, SE = 0.01, *p* = 2.60×10^−15^, p for heterogeneity = 0.69, Supplemental Figure 1).

#### Insomnia.

For the association between insomnia UKB PRS and WHIIRS, effect estimates were similar whether the measure was based on all five questions or only a subset of three questions and were significant across cohorts (Figure 2, Supplemental Table 2). For studies that asked all five WHIIRS questions (HCHS/SOL, MESA and BHS), the association between insomnia PRS and WHIIRS ≥10 (OR = 1.17, 95% CI = 1.11 to 1.24, *p* = 2.13×10^−8^, p for heterogeneity = 0.49, N cases = 4851) was slightly stronger and more consistent across studies than the association with WHIIRS ≥9 in meta-analyses (OR = 1.16, 95% CI = 1.10 to 1.22, *p* = 5.72×10^−8^, p for heterogeneity = 0.37, N cases = 5574). Based on a summary variable created from the three WHIIRS questions available in all studies, the association between insomnia PRS and WHIIRS ≥5 (OR = 1.18, 95% CI = 1.12 to 1.23, *p* = 1.89×10^−11^, p for heterogeneity = 0.55, N cases = 5759) was slightly more significant in meta-analyses but the association with WHIIRS ≥6 was slightly more consistent across studies (OR = 1.16, 95% CI = 1.10 to 1.22, *p* = 2.60×10^−9^, p for heterogeneity = 0.73, N cases = 5374). In meta-analyses combining estimates based on 5 WHIIRS questions ≥10 in HCHS/SOL, MESA and BHS and 3 WHIIRS ≥6 questions in ARIC, results were slightly stronger reflecting the increased sample size of including all studies in a single analysis (OR = 1.17, 95% CI = 1.11 to 1.22, *p* = 5.51×10^−10^ , p for heterogeneity = 0.69, N cases = 5650). Associations between insomnia PRS and self-reported doctor diagnosis of insomnia based on HCHS/SOL, MESA, and BHS were also significant (OR = 1.11, 95% CI = 1.02 to 1.21, *p* = .02, p for heterogeneity = 0.27, N cases = 1392) but not as strong as associations with WHIIRS. Finally, in analyses of the continuous WHIIRS variables, the insomnia PRS was significantly associated with both WHIIRS based on 5 questions (*β* = 0.40, SE = 0.06, *p* = 3.7×10^−11^ , p for heterogeneity = 0.32) as well as WHIIRS based on 3 questions (*β* = 0.22, SE = 0.03, *p* = 1.75×10^−10^, p for heterogeneity = 0.20, Supplemental Figure 1).

#### Excessive daytime sleepiness.

For EDS we considered associations between the UKB PRS and two outcomes: ESS score and a single question about how often the study participant felt overly sleepy during the past month. For each outcome we considered three thresholds. In meta-analyses, the daytime sleepiness PRS was significantly associated with ESS at all three definitions (≥10, ≥11 and ≥12) with *p*-values at or below 5×10^−13^ (Figure 2, Supplemental Table 2). While the association for ESS ≥12 was the most significant (OR = 1.26, 95% CI = 1.19 to 1.34, *p* = 6.62×10^−15^, p for heterogeneity = 0.65, N cases = 2238), it was less consistent across studies than other thresholds. Among the other thresholds, results were similar for ESS ≥10 and ESS ≥11, but slightly more significant and consistent for ESS ≥11 (OR = 1.23, 95% CI = 1.16 to 1.30, *p* = 1.83×10^−13^, p for heterogeneity = 0.83, N cases = 2792). Associations between the daytime sleepiness PRS and the single question were weaker and generally not significant, though the association contrasting the highest frequency of sleepy days vs all other lower categories was significant (OR = 1.10, 95% CI = 1.03 to 1.19, *p* = .008, p for heterogeneity = 0.20). Finally, in analyses of the continuous ESS variable, the EDS PRS was significantly and consistently associated with ESS (*β* = 0.47, SE = 0.05, *p* = 1.24×10^−24^ , p for heterogeneity = 0.97, Supplemental Figure 1).

### UKB PRS (additional analysis)

In addition to the main meta-analyses already presented which relied on sleep data from MESA visit 5 (Figure 2, Supplemental Table 2), we also ran meta-analyses using sleep data from MESA visit 4 (Supplemental Figure 2, Supplemental Table 3), which had less detailed information on sleep phenotypes but a much larger sample size (5384 individuals with sleep and genetic data at visit 4 compared to 2085 individuals at visit 5). Sleep duration in MESA visit 4 was ascertained based on a single question “how much sleep do you usually get at night?” with discrete whole hour responses and insomnia was based on only three WHIIRS questions. Yet supplemental meta-analyses utilizing MESA visit 4 mostly highlighted the same sleep definitions as the main meta-analyses (Table 3). For short and long sleep UKB PRS, associations with ≤6 hours (OR = 1.26, 95% CI = 1.18 to 1.34, *p* = 1.98×10^−13^ , p for heterogeneity = 0.85) and ≥9 hours (OR = 1.12, 95% CI = 1.07 to 1.18, *p* = 1.63×10^−5^, p for heterogeneity = 0.76) were the most significant and most consistent across studies. For the insomnia UKB PRS, the association with WHIIRS 5 questions ≥10 or 3 questions ≥6 remained highly significant (OR = 1.18, 95% CI = 1.13 to 1.23, *p* = 8.64×10^−14^ , p for heterogeneity = 0.51). For the daytime sleepiness UKB PRS, the associations with ESS ≥12 was most significant (OR = 1.27,95% CI = 1.21 to 1.34, *p* = 7.69×10^−20^, p for heterogeneity = 0.69) while the association with ESS ≥11 was similar and most consistent across studies (OR = 1.23, 95% CI = 1.18 to 1.29, *p* = 5.45×10^−18^ , p for heterogeneity = 0.83).

In analyses stratified by sex, estimates for short sleep and excessive daytime sleepiness (ESS) were slightly higher in females and estimates for long sleep and insomnia (WHIIRS) were slightly higher in males, but differences did not appear to be significant in meta-analyses (Supplemental Figure 3). Estimates were similar when further adjusted for BMI (Supplemental Table 4).

We also estimated percent variance explained by each UKB PRS in HCHS/SOL with respect to the following definitions of sleep measures: short sleep ≤6 hours (r^2^ = 0.16%, 95% CI = 0.04 to 0.33, *p* = 1.11×10^−5^); long sleep ≥9 hours (r^2^ = 0.21%, 95% CI = 0.07 to 0.39, *p* = 8.21×10^−7^); WHIIRS ≥10 (r^2^ = 0.27%, 95% CI = 0.11 to 0.48, *p* = 1.43×10^−8^); and ESS ≥11 (r^2^ = 0.47%, 95% CI = 0.25 to 0.73, *p* = 3.85×10^−14^). For continuous measures of sleep, the percent variance explained by each PRS was: sleep duration (r^2^ = 0.40%, 95% CI = 0.20 to 0.65, *p* = 5.70×10^−12^); WHIIRS (r^2^ = 0.45%, 95% CI = 0.24 to 0.72, *p* = 1.23×10^−13^); and ESS (r^2^ = 0.76%, 95% CI = 0.47 to 1.09, *p* = 4.25×10^−22^).

### MVP PRS

To evaluate PRSs based on the MVP GWAS, we first constructed EUR PRS using PRS-CS and EUR, AFR, AMR, and EAS PRS using PRS-CSx, including a multiethnic PRS which summed the ancestry-specific PRS-CSx, in HCHS/SOL and MESA. The multiethnic PRSs did not perform better than the MVP EUR PRS-CSx alone (nor the UKB PRS) based on statistical significance in meta-analyses and consistency of estimates across studies, with some of the multiethnic MVP PRSs not associated with their respective sleep pheno-types depending on study and definition (Supplemental Table 5). Therefore, we focused on the MVP EUR PRS-CS and MVP EUR PRS-CSx in subsequent meta-analyses among all studies (HCHS/SOL, MESA, ARIC and BHS). The MVP EUR PRSs were associated with many definitions of the respective sleep phenotypes, with the most significant associations for short and long sleep again being sleep duration ≤6 hours (PRS-CSx) and <7 hours (PRS-CS) and ≥9 hours (PRS-CS and PRS-CSx). However, the MVP EUR PRSs did not appear to perform better than the UKB PRSs with respect to statistical significance in meta-analyses or consistency of estimates across studies (Figure 3, Supplemental Tables 6, 7, and 8). For example, although both the MVP EUR PRS-CS (OR = 1.19, 95% CI = 1.09 to 1.30, *p* = 8.37×10^−5^, p for heterogeneity = 0.04) and MVP EUR PRS-CSx (OR = 1.23, 95% CI = 1.13 to 1.34, *p* = 2.36×10^−6^, p for heterogeneity = 0.04) for short sleep were associated with sleep duration ≤6 hours, these associations were weaker and less consistent than the UKB PRS association (also based on European ancestries and PRS-CS software). Likewise, the MVP EUR PRS-CS (OR = 1.06, 95% CI = 1.01 to 1.12, *p* = .03, p for heterogeneity = 0.56) and MVP EUR PRS-CSx (OR = 1.08, 95% CI = 1.03 to 1.14, *p* = .002, p for heterogeneity = 0.52) for long sleep were associated with sleep duration ≥9 hours but these associations were weaker and less consistent than the UKB PRS association. For insomnia, the MVP EUR PRSs was associated with WHIIRS, whether based on 5 questions, 3 questions or a combination of thresholds (e.g. OR = 1.19, 95% CI = 1.10 to 1.27, *p* = 3.11×10^−6^, p for heterogeneity = 0.49 for MVP EUR PRS CSx and WHIIRS≥10 or WHIIRS 3 questions≥6), but the UKB PRS association again appeared stronger. Interestingly, the MVP PRSs were more strongly associated with doctor diagnosed insomnia than the UKB PRS across all studies and in the meta-analysis (e.g. OR = 1.45, 95% CI = 1.25 to 1.68, *p* = 7.39×10^−7^, p for heterogeneity = 0.16 for MVP PRS CSx), likely reflecting that the MVP GWAS used ICD codes to define insomnia while the UKB GWAS used a single questionnaire item to define insomnia. For EDS, MVP EUR PRS-CS was not associated with any of the thresholds of ESS nor the general questions. In contrast, MVP EUR PRS-CSx was significantly associated with all three thresholds of ESS and some thresholds of the general question. The strongest association was for ESS ≤10, though associations with ESS ≤11 were similar and more consistent across studies. While the MVP EUR PRS-CSx was associated with ESS ≥11 (OR = 1.15, 95% CI = 1.07 to 1.24, *p* = 2.32×10^−4^, p for heterogeneity = 0.29), the association with UKB PRS was again stronger.

### UKB and MVP combined PRS

Among the UKB and MVP combined PRSs, the PRS which summed the UKB and MVP EUR PRS-CSx performed better with respect to statistical significance in meta-analyses and consistency across studies than the other two UKB and MVP combined PRSs which utilized other methods (Figure 3, Supplemental Tables 6, 7, and 8). Further, the PRS which summed UKB and MVP EUR PRS-CSx appeared to perform slightly better than the primary UKB only PRS with respect to statistical significance, though not always with respect to consistency across studies. For example, the summed UKB and MVP EUR PRS-CSx PRS for each respective sleep trait was associated with ≤6 hours (OR = 1.30, 95% CI = 1.19 to 1.41, *p* = 2.14×10^−9^, p for heterogeneity = 0.35), WHIIRS≥10 or WHIIRS 3 questions≥6 (OR = 1.26, 95% CI = 1.18 to 1.34, *p* = 5.08×10^−13^ , p for heterogeneity = 0.43), and ESS ≥11 (OR = 1.30, 95% CI = 1.21 to 1.38, *p* = 3.83×10^−14^ , p for heterogeneity = 0.24), with better statistical significance but poorer consistency across studies than the associations for UKB only PRSs. For long sleep, the summed UKB and MVP EUR PRS-CSx PRS was associated with ≥9 hours (OR = 1.11, 95% CI = 1.06 to 1.17, *p* = 4.91×10^−5^, p for heterogeneity = 0.95), with both better statistical significance in meta-analyses and consistency across studies than the UKB only PRS.

### DSST and hypertension

To evaluate whether the sleep phenotype definitions selected based on sleep PRS are also associated with cognitive aging and cardiometabolic outcomes, we also characterized associations of sleep phenotypes with DSST and hypertension.

Analyses of sleep phenotypes and standardized DSST scores appeared to highlight the same sleep definitions identified based on sleep PRS (Table 3). For short sleep, the association between ≤6 hours and DSST was strongest in fixed effects meta-analyses (*β* = −0.07, SE = 0.03, *p* = .03, p for heterogeneity = 0.04) and random effects meta-analyses (*β* = −0.09, SE = 0.05, *p* = .10) which we also ran given heterogeneity of estimates across studies (Figure 4, Supplemental Table 9). For long sleep, the association between ≥9 hours and DSST was most significant and consistent across studies (*β* = −0.23, SE = 0.03, *p* = 1.46×10^−19^, p for heterogeneity = 0.96). Associations of insomnia with DSST were significant and similar across thresholds of WHIIRS, with the lowest *p*-values for WHIIRS ≥10 (or WHIIRS ≥6 for ARIC) (*β* = −0.07, SE = 0.02, *p* = 9.11×10^−4^, p for heterogeneity = 0.01) in the fixed effects meta-analyses and WHIIRS ≥9 (or WHIIRS ≥5 for ARIC) (*β* = −0.09, SE = 0.04, *p* = .03, p for heterogeneity = 0.02) in the random effects meta-analyses. Likewise, associations of daytime sleepiness with DSST were significant and similar across thresholds of ESS, with the lowest *p*-values for ESS ≥11 in both fixed-effects meta-analyses (*β* = −0.08, SE = 0.03, *p* = .001, p for heterogeneity = 0.08) and random-effects meta-analyses (*β* = −0.10, SE = 0.04, *p* = .02). The general questions about doctor diagnosis insomnia and feeling sleepy during the day were also significantly associated with DSST (*β* = −0.19, SE = 0.04, *p* = 2.49×10^−6^ and p for heterogeneity = 0.05 for doctor diagnosed insomnia and *β* = −0.15, SE = 0.04, *p* = 7.21×10^−5^ and p for heterogeneity = 0.39 for the highest level vs all other levels).

For analyses of sleep phenotypes and hypertension, results were more mixed (Table 3). None of the short sleep definitions were statistically associated with hypertension, with the lowest *p*-values observed with sleep duration ≤5 (OR = 1.12, 95% CI = 0.88 to 1.43, *p* = .36, p for heterogeneity = 0.89) and <7 hours (OR = 0.96, 95% CI = 0.87 to 1.06, *p* = .39, p for heterogeneity = 0.71, Figure 5, Supplemental Table 10). For long sleep and hypertension, fixed-effects meta-analyses pointed to associations for ≥9 hours of sleep (OR = 1.24, 95% CI = 1.10 to 1.39, *p* = 3.99×10^−4^, p for heterogeneity = 0.002). However, since associations across all long sleep definitions and hypertension varied noticeably by study (p for heterogeneity <0.05), we also ran random effects meta-analyses. None of the associations were significant using random effects meta-analysis, with the lowest *p*-value for duration >8 hours (OR = 1.14 95% CI = 0.94 to 1.39, *p* = .19). Likewise, significant heterogeneity was observed across studies for estimates of the association between most insomnia definitions and hypertension, with WHIIRS ≥10 (or WHIIRS ≥6 for ARIC) identified in fixed effects meta-analyses (OR = 1.16, 95% CI = 1.06 to 1.26, *p* = 1.17×10^−3^, p for heterogeneity = 0.03) and WHIIRS ≥9 (or WHIIRS ≥5 for ARIC) in random effects meta-analyses (*β* = 1.13, SE = 0.98–1.30, *p* = .10, p for heterogeneity = 0.09). For daytime sleepiness and hypertension, none of the associations were significant, with the lowest *p*-value being ESS ≥12 (OR = 1.04, 95% CI = 0.92 to 1.18, *p* = .51, p for heterogeneity = 0.26). For both insomnia and EDS, the general questions were significantly associated with hypertension (OR = 1.44, 95% CI = 1.21 to 1.71, *p* = 4.28×10^−5^ and p for heterogeneity = 0.50 for doctor diagnosed insomnia and OR = 1.15, 95% CI = 0.99 to 1.32, *p* = .06, and p for heterogeneity = 0.67 for the highest level vs all other levels).

## Discussion

In this large meta-analysis of sleep PRSs and sleep measures, sleep PRSs based on UKB GWAS were associated with several definitions of their respective phenotypic traits. Sleep PRS based on MVP GWAS were also associated with several sleep definitions, though associations were generally weaker than those for the UKB PRS. Of the sleep definitions we examined, the following definitions appeared to have the strongest evidence based on statistical significance, consistency across studies and sample sizes: short sleep ≤6 hours, long sleep ≥9 hours, WHIIRS ≥10 (or WHIIRS ≥6 if only 3 questions available), and ESS ≥11. Additional analyses of sleep measures with standardized DSST scores and hypertension seemed to support these thresholds. A summary of results across analyses considered is provided in Table 3.

The sleep duration UKB PRS was significantly and consistently associated with continuous sleep duration across the cohorts in our study. Since associations between sleep duration and several health conditions, as well as mortality, are often marked by a U-shaped pattern [8,9,43,44], with sleeping too few or too many hours associated with higher risk, previous studies have typically characterized sleep duration into three categories: short sleep, normal sleep, and long sleep. Thresholds for these categories have varied across studies, though recent studies, including the UKB GWAS upon which the primary PRS was built, tend to define short sleep as <7 hours and long sleep as ≥9 hours. In our study the short sleep UKB PRS was significantly associated with sleep <7 hours. However, associations with other thresholds were also significant and appeared more consistent across studies, particularly ≤6 hours. For long sleep, the UKB PRS was significantly and consistently associated with several thresholds, though the association with ≥9 hours appeared strongest. For insomnia, we explored associations between the UKB PRS and two often used metrics, WHIIRS and self-reported doctor-diagnosed insomnia. A WHIIRS threshold of 9 has been previously suggested based on a study demonstrating this cut-point had the largest specificity (0.67) associated with a sensitivity above 0.5 [45]. In previous HCHS/SOL studies, a threshold of ≥10 has been used [46]. In the present study we considered WHIIRS≥9 and WHIIRS≥10, finding that the association between the insomnia UKB PRS and WHIIRS≥10 was slightly stronger and more consistent than with WHIIRS≥9. Since one study (ARIC) did not have information for all the standard WHIIRS questions, we also created and evaluated an abbreviated WHIIRS variable based on 3 questions. Reassuringly, estimates for the association between the PRS and 3-question WHIIRS phenotype were almost identical to estimates for the association with the 5-question WHIIRS phenotype. Furthermore, in meta-analyses combining estimates based on the two definitions, particularly WHIIRS ≥10 and WHIIRS (3 questions) ≥6, associations with the PRS were highly significant and results were not heterogeneous across studies. This finding supports combining WHIIRS data from studies with varying levels of completeness in future multicohort collaborations. The UKB PRS was also associated with insomnia diagnosed by a doctor, but associations varied more by study and were weaker in meta-analyses than WHIIRS, consistent with the known under-diagnosis of insomnia [47]. Similar to our findings for insomnia, the daytime sleepiness UKB PRS was more strongly associated with the phenotype summarizing responses to multiple questions than the phenotype based on a single question. Specifically, we found significant associations between the PRS and ESS based on several cut-points (≥10, ≥11, and ≥12). Although the association between the PRS and ESS ≥12 was the most significant, the association with ESS ≥11 yielded similar estimates and appeared more consistent across studies. Further, ESS ≥11 resulted in more cases than ESS ≥12 yielding more precise estimates and was more strongly associated with DSST. Previous studies of ESS have also often used a threshold of ESS ≥11 based on early research by Johns et al. demonstrating this cutoff had a sensitivity of 93.5% and a specificity of 100% when identifying narcolepsy [11,48]. Furthermore, a recent study summarizing studies which investigated the test–retest reliability of ESS found that while discrete ESS values tended to vary between visits, using a binary definition of ESS ≥11 consistently showed moderate to substantial agreement with Cohen’s kappa statistic typically between 0.5 and 0.7 [12]. The daytime sleepiness UKB PRS was not associated with most thresholds of the general question “Did you feel excessively/overly sleepy during the day?”

We also considered associations for sleep PRSs based on recent multiethnic MVP GWAS with their respective sleep traits. We initially evaluated multiethnic as well as European ancestry (EUR)-specific PRSs in HCHS/SOL (Hispanic/Latino individuals) and MESA (multiethnic individuals). Surprisingly, in both studies EUR-specific PRSs performed better than multiethnic ones. This lack of improvement in performance is disappointing given the well-known drop in performance of EUR-based PRS in non-EUR populations, and especially in populations of substantial African ancestries [49].The MVP EUR PRSs were associated with many thresholds of their respective sleep phenotypes, but seemed to highlight the same sleep definitions as the UKB PRSs, in particular short sleep ≤6 hours, long sleep ≥9 hours, and ESS ≥11. However, associations were often weaker and less consistent across all studies (HCHS/SOL, MESA, ARIC and BHS) for the MVP EUR PRSs compared to the UKB PRSs. This was true whether the MVP PRS were constructed using PRS-CS based solely on European ancestry or using PRS-CSx to borrow information across multiple ancestries. The only sleep definition for which an MVP PRS seemed to perform better than the UKB PRS across studies and in meta-analyses was doctor diagnosed insomnia. This is likely because the MVP GWAS used ICD codes to define insomnia while the UKB GWAS used a single questionnaire item to define insomnia. In addition to differences in ancestry and questions used to ascertain phenotypes, MVP also differs from UKB with respect to sex as MVP is predominantly male. Although these differences may have in part influenced PRS performance in the cohorts included in our study, further evaluation in additional cohorts is warranted to further understand difference in PRS performance.

We also constructed PRSs that combined UKB and MVP using three methods, of which the PRS which summed the UKB PRS and MVP EUR PRS-CSx appeared to be most significantly associated with several definitions of sleep traits. Compared to the UKB PRS associations, the UKB and MVP EUR combined PRS associations were often stronger but less consistent across studies. Although associations between the UKB and MVP EUR combined PRS and sleep traits were often slightly more statistically significant (i.e. lower *p*-values) than associations between the UKB only PRS and sleep traits, the effect estimates were often very similar. For example, the summed UKB PRS and MVP EUR PRS-CSx was most significantly associated with short sleep≤6 hours (OR = 1.29, 95% CI = 1.19 to 1.41, *p* = 2.14×10^−9^, p for heterogeneity = 0.35), but this same association using UKB PRS was similar and more consistent across studies (OR = 1.23, 95% CI = 1.14 to 1.33, *p* = 1.80×10^−7^, p for heterogeneity = 0.97).

When we explored associations between sleep definitions and cognitive and cardiovascular conditions, none of the short sleep definitions were associated with DSST and hypertension, though associations appeared stronger for ≤6 hours than other thresholds. Although long sleep was also not associated with hypertension, all definitions of long sleep were associated with DSST, with the most significant association for ≥9 hours. Since associations between sleep duration and hypertension may follow a U-shaped pattern as just noted, we also examined short sleep-hypertension associations excluding long sleepers and long sleep-hypertension excluding short sleepers, such that the referent group in both analyses was those reporting normal sleep duration (>6 hours and <9 hours). However, estimates were similar after these exclusions (short sleep ≥6 hours fixed-effects OR = 0.99, 95% CI = 0.85 to 1.14, *p* = .86, p for heterogeneity = 0.75 and long sleep ≥9 hours random-effects OR = 1.46, 95% CI = 0.91 to 2.34, *p* = .12, p for heterogeneity = 0.002 based on meta-analyses of HCHS/SOL, MESA, and ARIC). As noted in previous literature associations between long sleep and hypertension or DSST may also be impacted by other comorbidities (i.e. individuals with other underlying health problems may sleep longer) [50]. For insomnia, several definitions were associated with both DSST and hypertension, particularly WHIIRS≥10. Although none of the definitions of daytime sleepiness were associated with hypertension, several definitions were associated with DSST, particularly ESS ≥11.

Although it is possible that the definitions highlighted in our analyses may reflect some degree of preference based on the sleep definitions in the GWASs used for PRS construction (Supplemental Table 1), associations with other thresholds suggest that the PRS captures underlying genetic predisposition for a phenotype despite shifts in definitions. For example, the UKB GWAS for insomnia was based on the case–control definition on a single question “Do you have trouble falling asleep at night or do you wake up in the middle of the night?”. Yet, we found strong evidence for an association between the PRS and WHIIRS based on 5 or 3 questions. Similarly, the UKB GWAS for daytime sleepiness asked participants “How likely are you to doze off or fall asleep during the daytime when you don’t mean to?” Yet, we did not find strong associations between the PRS and a similar question in our study. Instead, the PRS was strongly associated with the ESS.

In addition, similarity in genetic background between the GWASs used for PRS construction and the cohorts included in our study may also influence our results. The primary UKB PRSs were based on GWAS results among populations of European ancestry, while our study included cohorts representing multiple ancestries. MESA and BHS are multiethnic cohorts, while individuals in HCHS/SOL are Hispanics/Latinos and individuals in ARIC included in this analysis are White. Although developing the PRS based on European GWAS results may limit prediction in non-European populations, we noted reassuringly similar associations between the UKB PRS and sleep measures across all cohorts in our paper. Furthermore, the secondary MVP PRS which were based on multiethnic GWAS did not perform as well as the UKB PRS based on European ancestry (Supplemental Tables 5–8). However, more GWAS are needed in diverse populations to further refine PRS based on non-European ancestries.

Another limitation of our study was that we were limited to evaluating the sleep questions and scales available in the HCHS/SOL, MESA, ARIC, and BHS so alternative questions and scales (e.g. the Patient Reported Outcomes Measurement Information System PROMIS sleep scale or ISI) should be evaluated in other cohorts with such data. Although our study included four sleep phenotypes and several definitions per phenotype, we did not correct *p*-values for multiple comparisons as the tests were not independent. Indeed, many of the definitions considered for a particular sleep phenotype have some overlap and are not fully distinct (e.g. many individuals defined as long sleepers based on sleep duration >9 or ≥9 are the same). The similarity of thresholds used in sleep phenotype definitions was necessitated by differing questions across studies (e.g. sleep duration >9 and ≥9 were both considered because some cohorts ascertain sleep duration in increments of whole hours while other cohorts ascertain more precise partial hours). Moreover, we treated each phenotype as a separate analysis as has been done for other potentially correlated phenotypes (e.g. FEV1, FVC and FEV1/FVC for pulmonary function). Therefore, we utilized a nominal significance level (*p* < .05) and focused on comparing associations within each phenotype. However, it is also important to note that given the similarity of several sleep definitions within sleep phenotypes, associations were often similar across thresholds (i.e. similar estimates and overlapping 95% CIs) and had we corrected for multiple comparisons fewer associations would meet statistical significance criteria. Further, it is possible that some heterogeneity in results across definitions for a particular sleep phenotype may reflect to some degree differences in the proportion of cases meeting that criterion across studies rather than genetic vulnerability alone. For example, the percent of individuals classified as cases for short sleep ≤6 hours did not seem to vary greatly in HCHS/SOL, MESA and ARIC (7.7%–9.9%), but was higher in BHS (27.1%) and the percent of individuals classified as cases for long sleep ≥9 hours was similar in HCHS/SOL and MESA (23.4%–24.4%) but higher than ARIC and BHS (6.2–6.8%, Table 2). Additionally, it is important to note that the more restrictive definitions (e.g. short sleep ≤5 hours or long sleep ≥10 hours) not only result in a smaller number of cases but also likely reflect more extreme phenotypes. For these reasons, we compared associations between sleep PRS and sleep phenotypes based on several factors including statistical significance, consistency across studies, and sample sizes.

Finally, previous research has shown high correlation between PRS derived from a variety of genotyping methods (i.e. differing arrays + imputation vs WGS) [51]. Although differences in genotyping methods can impact PRS based on a large number of INDELS or when using PRS for risk stratification [51], our study did neither. Further, while differences in pre-phasing and imputation tools across studies can impact scores at an individual level, PRS variability is minimal (<5 percentile rank change) and therefore does not usually impact interpretability and reproducibility at the population level [52]. Therefore, we do not believe differences in genotyping methods across the cohorts used in our study would lead to any large-scale bias.

In general, harmonizing data can be challenging given the wide-array of field-specific situations and potential pitfalls including access to individual-level data, differences in data content and quality, balancing internal and external validity, and infrastructure to harmonize data [53,54]. Despite such challenges, numerous initiatives in the field of epidemiology have been established to harmonize data for a wide-variety of exposures and outcomes [54]. Here we sought to use the PRS as another tool to evaluate consensus definitions across studies. Although the low estimated percent variance explained by sleep PRS across sleep measures is expected given that PRS typically have r^2^ <1% across most phenotypes [55], it does indicate that PRS alone are insufficient to establish clinical definitions. Nevertheless, sleep PRS can complement demographic and environmental information to comprehensively evaluate sleep outcomes and help highlight which sleep definitions are associated with sleep genetics. Given the differences in sleep questions and potential responses across studies our analysis focused on binary definitions of sleep rather than continuous measures. For example, since WHIIRS was based on five questions in some studies and three questions in others, we focused on analyzing high versus low scores rather than the continuous metric. This approach helped to evaluate consensus definitions of sleep measures even when questions and potential responses differed slightly. Additionally, previous studies of sleep, including the GWAS used to develop PRS, have often utilized binary sleep definitions since investigators are often trying to characterize whether individuals have a sleep disorder or not, and the factors that predict that dichotomization. However, when the research goal is to study whether a sleep trait has a biological relationship with a gene or biomarker then continuous measures of sleep may help capture further biological variation. To this end, we did note stronger associations between sleep PRS and continuous sleep measures compared to binary sleep measures.

By utilizing data from four large cohorts, we were able to comprehensively characterize the relationship between sleep PRSs and sleep measures across diverse populations. Previous studies have used a variety of questions and thresholds to define sleep measures and a variety of populations to study sleep genetics. Therefore, our study extends the existing literature by evaluating which sleep PRS are consistently associated with which sleep questions and dichotomizations. Downstream, this analysis may help inform which sleep PRSs and sleep definitions to prioritize in future genetic and non-genetic studies, especially for cross-study collaborations. For example, meta-analyses for sleep and cognitive aging measures can benefit from harmonized sleep measures.

In summary, we found short and long sleep PRSs were associated with several thresholds of sleep duration, with slightly stronger and more consistent associations for ≤6 hours and ≥9 hours respectively. For insomnia, estimates for the association between the PRS and WHIIRS were similar whether the outcome was based on five or three questions; for example, WHIIRS 5 questions ≥10 or 3 questions ≥6. The insomnia PRS was also associated with self-reported doctor-diagnosed insomnia but associations were generally weaker than with WHIIRS. For EDS, the PRS was associated with several thresholds of ESS, including ESS ≥11 and ESS ≥12, which asked a series of questions but not a single general question about how often individuals felt overly sleepy during the day. Associations between sleep measures and DSST or hypertension seemed to support definitions and thresholds highlighted by the sleep PRS. Interestingly, PRS based on UKB GWAS among populations of European ancestry seemed to be more strongly associated with sleep measures than PRS based on MVP GWAS among multiethnic populations, especially given that our study population included 4 large diverse cohorts. Based on validation in additional studies, the sleep PRS and definitions highlighted in our paper may help inform future prospective studies and can be useful for subsequent collaborative studies on a variety of sleep-related topics.

## Supplementary Material

Supplement

Supplementary material is available at *SLEEP* online.

## Figures and Tables

**Figure 1.**
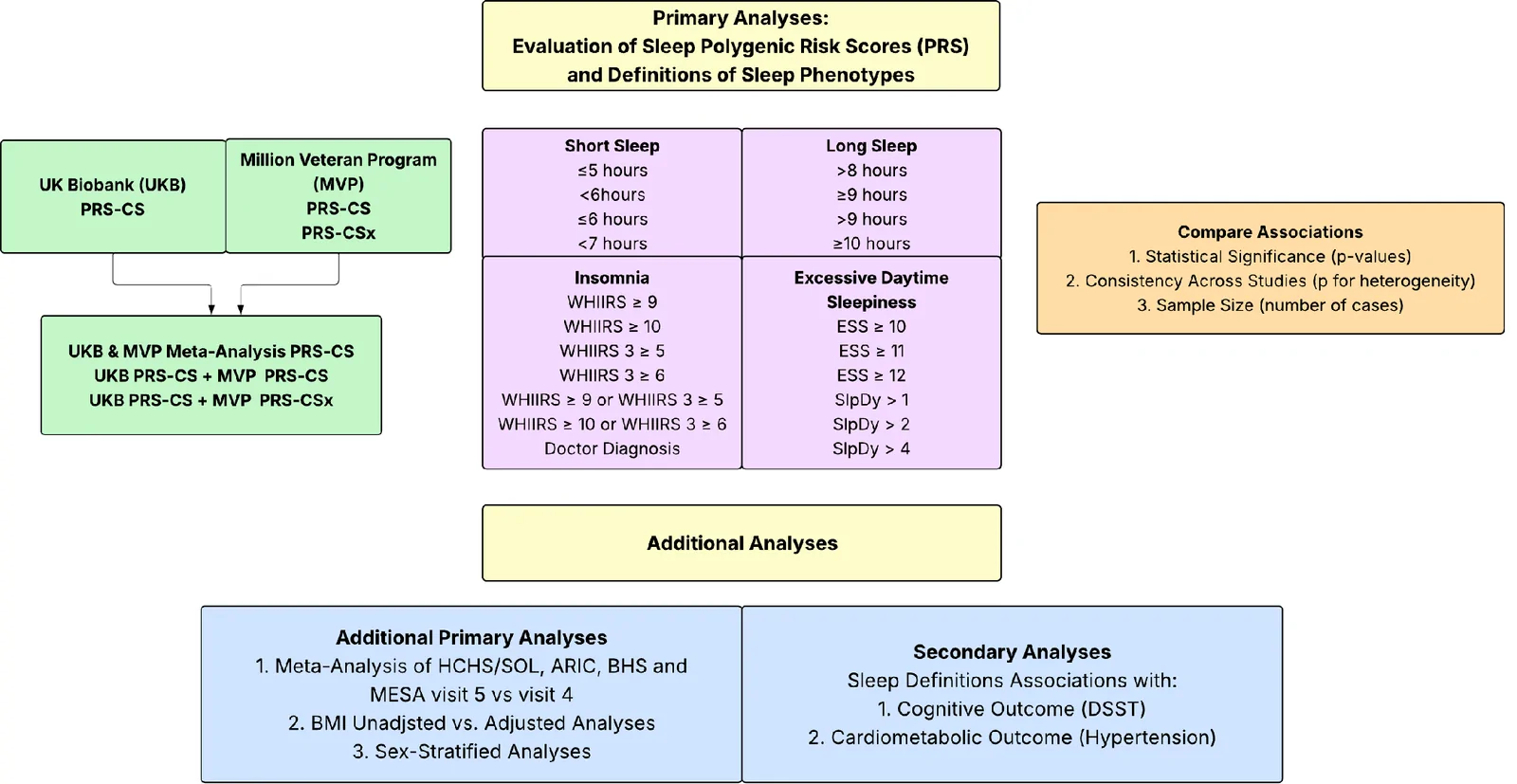
Analysis framework. Primary analyses evaluated associations of sleep polygenic risk scores (PRS) with several definitions of sleep phenotypes using data from HCHS/SOL (visit 1), ARIC (visit 4), BHS (biceps visit), and MESA (visit 5). Sleep PRS were developed based on UK Biobank (UKB) and Million Veteran Program (MVP) genome-wide association analyses (GWAS). Sleep phenotypes included short sleep, long sleep, insomnia and excessive daytime sleepiness. Associations between sleep PRS and definitions of sleep phenotypes were compared based on statistical significance in meta-analyses, consistency across studies, and sample sizes. Additional primary analyses included meta-analyses using MESA visit 4 instead of visit 5, BMI adjusted analyses, and sex-stratified analyses. Secondary analyses included associations of sleep definitions with hypertension and digit symbol substitution test (DSST).

**Figure 2.**
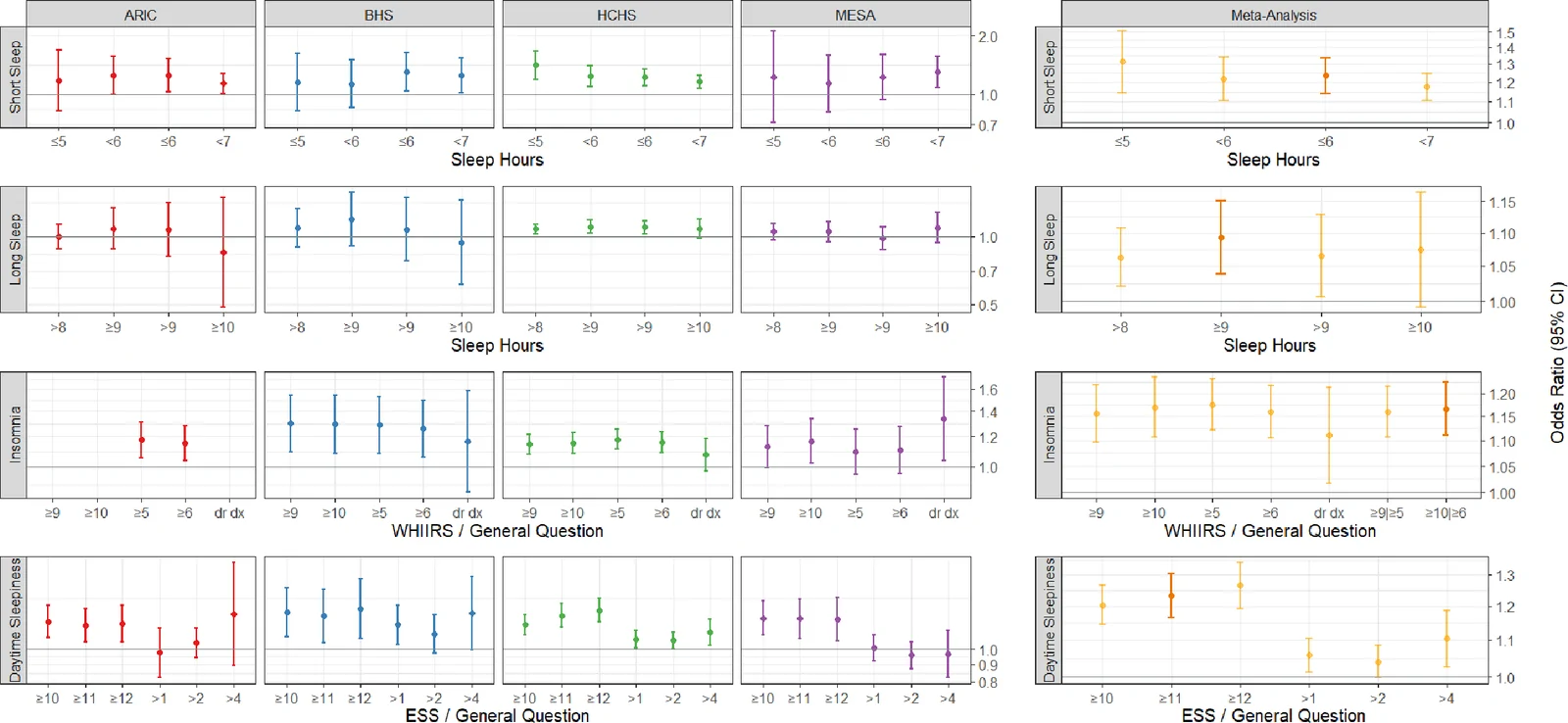
Study-specific and meta-analysis results for the association between UKB sleep PRSs and sleep phenotype definitions for short sleep, long sleep, insomnia and excessive daytime sleepiness. Study-specific results are presented for ARIC (red), BHS (blue), HCHS/SOL (green) and MESA (purple). Meta-analyses results are presented in orange, with bolded associations between UKB sleep PRS and the following sleep phenotype definitions: short sleep ≤6 hours, long sleep ≥9 hours, WHIIRS ≥10 (or WHIIRS ≥6 if only 3 questions available), and ESS ≥11.

**Figure 3.**
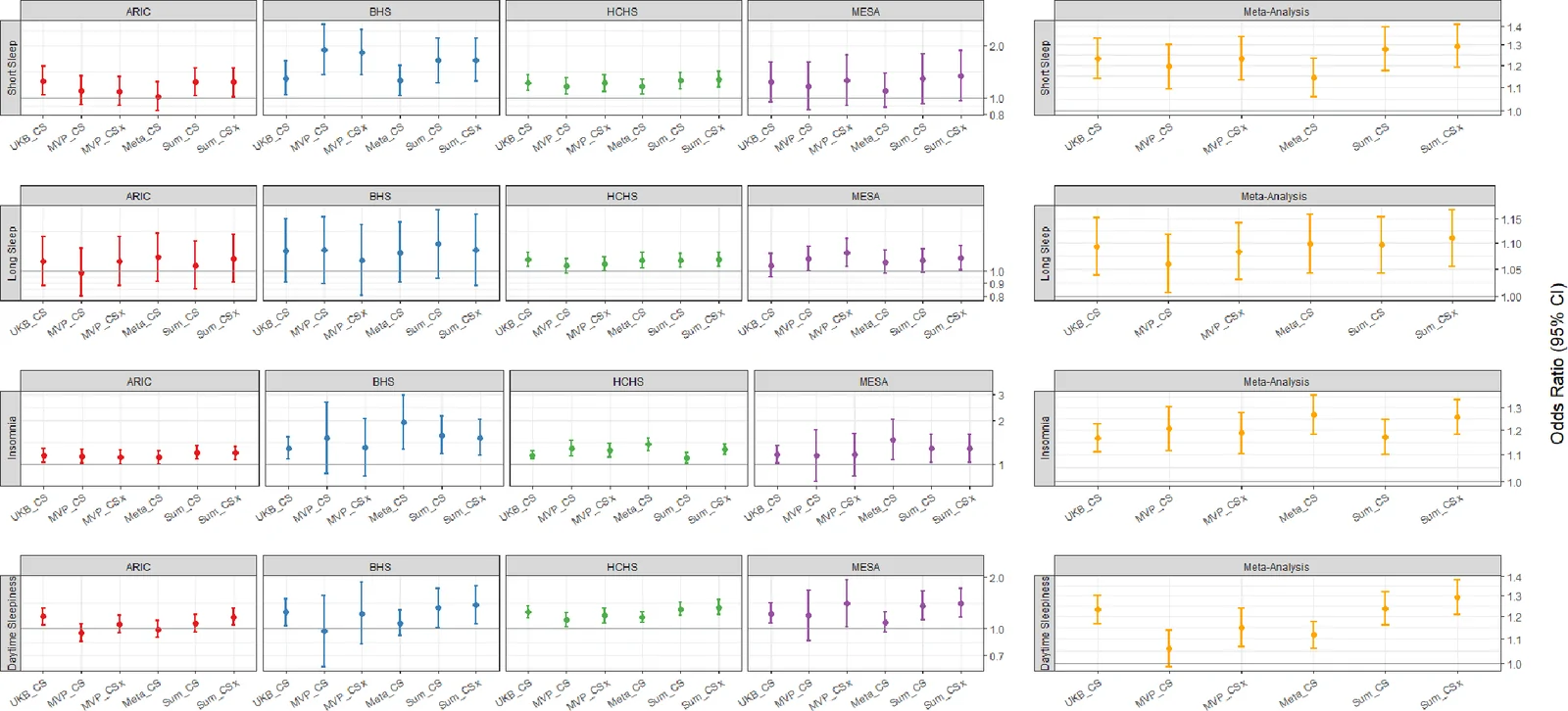
Comparison of associations between UKB and MVP sleep PRSs and selected sleep phenotype definitions for short sleep (≤6 hours), long sleep (≥9 hours), insomnia (WHIIRS 5 questions ≥10, WHIIRS 3 questions ≥6), and excessive daytime sleepiness (ESS ≥11). Study-specific results are presented for ARIC (red), BHS (blue), HCHS/SOL (green) and MESA (purple). Meta-analyses results are presented in orange. Results are presented for the following PRS: UKB CS, MVP CS, MVP CSx, Meta CS, Sum CS, and Sum CSx. Meta CS is the meta-analysis of UKB and MVP GWAS summary statistics followed by PRS-CS. Sum CS is the summation of UKB PRS and MVP PRS-CS. Sum CSx is the summation of UKB PRS and MVP PRS-CSx.

**Figure 4.**
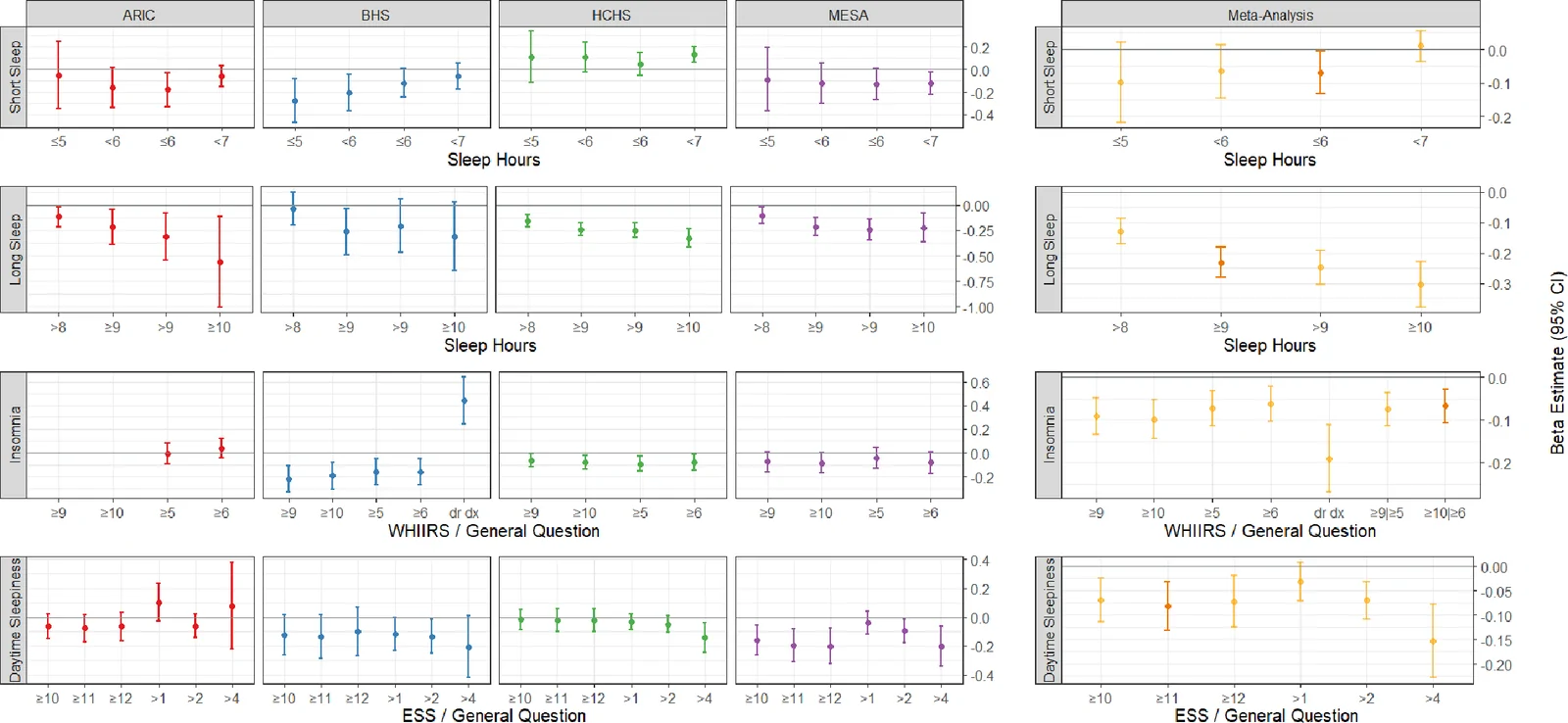
Study-specific and meta-analysis results for the association between sleep phenotype definitions for short sleep, long sleep, insomnia and daytime sleepiness and standardized scores from the digit symbol substitution test (DSST). Study-specific results are presented for ARIC (red), BHS (blue), HCHS/SOL (green) and MESA (purple). Meta-analyses are presented in orange, with bolded associations between sleep phenotypes and DSST for short sleep ≤6 hours, long sleep ≥9 hours, WHIIRS ≥10 (or WHIIRS ≥6 if only 3 questions available), and ESS ≥11.

**Figure 5.**
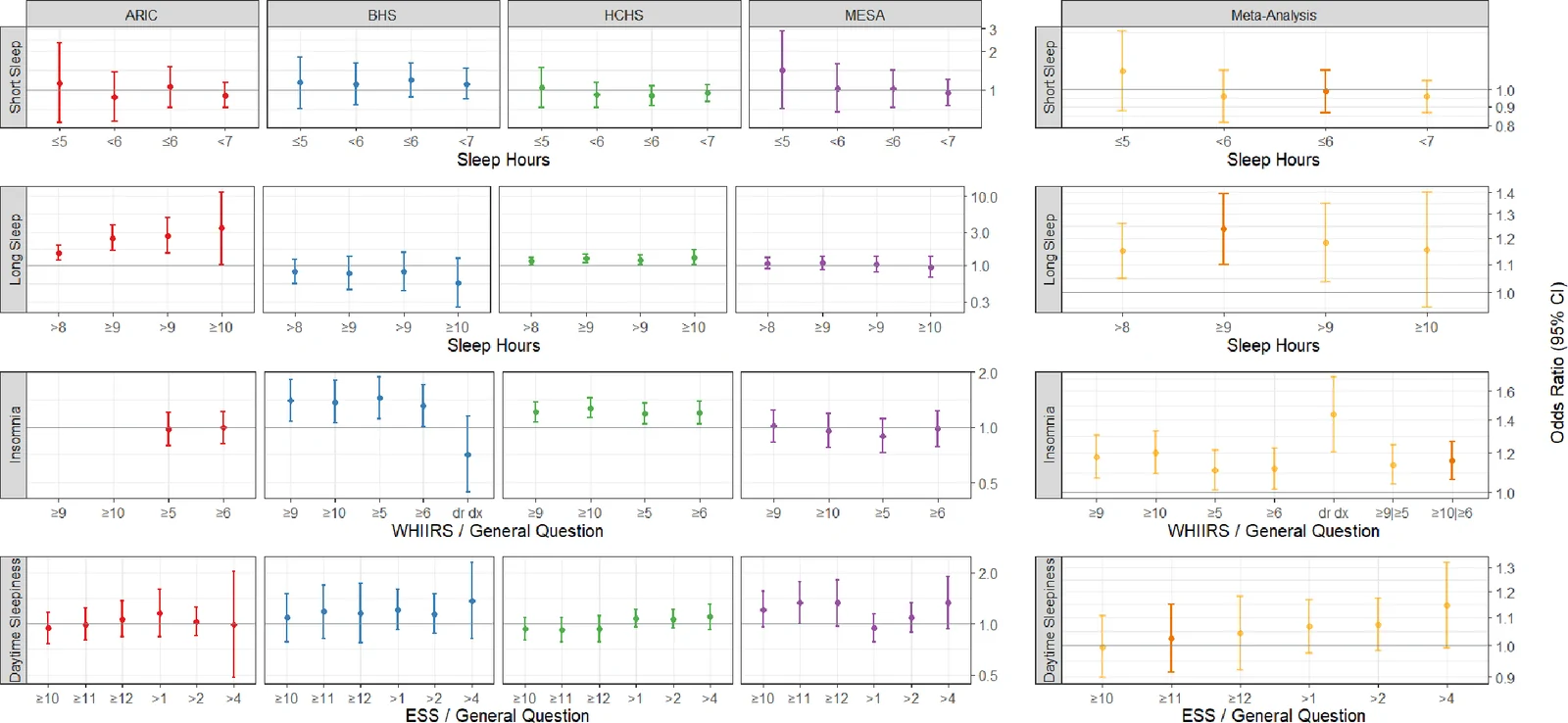
Study-specific and meta-analysis results for the association between sleep phenotype definitions for short sleep, long sleep, insomnia and daytime sleepiness and hypertension. Study-specific results are presented for ARIC (red), BHS (blue), HCHS/SOL (green) and MESA (purple). Meta-analyses are presented in orange, with bolded associations between sleep phenotypes and hypertension for short sleep ≤6 hours, long sleep ≥9 hours, WHIIRS ≥10 (or WHIIRS ≥6 if only 3 questions available), and ESS ≥11.

**Table 1.** Sleep questions from HCHS/SOL (visit 1), MESA (visits 5 and 4), and ARIC (visit 4)

Question	HCHS/SOL (Visit 1)	MESA (Visit 5)	MESA (Visit 4)	ARIC (Visit 4)	BHS (Biceps Visit)

**Sleep Duration**					
Weekday	What time do you usually go to bed?	What time do you usually go to bed?	X	What time do you usually fall asleep?	Not including naps, what time do you usually go to bed?
	What time do you usually wake up?	What time do you usually wake up?	X	What time do you usually wake up?	Not including naps, what time do you usually wake up?
Weekend	What time do you usually go to bed?	What time do you usually go to bed?	X	What time do you usually fall asleep?	Not including naps, what time do you usually go to bed?
	What time do you usually wake up?	What time do you usually wake up?	X	What time do you usually wake up?	Not including naps, what time do you usually wake up?
General	X	X	How much sleep do you usually get at night?	How many hours of sleep do you usually get at night?	How many hours of sleep do you usually get per night: On weekdays or workdays? On weekends?
**Insomnia**					
WHIIRS	How often have you experienced the situation in the past 4 weeks?				
	Trouble Falling Asleep	Trouble Falling Asleep	Trouble Falling Asleep	Trouble Falling Asleep	Trouble Falling Asleep
	Wake up earlier than planned	Wake up earlier than planned	Wake up earlier than planned	Wake up earlier than planned	Wake up earlier than planned
	Trouble getting back to sleep	Trouble getting back to sleep	Trouble getting back to sleep	Trouble getting back to sleep	Trouble getting back to sleep
	Wake up several times at night	Wake up several times at night	X	X	Wake up several times at night
	Overall typical night’s sleep	Overall typical night’s sleep	X	X	Overall typical night’s sleep
General	Have you ever been told by a doctor that you have insomnia?	Have you ever been told by a doctor that you have insomnia?	Have you ever been told by a doctor that you have insomnia?	X	Have you ever been told by a doctor or other health professionals that you have insomnia?
**Excessive Daytime Sleepiness**					
ESS	What is the chance that you would doze off or fall asleep (not just “feel tired”) in each of the following situations?				
	Reading	Reading	Reading	Reading	Reading
	Watching TV	Watching TV	Watching TV	Watching TV	Watching TV
	Sitting in public	Sitting in public	Sitting in public	Sitting in public	Sitting in public
	Riding as a passenger in a car	Riding as a passenger in a car	Riding as a passenger in a car	Riding as a passenger in a car	Riding as a passenger in a car
	Lying down to rest in the afternoon	Lying down to rest in the afternoon	Lying down to rest in the afternoon	Lying down to rest in the afternoon	Lying down to rest in the afternoon
	Talking to someone	Talking to someone	Talking to someone	Talking to someone	Talking to someone
	Sitting quietly after lunch	Sitting quietly after lunch	Sitting quietly after lunch	Sitting quietly after lunch	Sitting quietly after lunch
	Stopped in a car	Stopped in a car	Stopped in a car	Stopped in a car	Stopped in a car
General	In the past 4 weeks (month), did you feel excessively (overly) sleepy during the day?	In the past 4 weeks (month), did you feel excessively (overly) sleepy during the day?	In the past 4 weeks (month), did you feel excessively (overly) sleepy during the day?	In the past 4 weeks (month), did you feel excessively (overly) sleepy during the day?	In the past 4 weeks (month), did you feel excessively (overly) sleepy during the day?

**Table 2.** Study characteristics for individuals from HCHS/SOL, MESA and ARIC in sleep PRS and sleep phenotype analysis

	HCHS/SOL visit 1 (N = 12 182)		MESA visit 5 (N = 2085)		ARIC visit 4 (N = 1550)		BHS biceps visit (N = 1078)	

**Characteristics (continuous)**	**N**	**Mean (SD)**	**N**	**Mean (SD)**	**N**	**Mean (SD)**	**N**	**Mean (SD)**
**Age (years)**	12 182	46.1 (13.9)	2085	68.7 (9.2)	1550	62.7 (5.5)	1078	48.2 (5.2)
**BMI (kg/m** ^**2**^ **)** ^a^	12 151	29.8 (6.1)	2081	28.7 (5.6)	1548	28.7 (5.1)	1078	31.5 (7.8)
**Sleep duration (hours)** ^b^	11 721	7.9 (1.4)	2062	8.0 (1.4)	1510	7.4 (1.0)	991	7.1 (1.4)
**Insomnia WHIIRS** ^c^	11 981	7.1 (5.5)	2069	7.4 (4.4)	1539	4.9 (2.5)	1067	8.1 (5.3)
**Daytime sleepiness ESS** ^d^	12 125	5.8 (4.8)	2073	6.0 (4.1)	1516	8.3 (4.4)	1072	6.2 (4.6)
**DSST (unscaled)**	7101	33.9 (13.5)	1882	51.3 (19.1)	1428	48.6 (10.5)	1078	59.6 (17.3)
**Characteristics (categorical)**	**N**	**%**	**N**	**%**	**N**	**%**	**N**	**%**
**Sex**								
Male	5037	41.3	978	46.9	893	48.7	440	40.8
Female	7145	58.7	1107	53.1	940	51.3	638	59.2
**Short sleep (hours)**								
≤5	357	3.0	42	2.0	31	2.1	93	9.4
<6	836	7.1	108	5.2	81	5.4	147	14.8
≤6	1156	9.9	178	8.6	117	7.7	269	27.1
<7	2502	21.3	399	19.4	392	26.0	408	41.1
**Long sleep (hours)**								
>8	5089	43.4	904	43.8	328	21.7	140	14.1
≥9	2740	23.4	503	24.4	94	6.2	67	6.8
>9	2121	18.1	372	18.0	53	3.5	50	5.1
≥10	964	8.2	183	8.9	13	0.9	29	2.9
**Insomnia (WHIIRS)**								
≥9	4375	36.5	727	35.1			472	44.2
≥10	3837	32.0	604	29.2			410	38.4
3 Ques≥5	4089	34.1	563	27.2	555	36.1	552	51.5
3 Ques≥6	3630	30.3	484	23.4	799	51.9	461	43.0
General Ques^e^	1163	9.5	139	6.7			90	8.8
**Daytime sleepiness (ESS)**								
≥10	2377	19.6	393	19.0	534	35.2	213	19.9
≥11	1905	15.7	287	13.8	431	28.4	169	15.8
≥12	1555	12.8	225	10.9	326	21.5	132	12.3
General Ques>1^f^	6092	50.1	923	44.2	1368	89.5	631	58.7
General Ques>2	5187	42.7	726	34.9	722	47.3	391	36.4
General Ques>4	1333	11.0	173	8.3	32	2.1	75	7.0
**Hypertension**								
No	8380	68.7	830	42.6	1607	87.7	594	55.1
Yes	3802	31.3	1253	57.3	225	12.3	484	44.9
Missing	0	0.0	2	0.1	1	0.1	0	0.0

a Number of individuals missing BMI in HCHS/SOL was 31, in MESA was 4, in ARIC was 2

b Number of individuals missing sleep duration in HCHS/SOL was 461, in MESA was 23, in ARIC was 40

c Number of individuals missing WHIIRS in HCHS/SOL was 201, in MESA was 16, in ARIC was 11

d Number of individuals missing ESS in HCHS/SOL was 57, in MESA was 12, in ARIC was 34

e Insomnia general question: Have you ever been told by a doctor that you have insomnia?

f Daytime Sleepiness general question: In the past 4 weeks (month), did you feel excessively/overly sleepy during the day?

**Table 3.** Summary of associations between sleep PRS and consensus sleep definitions (statistical significance, consistency across studies, and sample sizes) and additional considerations (most significant association between sleep definitions and DSST or hypertension)

	Short sleep	Long sleep	Insomnia^a^	Daytime sleepiness

**UKB sleep PRS and sleep phenotype (main analysis with MESA 5)**	Significant sleep PRS-phenotype associations: ≤5 hours: OR = 1.31 and *p* = 1.11×10^−4^ <6 hours: OR = 1.22 and *p* = 6.53×10^−5^ ≤6 hours: OR = 1.23 and *p* = 1.80×10^−7^ <7 hours: OR = 1.17 and *p* = 1.13×10^−7^ Consistency across studies: ≤5 hours: Phet = 0.66 <6 hours: Phet = 0.90 ≤6 hours: Phet = 0.97 <7 hours: Phet = 0.57 Number of cases: ≤5 hours: 523 <6 hours: 1172 ≤ 6 hours: 1720 <7 hours: 3701	Significant sleep PRS-phenotype associations: >8 hours: OR = 1.06 and *p* = 2.82×10^−3^ ≥9 hours: OR = 1.09 and *p* = 6.76×10^−4^ >9 hours: OR = 1.07 and *p* = 0.03 Consistency across studies: >8 hours: Phet = 0.68 ≥9 hours: Phet = 0.77 >9 hours: Phet = 0.42 Number of cases: >8 hours: 6461 ≥9 hours: 3404 >9 hours:2596	Significant sleep PRS-phenotype associations: WHIIRS (5 questions) ≥9 or WHIIRS (3 questions) ≥5: OR = 1.16, *p* = 7.23×10^−10^ WHIIRS (5 questions) ≥10 or WHIIRS (3 questions) ≥6: OR = 1.17, *p* = 5.51×10^−10^ Dr Dx: OR = 1.11 and *p* = .02 Consistency across studies: WHIIRS (5 questions) ≥9 or WHIIRS (3 questions) ≥5: Phet = 0.55 WHIIRS (5 questions) ≥10 or WHIIRS (3 questions) ≥6: Phet = 0.69 Dr Dx: Phet = 0.27 Number of cases: WHIIRS (5 questions) ≥9 or WHIIRS (3 questions) ≥5: 6129 WHIIRS (5 questions) ≥10 or WHIIRS (3 questions) ≥6: 5650 Dr Dx: 1392	Significant sleep PRS-phenotype associations: ESS ≥ 10: OR = 1.20 and *p* = 3.04×10^−13^ ESS ≥ 11: OR = 1.23 and *p* = 1.83×10^−13^ ESS ≥12: OR = 1.26 and *p* = 6.62×10^−15^ Gen Ques >1: OR = 1.06, *p* = .01 Gen Ques >4: OR = 1.10, *p* = 8.16×10^−3^ Consistency across studies: ESS ≥ 10: Phet = 0.77 ESS ≥ 11: Phet = 0.83 ESS ≥ 12: Phet = 0.65 Gen Ques >1: Phet = 0.19 Gen Ques >4: Phet = 0.20 Number of cases: ESS ≥ 10: 3517 ESS ≥ 11: 2792 ESS ≥ 12: 2238 Gen Ques >1: 9014 Gen Ques >4: 1613
**MVP CS-x sleep PRS and sleep phenotype (main analysis with MESA 5)**	Significant Sleep PRS-Phenotype Associations: ≤5 hours: OR = 1.26 and *p* = 3.72×10^−3^ <6 hours: OR = 1.17 and *p* = 3.36×10^−3^ ≤6 hours: OR = 1.23 and *p* = 2.36×10^−6^ <7 hours: OR = 1.13 and *p* = 1.54×10^−4^ Consistency across studies: ≤5 hours: Phet = 0.16 <6 hours: Phet = 0.01 ≤6 hours: Phet = 0.04 <7 hours: Phet = 0.03	Significant Sleep PRS-Phenotype Associations: >8 hours: OR = 1.06 and *p* = 5.49×10^−3^ ≥9 hours: OR = 1.08 and *p* = 2.05×10^−3^ >9 hours: OR = 1.08 and *p* = 8.95×10^−3^ Consistency across studies: >8 hours: Phet = 0.26 ≥9 hours: Phet = 0.52 >9 hours: Phet = 0.67	Significant Sleep PRS-Phenotype Associations: WHIIRS (5 questions) ≥9 or WHIIRS (3 questions) ≥5: OR = 1.16, *p* = 6.65×10^−5^ WHIIRS (5 questions) ≥10 or WHIIRS (3 questions) ≥6: OR = 1.19, *p* = 3.11×10^−6^ Dr Dx: OR = 1.45 and *p* = 7.39×10^−7^ Consistency across studies: WHIIRS (5 questions) ≥9 or WHIIRS (3 questions) ≥5: Phet = 0.60 WHIIRS (5 questions) ≥10 or WHIIRS (3 questions) ≥6: Phet = 0.49 Dr Dx: Phet = 0.16	Significant Sleep PRS-Phenotype Associations: ESS ≥ 10: OR = 1.15 and *p* = 1.14×10^−4^ ESS ≥ 11: OR = 1.15 and *p* = 2.32×10^−4^ ESS ≥12: OR = 1.14 and *p* = 2.39×10^−3^ Gen Ques >1: OR = 1.12 and *p* = 2.17×10^−3^ Gen Ques >2: OR = 1.08 and *p* = .01 Consistency across studies: ESS ≥ 10: Phet = 0.03 ESS ≥ 11: Phet = 0.29 ESS ≥ 12: Phet = 0.28 Gen Ques >1: Phet = 0.44 Gen Ques >2: Phet = 0.08
**UKB + MVP CS-x sleep PRS and sleep phenotype (main analysis with MESA 5)**	Significant sleep PRS-phenotype associations ≤5 hours: OR = 1.36 and *p* = 1.24×10^−4^ <6 hours: OR = 1.25 and *p* = 3.35×10^−5^ ≤6 hours: OR = 1.30 and *p* = 2.14×10^−9^ <7 hours: OR = 1.19 and *p* = 4.57×10^−8^ Consistency across studies: ≤5 hours: Phet = 0.58 <6 hours: Phet = 0.38 ≤6 hours: Phet = 0.35 <7 hours: Phet = 0.11	Significant sleep PRS-phenotype associations: >8 hours: OR = 1.07 and *p* = 5.31×10^−4^ ≥9 hours: OR = 1.11 and *p* = 4.91×10^−5^ >9 hours: OR = 1.09 and *p* = 2.87×10^−3^ ≥10 hours: OR = 1.09 and *p* = .01 Consistency across studies: >8 hours: Phet = 0.36 ≥9 hours: Phet = 0.95 >9 hours: Phet = 0.83 ≥10 hours: Phet = 0.69	Significant sleep PRS-phenotype associations: WHIIRS (5 questions) ≥9 or WHIIRS (3 questions) ≥5: OR = 1.23, *p* = 1.19×10^−11^ WHIIRS (5 questions) ≥10 or WHIIRS (3 questions) ≥6: OR = 1.26, *p* = 5.08×10^−13^ Dr Dx: OR = 1.28 and *p* = 1.13×10^−4^ Consistency across studies: WHIIRS (5 questions) ≥9 or WHIIRS (3 questions) ≥5: Phet = 0.60 WHIIRS (5 questions) ≥10 or WHIIRS (3 questions) ≥6: Phet = 0.43 Dr Dx: Phet = 0.14	Significant sleep PRS-phenotype associations: ESS ≥ 10: OR = 1.27 and *p* = 1.84×10^−14^ ESS ≥ 11: OR = 1.30 and *p* = 3.83×10^−14^ ESS ≥12: OR = 1.31 and *p* = 5.06×10^−14^ Gen Ques >1: OR = 1.11 and *p* = 5.15×10^−4^ Gen Ques >2: OR = 1.07 and *p* = 9.33×10^−3^ Gen Ques >4: OR = 1.15 and *p* = 3.18×10^−3^ Consistency across studies: ESS ≥ 10: Phet = 0.13 ESS ≥ 11: Phet = 0.24 ESS ≥ 12: Phet = 0.17 Gen Ques >1: Phet = 0.57 Gen Ques >2: Phet = 0.24 Gen Ques >4: Phet = 0.84
**UKB sleep PRS and sleep phenotype (secondary analysis with MESA 4)**	≤6 hours: OR = 1.26 and *p* = 1.98×10^−13^	≥9 hours: OR = 1.12 and *p* = 1.63×10^−5^	WHIIRS (5 questions) ≥10 or WHIIRS (3 questions) ≥6: OR = 1.18, *p* = 8.64×10^−14^	ESS ≥12: OR = 1.27 and *p* = 7.69×10^−20^
**Sleep phenotype and DSST (secondary analysis with MESA 5)**	≤6 hours (random effects): *β* = −0.09 and *p* = .10	≥9 hours: *β* = −0.23 and *p* = 1.46×10^−19^	WHIIRS (5 questions) ≥9 or WHIIRS (3 questions) ≥5 (random effects): *β* = −0.09 and *p* = .03	ESS ≥11: *β* = −0.08 and *p* = 1.25×10^−3^
**Sleep phenotype and hypertension (secondary analysis with MESA 5)**	≤5 hours: OR = 1.12 and *p* = .36	≥9 hours (fixed effects): OR = 1.24 and *p* = 3.99×10^−4^ >8 hours (random effects): OR = 1.14 and *p* = .19	Dr Dx: OR = 1.44, *p* = 4.28×10^−5^ WHIIRS (5 questions) ≥10 or WHIIRS (3 questions) ≥6 (fixed effects): OR = 1.16, *p* = 1.17×10^−3^ WHIIRS (5 questions) ≥9 or WHIIRS (3 questions) ≥5 (random effects): OR = 1.13, *p* = .10	Gen Ques >4: OR = 1.15 and *p* = .06 ESS ≥12: OR = 1.04 and *p* = .51

## Data Availability

HCHS/SOL, MESA and ARIC genetic and phenotype data are available through application to the Data Base of Genotypes and Phenotypes (dbGAP; https://dbgap.ncbi.nlm.nih.gov). HCHS/SOL data are available in accessions phs000810 and phs000880. MESA data are available in accessions phs000209 and phs001416 (TOPMed WGS). ARIC data are available in accessions phs000280 and phs000090. Sleep data for HCHS/SOL, MESA and ARIC are also available from the National Sleep Research Resource (sleepdata.org). Data from the BHS are confidential to protect participant privacy. De-identified BHS data are available upon request from the BHS Steering Committee. BHS data requests can be submitted by email and are subject to approval. Polygenic scores variants and weights are available from the PGS catalog under publication ID PGP000836 and score IDs PGS019920-PGS019939. Example analysis code available on GitHub at https://github.com/tamartsi/PRS_sleep_consensus_definitions.

## Statement of Significance

Sleep disorders are associated with a variety of health outcomes, including cardiovascular disease, diabetes, and Alzheimer’s disease. Previous studies have identified numerous genetic variants associated with short and long sleep durations, insomnia and excessive daytime sleepiness (EDS), from which summary measures called polygenic risk scores (PRSs) have been developed. However, previous genome-wide association studies (GWAS) have been conducted in differing populations. Further, information on sleep is often self-reported via questionnaire and specific questions can vary across studies. Therefore, we sought to evaluate associations between multiple sleep PRSs and a variety of sleep definitions utilizing data from over 16 000 individuals from four cohort studies (HCHS/SOL, MESA ARIC, and BHS). In conjunction with demographic and environmental information, this analysis can help inform which sleep PRSs and sleep definitions to prioritize in future studies, especially for cross-study collaborations.
