# Supplementary material for: Using polygenic risk scores to evaluate definitions of self-reported sleep phenotypes across cohorts
Source: Sleep. Author manuscript; Available in PMC 2026 Oct 9. (PMC13652048; doi:10.1093/sleep/zsag200)
Supplement: Supplementary file 1 [file NIHMS2213360-supplement-Supplement.docx]

**Supplemental Table 1.** Characteristics of Genome-Wide Association Studies (GWAS) used for Polygenic Risk Score Construction

| **Trait** | **Paper** | **Population** | **Trait Question** |
| --- | --- | --- | --- |
| Sleep Duration  Short Sleep  Long Sleep | Dashti et al. 2019 *Nat Commun* | UK Biobank | About how many hours of sleep do you get in every 24h?  Short (<7) and Long (≥9) |
|  | Austin-Zimmerman et al 2023 *Nat Commun* | MVP | How many hours do you usually sleep each day (24-h period)?  Response options: 5 or less, 6, 7, 8, 9 or 10 or more  Short (≤5) and Long (≥ 10) |
| Insomnia | Jansen et al. 2019 *Nat Genet* | UK Biobank | Do you have trouble falling asleep at night or do you wake up in the middle of the night?  “1. Never/rarely”, “2. Sometimes”, “3. Usually”  Cases=3, Controls=1&2 |
|  | Verma et al 2024 *Science* | MVP | Patients with at least one ICD-9 or ICD-10 code are considered a case for the phenotype:  ICD-9 Diagnostic Codes: 307.41, 307.42, 327.0, 327.00, 327.01, 327.02, 327.09, 780.52  ICD-10 Diagnostic Codes: F51.01, F51.02, F51.03, F51.04, F51.05, F51.09, G47.0, G47.00, G47.01, G47.09 |
| Daytime Sleepiness | Wang et al. 2019 *Nat Commun* | UK Biobank | How likely are you to dose off or fall asleep during the daytime when you don’t mean to?  Response options: 1. Never/rarely, 2. Sometimes, 3. Often, 4. All of the time |
|  | Verma et al 2024 *Science* | MVP | Feeling excessively sleepy during the day (does not include regular naps)  Yes, No |

**Supplemental Table 2.** Study-Specific and Main Meta-Analysis Results for the Association between UKB Sleep PRSs and Sleep Phenotype Definitions for Short Sleep Long Sleep Insomnia and Daytime Sleepiness ARIC (Visit 4),

HCHS/SOL (Visit 1), BHS (Biceps) and MESA (Visit 5)

|  |  | **ARIC** | | | | | | | **HCHS/SOL** | | | | | | | **MESA 5** | | | | | | | **BHS** | | | | | | | | | | | | | | | |  |
| --- | --- | --- | --- | --- | --- | --- | --- | --- | --- | --- | --- | --- | --- | --- | --- | --- | --- | --- | --- | --- | --- | --- | --- | --- | --- | --- | --- | --- | --- | --- | --- | --- | --- | --- | --- | --- | --- | --- | --- |
| **Trait** | **Threshold** | **N** | **N cases** | **Beta** | **SE** | **OR** | **95% CI** | **Pvalue** | **N** | **N cases** | **Beta** | **SE** | **OR** | **95% CI** | **Pvalue** | **N** | **N cases** | **Beta** | **SE** | **OR** | **95% CI** | **Pvalue** | **N** | **N cases** | **Beta** | | | **SE** | | **OR** | | | | **95% CI** | | | **Pvalue** | |  |
| Short Sleep | ≤5 | 1491 | 31 | 0.16 | 0.19 | 1.17 | 0.82, 1.69 | 0.39 | 11721 | 357 | 0.34 | 0.09 | 1.41 | 1.18-1.67 | 1.44E-04 | 2062 | 42 | 0.20 | 0.28 | 1.23 | 0.71-2.11 | 0.46 | 991 | 93 | 0.14 | | | 0.17 | | 1.15 | | | | 0.82-1.61 | | | 0.43 | |  |
| Short Sleep | <6 | 1491 | 81 | 0.22 | 0.12 | 1.25 | 1.00, 1.57 | 0.05 | 11721 | 836 | 0.21 | 0.06 | 1.24 | 1.09-1.4 | 7.47E-04 | 2062 | 108 | 0.13 | 0.17 | 1.13 | 0.81-1.59 | 0.46 | 991 | 147 | 0.12 | | | 0.14 | | 1.13 | | | | 0.85-1.5 | | | 0.39 | |  |
| Short Sleep | ≤6 | 1491 | 117 | 0.22 | 0.10 | 1.25 | 1.03, 1.51 | 0.02 | 11721 | 1156 | 0.20 | 0.05 | 1.22 | 1.1-1.35 | 1.21E-04 | 2062 | 178 | 0.21 | 0.14 | 1.23 | 0.94-1.6 | 0.13 | 991 | 269 | 0.26 | | | 0.11 | | 1.30 | | | | 1.04-1.62 | | | 0.02 | |  |
| Short Sleep | <7 | 1491 | 392 | 0.13 | 0.06 | 1.13 | 1.01, 1.27 | 0.04 | 11721 | 2502 | 0.15 | 0.04 | 1.16 | 1.07-1.25 | 2.58E-04 | 2062 | 399 | 0.26 | 0.10 | 1.30 | 1.08-1.57 | 5.57E-03 | 991 | 408 | 0.22 | | | 0.10 | | 1.25 | | | | 1.02-1.53 | | | 0.03 | |  |
| Long Sleep | >8 | 1491 | 328 | -0.004 | 0.06 | 1.00 | 0.88, 1.13 | 0.96 | 11721 | 5089 | 0.08 | 0.03 | 1.08 | 1.03-1.13 | 3.60E-03 | 2062 | 904 | 0.05 | 0.04 | 1.05 | 0.96-1.15 | 0.28 | 991 | 140 | 0.08 | | | 0.10 | | 1.09 | | | | 0.89-1.32 | | | 0.40 | |  |
| Long Sleep | ≥9 | 1491 | 94 | 0.08 | 0.11 | 1.09 | 0.88, 1.35 | 0.45 | 11721 | 2740 | 0.10 | 0.03 | 1.11 | 1.04-1.18 | 1.94E-03 | 2062 | 503 | 0.05 | 0.05 | 1.05 | 0.95-1.16 | 0.35 | 991 | 67 | 0.18 | | | 0.14 | | 1.19 | | | | 0.9-1.58 | | | 0.22 | |  |
| Long Sleep | >9 | 1491 | 53 | 0.07 | 0.14 | 1.07 | 0.81, 1.42 | 0.61 | 11721 | 2121 | 0.09 | 0.04 | 1.10 | 1.02-1.18 | 8.69E-03 | 2062 | 372 | -0.02 | 0.06 | 0.98 | 0.87-1.1 | 0.73 | 991 | 50 | 0.07 | | | 0.17 | | 1.07 | | | | 0.77-1.49 | | | 0.67 | |  |
| Long Sleep | ≥10 | 1491 | 13 | -0.16 | 0.29 | 0.85 | 0.49, 1.49 | 0.58 | 11721 | 964 | 0.08 | 0.05 | 1.08 | 0.98-1.19 | 0.11 | 2062 | 183 | 0.09 | 0.08 | 1.09 | 0.94-1.28 | 0.26 | 991 | 29 | -0.06 | | | 0.22 | | 0.94 | | | | 0.61-1.45 | | | 0.78 | |  |
| Sleep Duration | Continuous | 1491 | NA | 0.09 | 0.03 | NA | NA | 3.23E-04 | 11721 | NA | 0.11 | 0.02 | NA | NA | 5.45E-08 | 2062 | NA | 0.14 | 0.04 | NA | NA | 6.13E-05 | 991 | NA | 0.11 | | | 0.05 | | NA | | | | NA | | | 0.04 | |  |
| Insomnia WHIIRS | ≥9 | NA | NA | NA | NA | NA | NA | NA | 11981 | 4375 | 0.14 | 0.03 | 1.15 | 1.08-1.22 | 1.42E-05 | 2069 | 727 | 0.12 | 0.07 | 1.13 | 0.99-1.28 | 0.07 | 1067 | 472 | 0.26 | | | 0.09 | | 1.30 | | | | 1.09-1.54 | | | 2.82E-03 | |  |
| Insomnia WHIIRS | ≥10 | NA | NA | NA | NA | NA | NA | NA | 11981 | 3837 | 0.14 | 0.03 | 1.15 | 1.08-1.23 | 1.23E-05 | 2069 | 604 | 0.16 | 0.07 | 1.17 | 1.02-1.34 | 0.02 | 1067 | 410 | 0.26 | | | 0.09 | | 1.29 | | | | 1.08-1.54 | | | 4.18E-03 | |  |
| Insomnia WHIIRS 3Q | ≥5 | 1520 | 555 | 0.16 | 0.06 | 1.17 | 1.05, 1.31 | 0.003 | 11999 | 4089 | 0.16 | 0.03 | 1.18 | 1.11-1.25 | 1.21E-07 | 2070 | 563 | 0.09 | 0.07 | 1.09 | 0.95-1.25 | 0.20 | 1071 | 552 | 0.25 | | | 0.09 | | 1.29 | | | | 1.08-1.52 | | | 3.91E-03 | |  |
| Insomnia WHIIRS 3Q | ≥6 | 1520 | 799 | 0.14 | 0.05 | 1.15 | 1.04, 1.28 | 0.007 | 11999 | 3630 | 0.15 | 0.03 | 1.16 | 1.09-1.24 | 6.05E-06 | 2070 | 484 | 0.10 | 0.07 | 1.11 | 0.96-1.28 | 0.17 | 1071 | 461 | 0.23 | | | 0.09 | | 1.26 | | | | 1.06-1.49 | | | 8.90E-03 | |  |
| Insomnia Dr Dx | Yes | NA | NA | NA | NA | NA | NA | NA | 12289 | 1163 | 0.07 | 0.05 | 1.07 | 0.97-1.18 | 0.16 | 2076 | 139 | 0.29 | 0.13 | 1.34 | 1.04-1.72 | 0.02 | 1024 | 90 | 0.15 | | | 0.16 | | 1.16 | | | | 0.86-1.58 | | | 0.33 | |  |
| Insomnia WHIIRS | Continuous | NA | NA | NA | NA | NA | NA | NA | 11981 | NA | 0.38 | 0.07 | NA | NA | 5.61E-08 | 2069 | NA | 0.33 | 0.14 | NA | NA | 0.02 | 1067 | NA | 0.71 | | | 0.22 | | NA | | | | NA | | | 1.57E-03 | |  |
| Insomnia WHIIRS 3Q | Continuous | 1520 | NA | 0.20 | 0.06 | NA | NA | 1.71E-03 | 11999 | NA | 0.24 | 0.05 | NA | NA | 8.67E-07 | 2070 | NA | 0.10 | 0.10 | NA | NA | 0.34 | 1071 | NA | 0.46 | | | 0.15 | | NA | | | | NA | | | 1.74E-03 | |  |
| Daytime Sleepiness ESS | ≥10 | 1497 | 534 | 0.19 | 0.06 | 1.21 | 1.08, 1.35 | 8.0E-04 | 12125 | 2377 | 0.16 | 0.03 | 1.18 | 1.1-1.26 | 3.23E-06 | 2073 | 393 | 0.21 | 0.06 | 1.23 | 1.1-1.39 | 4.44E-04 | 1072 | 213 | 0.25 | | | 0.08 | | 1.28 | | | | 1.09-1.51 | | | 3.06E-03 | |  |
| Daytime Sleepiness ESS | ≥11 | 1497 | 431 | 0.16 | 0.06 | 1.18 | 1.05, 1.32 | 0.006 | 12125 | 1905 | 0.23 | 0.04 | 1.26 | 1.16-1.36 | 2.12E-08 | 2073 | 287 | 0.21 | 0.07 | 1.23 | 1.08-1.41 | 2.32E-03 | 1072 | 169 | 0.22 | | | 0.09 | | 1.25 | | | | 1.04-1.5 | | | 0.02 | |  |
| Daytime Sleepiness ESS | ≥12 | 1497 | 326 | 0.17 | 0.06 | 1.19 | 1.05, 1.35 | 0.006 | 12125 | 1555 | 0.26 | 0.04 | 1.30 | 1.2-1.41 | 3.40E-10 | 2073 | 225 | 0.20 | 0.08 | 1.22 | 1.06-1.42 | 7.02E-03 | 1072 | 132 | 0.27 | | | 0.10 | | 1.31 | | | | 1.07-1.6 | | | 0.01 | |  |
| Daytime Sleepiness GenQ | >1 | 1528 | 1368 | -0.03 | 0.09 | 0.97 | 0.82, 1.15 | 0.76 | 12159 | 6092 | 0.06 | 0.03 | 1.07 | 1.01-1.13 | 0.03 | 2081 | 923 | 0.01 | 0.05 | 1.01 | 0.92-1.1 | 0.90 | 1075 | 631 | 0.16 | | | 0.07 | | 1.18 | | | | 1.03-1.34 | | | 0.01 | |  |
| Daytime Sleepiness GenQ | >2 | 1528 | 722 | 0.04 | 0.05 | 1.04 | 0.94, 1.15 | 0.44 | 12159 | 5187 | 0.06 | 0.03 | 1.06 | 1-1.12 | 0.05 | 2081 | 726 | -0.04 | 0.05 | 0.96 | 0.87-1.05 | 0.36 | 1075 | 391 | 0.10 | | | 0.07 | | 1.11 | | | | 0.97-1.26 | | | 0.13 | |  |
| Daytime Sleepiness GenQ | >4 | 1528 | 32 | 0.24 | 0.18 | 1.27 | 0.89, 1.80 | 0.19 | 12159 | 1333 | 0.11 | 0.05 | 1.12 | 1.02-1.22 | 0.01 | 2081 | 173 | -0.04 | 0.08 | 0.96 | 0.82-1.13 | 0.65 | 1075 | 75 | 0.24 | | | 0.13 | | 1.27 | | | | 0.99-1.63 | | | 0.06 | |  |
| Daytime Sleepiness ESS | Continuous | 1497 | NA | 0.50 | 0.11 | NA | NA | 1.06E-05 | 12125 | NA | 0.47 | 0.06 | NA | NA | 1.20E-12 | 2073 | NA | 0.43 | 0.09 | NA | NA | 2.38E-06 | 1072 | NA | 0.48 | | | 0.15 | | NA | | | | NA | | | 1.05E-03 | |  |
|  |  | **Meta-Analysis (Fixed Effects)** | | | | | | | |  |  |  |  |  |  |  |  |  |  |  |  |  |  |  |  | |  | | | |  | |  | | |  | | |  |
|  |  |  |  | **Beta** | **SE** | **OR** | **95% CI** | **Pvalue** | **P Heterogeneity** |  |  |  |  |  |  |  |  |  |  |  |  |  |  |  | |  | | |  | | |  | | |  | | |  | |
| Short Sleep | ≤5 |  |  | 0.27 | 0.07 | 1.31 | 1.14-1.51 | 1.11E-04 | 0.66 |  |  |  |  |  |  |  |  |  |  |  |  |  |  |  | |  | | |  | | |  | | |  | | |  | |
| Short Sleep | <6 |  |  | 0.20 | 0.05 | 1.22 | 1.11-1.34 | 6.53E-05 | 0.90 |  |  |  |  |  |  |  |  |  |  |  |  |  |  |  | |  | | |  | | |  | | |  | | |  | |
| Short Sleep | ≤6 |  |  | 0.21 | 0.04 | 1.23 | 1.14-1.33 | 1.80E-07 | 0.97 |  |  |  |  |  |  |  |  |  |  |  |  |  |  |  | |  | | |  | | |  | | |  | | |  | |
| Short Sleep | <7 |  |  | 0.16 | 0.03 | 1.17 | 1.11-1.24 | 1.13E-07 | 0.57 |  |  |  |  |  |  |  |  |  |  |  |  |  |  |  | |  | | |  | | |  | | |  | | |  | |
| Long Sleep | >8 |  |  | 0.06 | 0.02 | 1.06 | 1.02-1.11 | 2.82E-03 | 0.68 |  |  |  |  |  |  |  |  |  |  |  |  |  |  |  | |  | | |  | | |  | | |  | | |  | |
| Long Sleep | ≥9 |  |  | 0.09 | 0.03 | 1.09 | 1.04-1.15 | 6.76E-04 | 0.77 |  |  |  |  |  |  |  |  |  |  |  |  |  |  |  | |  | | |  | | |  | | |  | | |  | |
| Long Sleep | >9 |  |  | 0.06 | 0.03 | 1.07 | 1.01-1.13 | 0.03 | 0.42 |  |  |  |  |  |  |  |  |  |  |  |  |  |  |  | |  | | |  | | |  | | |  | | |  | |
| Long Sleep | ≥10 |  |  | 0.07 | 0.04 | 1.07 | 0.99-1.16 | 0.08 | 0.78 |  |  |  |  |  |  |  |  |  |  |  |  |  |  |  | |  | | |  | | |  | | |  | | |  | |
| Sleep Duration | Continuous |  |  | 0.11 | 0.01 | NA | NA | 2.60E-15 | 0.69 |  |  |  |  |  |  |  |  |  |  |  |  |  |  |  | |  | | |  | | |  | | |  | | |  | |
| Insomnia WHIIRS | ≥9 |  |  | 0.15 | 0.03 | 1.16 | 1.1-1.22 | 5.72E-08 | 0.37 |  |  |  |  |  |  |  |  |  |  |  |  |  |  |  | |  | | |  | | |  | | |  | | |  | |
| Insomnia WHIIRS | ≥10 |  |  | 0.16 | 0.03 | 1.17 | 1.11-1.24 | 2.13E-08 | 0.49 |  |  |  |  |  |  |  |  |  |  |  |  |  |  |  | |  | | |  | | |  | | |  | | |  | |
| Insomnia WHIIRS 3Q | ≥5 |  |  | 0.16 | 0.02 | 1.18 | 1.12-1.23 | 1.89E-11 | 0.54 |  |  |  |  |  |  |  |  |  |  |  |  |  |  |  | |  | | |  | | |  | | |  | | |  | |
| Insomnia WHIIRS 3Q | ≥6 |  |  | 0.15 | 0.02 | 1.16 | 1.1-1.22 | 2.60E-09 | 0.73 |  |  |  |  |  |  |  |  |  |  |  |  |  |  |  | |  | | |  | | |  | | |  | | |  | |
| Insomnia WHIIRS 5Q, 3Q | ≥9, ≥5 |  |  | 0.15 | 0.02 | 1.16 | 1.11-1.22 | 7.23E-10 | 0.55 |  |  |  |  |  |  |  |  |  |  |  |  |  |  |  | |  | | |  | | |  | | |  | | |  | |
| Insomnia WHIIRS 5Q, 3Q | ≥10, ≥6 |  |  | 0.15 | 0.02 | 1.17 | 1.11-1.22 | 5.51E-10 | 0.69 |  |  |  |  |  |  |  |  |  |  |  |  |  |  |  | |  | | |  | | |  | | |  | | |  | |
| Insomnia Dr Dx | Yes |  |  | 0.10 | 0.04 | 1.11 | 1.02-1.21 | 0.02 | 0.27 |  |  |  |  |  |  |  |  |  |  |  |  |  |  |  | |  | | |  | | |  | | |  | | |  | |
| Insomnia WHIIRS | Continuous |  |  | 0.40 | 0.06 | NA | NA | 3.70E-11 | 0.32 |  |  |  |  |  |  |  |  |  |  |  |  |  |  |  | |  | | |  | | |  | | |  | | |  | |
| Insomnia WHIIRS 3Q | Continuous |  |  | 0.22 | 0.03 | NA | NA | 1.75E-10 | 0.20 |  |  |  |  |  |  |  |  |  |  |  |  |  |  |  | |  | | |  | | |  | | |  | | |  | |
| Daytime Sleepiness ESS | ≥10 |  |  | 0.18 | 0.03 | 1.20 | 1.14-1.26 | 3.04E-13 | 0.77 |  |  |  |  |  |  |  |  |  |  |  |  |  |  |  | |  | | |  | | |  | | |  | | |  | |
| Daytime Sleepiness ESS | ≥11 |  |  | 0.21 | 0.03 | 1.23 | 1.16-1.3 | 1.83E-13 | 0.83 |  |  |  |  |  |  |  |  |  |  |  |  |  |  |  | |  | | |  | | |  | | |  | | |  | |
| Daytime Sleepiness ESS | ≥12 |  |  | 0.23 | 0.03 | 1.26 | 1.19-1.34 | 6.62E-15 | 0.65 |  |  |  |  |  |  |  |  |  |  |  |  |  |  |  | |  | | |  | | |  | | |  | | |  | |
| Daytime Sleepiness GenQ | >1 |  |  | 0.06 | 0.02 | 1.06 | 1.01-1.1 | 1.38E-02 | 0.19 |  |  |  |  |  |  |  |  |  |  |  |  |  |  |  | |  | | |  | | |  | | |  | | |  | |
| Daytime Sleepiness GenQ | >2 |  |  | 0.04 | 0.02 | 1.04 | 1-1.08 | 0.06 | 0.23 |  |  |  |  |  |  |  |  |  |  |  |  |  |  |  | |  | | |  | | |  | | |  | | |  | |
| Daytime Sleepiness GenQ | >4 |  |  | 0.10 | 0.04 | 1.10 | 1.03-1.19 | 8.16E-03 | 0.20 |  |  |  |  |  |  |  |  |  |  |  |  |  |  |  | |  | | |  | | |  | | |  | | |  | |
| Daytime Sleepiness ESS | Continuous |  |  | 0.47 | 0.05 | NA | NA | 1.24E-24 | 0.97 |  |  |  |  |  |  |  |  |  |  |  |  |  |  |  | |  | | |  | | |  | | |  | | |  | |

Beta and SE (standard error) for log odds ratio, 95% CI (confidence interval) for OR (odds ratio)

3Q = 3 questions, Dr Dx = Self-reported doctor diagnosis, GenQ = General question

**Supplemental Table 3.** Study-Specific and Secondary Meta-Analysis Results for the Association between Sleep PRSs and Sleep Phenotype Definitions for Short Sleep Long Sleep Insomnia and Daytime Sleepiness, ARIC (Visit 4),

HCHS/SOL (Visit 1) BHS (Biceps) and MESA (Visit 4)

|  |  | **ARIC** | | | | | | | **HCHS/SOL** | | | | | | | **MESA 4** | | | | | | | **BHS** | | | | | | | | | | | |  |
| --- | --- | --- | --- | --- | --- | --- | --- | --- | --- | --- | --- | --- | --- | --- | --- | --- | --- | --- | --- | --- | --- | --- | --- | --- | --- | --- | --- | --- | --- | --- | --- | --- | --- | --- | --- |
| **Trait** | **Threshold** | **N** | **N cases** | **Beta** | **SE** | **OR** | **95% CI** | **Pvalue** | **N** | **N cases** | **Beta** | **SE** | **OR** | **95% CI** | **Pvalue** | **N** | **N cases** | **Beta** | **SE** | **OR** | **95% CI** | **Pvalue** | **N** | **N cases** | **Beta** | | **SE** | | **OR** | | **95% CI** | | **Pvalue** | |  |
| Short Sleep | ≤5 | 1491 | 31 | 0.16 | 0.19 | 1.17 | 0.82, 1.69 | 0.39 | 11721 | 357 | 0.34 | 0.09 | 1.41 | 1.18-1.67 | 1.44E-04 | 5305 | 769 | 0.33 | 0.07 | 1.40 | 1.23-1.59 | 4.20E-07 | 991 | 93 | 0.14 | | 0.17 | | 1.15 | | 0.82-1.61 | | 0.43 | |  |
| Short Sleep | <6 | 1491 | 81 | 0.22 | 0.12 | 1.25 | 1.00, 1.57 | 0.05 | 11721 | 836 | 0.21 | 0.06 | 1.24 | 1.09-1.4 | 7.47E-04 | NA | NA | NA | NA | NA | NA | NA | 991 | 147 | 0.12 | | 0.14 | | 1.13 | | 0.85-1.5 | | 0.39 | |  |
| Short Sleep | ≤6 | 1491 | 117 | 0.22 | 0.10 | 1.25 | 1.03, 1.51 | 0.02 | 11721 | 1156 | 0.20 | 0.05 | 1.22 | 1.1-1.35 | 1.21E-04 | 5305 | 2111 | 0.26 | 0.05 | 1.29 | 1.18-1.42 | 5.34E-08 | 991 | 269 | 0.26 | | 0.11 | | 1.30 | | 1.04-1.62 | | 0.02 | |  |
| Short Sleep | <7 | 1491 | 392 | 0.13 | 0.06 | 1.13 | 1.01, 1.27 | 0.04 | 11721 | 2502 | 0.15 | 0.04 | 1.16 | 1.07-1.25 | 2.58E-04 | NA | NA | NA | NA | NA | NA | NA | 991 | 408 | 0.22 | | 0.10 | | 1.25 | | 1.02-1.53 | | 0.03 | |  |
| Long Sleep | >8 | 1491 | 328 | -0.004 | 0.06 | 1.00 | 0.88, 1.13 | 0.96 | 11721 | 5089 | 0.08 | 0.03 | 1.08 | 1.03-1.13 | 3.60E-03 | NA | NA | NA | NA | NA | NA | NA | 991 | 140 | 0.08 | | 0.10 | | 1.09 | | 0.89-1.32 | | 0.40 | |  |
| Long Sleep | ≥9 | 1491 | 94 | 0.08 | 0.11 | 1.09 | 0.88, 1.35 | 0.45 | 11721 | 2740 | 0.10 | 0.03 | 1.11 | 1.04-1.18 | 1.94E-03 | 5305 | 319 | 0.16 | 0.06 | 1.18 | 1.05-1.32 | 4.85E-03 | 991 | 67 | 0.18 | | 0.14 | | 1.19 | | 0.9-1.58 | | 0.22 | |  |
| Long Sleep | >9 | 1491 | 53 | 0.07 | 0.14 | 1.07 | 0.81, 1.42 | 0.61 | 11721 | 2121 | 0.09 | 0.04 | 1.10 | 1.02-1.18 | 8.69E-03 | NA | NA | NA | NA | NA | NA | NA | 991 | 50 | 0.07 | | 0.17 | | 1.07 | | 0.77-1.49 | | 0.67 | |  |
| Long Sleep | ≥10 | 1491 | 13 | -0.16 | 0.29 | 0.85 | 0.49, 1.49 | 0.58 | 11721 | 964 | 0.08 | 0.05 | 1.08 | 0.98-1.19 | 0.11 | 5305 | 98 | 0.27 | 0.10 | 1.31 | 1.07-1.6 | 0.01 | 991 | 29 | -0.06 | | 0.22 | | 0.94 | | 0.61-1.45 | | 0.78 | |  |
| Insomnia WHIIRS | ≥9 | NA | NA | NA | NA | NA | NA | NA | 11981 | 4375 | 0.14 | 0.03 | 1.15 | 1.08-1.22 | 1.42E-05 | NA | NA | NA | NA | NA | NA | NA | 1067 | 472 | 0.26 | | 0.09 | | 1.30 | | 1.09-1.54 | | 2.82E-03 | |  |
| Insomnia WHIIRS | ≥10 | NA | NA | NA | NA | NA | NA | NA | 11981 | 3837 | 0.14 | 0.03 | 1.15 | 1.08-1.23 | 1.23E-05 | NA | NA | NA | NA | NA | NA | NA | 1067 | 410 | 0.26 | | 0.09 | | 1.29 | | 1.08-1.54 | | 4.18E-03 | |  |
| Insomnia WHIIRS 3Q | ≥5 | 1520 | 555 | 0.16 | 0.06 | 1.17 | 1.05, 1.31 | 0.003 | 11999 | 4089 | 0.16 | 0.03 | 1.18 | 1.11-1.25 | 1.21E-07 | 5316 | 2200 | 0.15 | 0.04 | 1.17 | 1.08-1.26 | 8.10E-05 | 1071 | 552 | 0.25 | | 0.09 | | 1.29 | | 1.08-1.52 | | 3.91E-03 | |  |
| Insomnia WHIIRS 3Q | ≥6 | 1520 | 799 | 0.14 | 0.05 | 1.15 | 1.04, 1.28 | 0.007 | 11999 | 3630 | 0.15 | 0.03 | 1.16 | 1.09-1.24 | 6.05E-06 | 5316 | 1642 | 0.20 | 0.04 | 1.22 | 1.12-1.32 | 1.48E-06 | 1071 | 461 | 0.23 | | 0.09 | | 1.26 | | 1.06-1.49 | | 8.90E-03 | |  |
| Insomnia Dr Dx | Yes | NA | NA | NA | NA | NA | NA | NA | 12289 | 1163 | 0.07 | 0.05 | 1.07 | 0.97-1.18 | 0.16 | 5229 | 267 | 0.32 | 0.09 | 1.38 | 1.17-1.64 | 1.61E-04 | 1024 | 90 | 0.15 | | 0.16 | | 1.16 | | 0.86-1.58 | | 0.33 | |  |
| Daytime Sleepiness ESS | ≥10 | 1497 | 534 | 0.19 | 0.06 | 1.21 | 1.08, 1.35 | 8.0E-04 | 12125 | 2377 | 0.16 | 0.03 | 1.18 | 1.1-1.26 | 3.23E-06 | 5312 | 1156 | 0.21 | 0.04 | 1.24 | 1.15-1.33 | 1.87E-09 | 1072 | 213 | 0.25 | | 0.08 | | 1.28 | | 1.09-1.51 | | 3.06E-03 | |  |
| Daytime Sleepiness ESS | ≥11 | 1497 | 431 | 0.16 | 0.06 | 1.18 | 1.05, 1.32 | 0.006 | 12125 | 1905 | 0.23 | 0.04 | 1.26 | 1.16-1.36 | 2.12E-08 | 5312 | 889 | 0.21 | 0.04 | 1.24 | 1.15-1.34 | 4.76E-08 | 1072 | 169 | 0.22 | | 0.09 | | 1.25 | | 1.04-1.5 | | 0.02 | |  |
| Daytime Sleepiness ESS | ≥12 | 1497 | 326 | 0.17 | 0.06 | 1.19 | 1.05, 1.35 | 0.006 | 12125 | 1555 | 0.26 | 0.04 | 1.30 | 1.2-1.41 | 3.40E-10 | 5312 | 686 | 0.24 | 0.04 | 1.27 | 1.16-1.38 | 5.64E-08 | 1072 | 132 | 0.27 | | 0.10 | | 1.31 | | 1.07-1.6 | | 0.01 | |  |
| Daytime Sleepiness GenQ | >1 | 1528 | 1368 | -0.03 | 0.09 | 0.97 | 0.82, 1.15 | 0.76 | 12159 | 6092 | 0.06 | 0.03 | 1.07 | 1.01-1.13 | 0.03 | 5316 | 4039 | 0.11 | 0.04 | 1.12 | 1.04-1.2 | 1.62E-03 | 1075 | 631 | 0.16 | | 0.07 | | 1.18 | | 1.03-1.34 | | 0.01 | |  |
| Daytime Sleepiness GenQ | >2 | 1528 | 722 | 0.04 | 0.05 | 1.04 | 0.94, 1.15 | 0.44 | 12159 | 5187 | 0.06 | 0.03 | 1.06 | 1-1.12 | 0.05 | 5316 | 2476 | 0.07 | 0.03 | 1.08 | 1.02-1.14 | 9.55E-03 | 1075 | 391 | 0.10 | | 0.07 | | 1.11 | | 0.97-1.26 | | 0.13 | |  |
| Daytime Sleepiness GenQ | >4 | 1528 | 32 | 0.24 | 0.18 | 1.27 | 0.89, 1.80 | 0.19 | 12159 | 1333 | 0.11 | 0.05 | 1.12 | 1.02-1.22 | 0.01 | 5316 | 247 | 0.05 | 0.07 | 1.05 | 0.92-1.2 | 0.48 | 1075 | 75 | 0.24 | | 0.13 | | 1.27 | | 0.99-1.63 | | 0.06 | |  |
|  |  | **Meta-Analysis (Fixed Effects)** | | | | | | | |  |  |  |  |  |  |  |  |  |  |  |  |  |  |  | |  | |  | |  | |  | |  | |
|  |  |  |  | **Beta** | **SE** | **OR** | **95% CI** | **Pvalue** | **P Heterogeneity** |  |  |  |  |  |  |  |  |  |  |  |  |  |  |  | |  | |  | |  | |  | |  | |
| Short Sleep | ≤5 |  |  | 0.31 | 0.05 | 1.36 | 1.24-1.5 | 2.95E-10 | 0.59 |  |  |  |  |  |  |  |  |  |  |  |  |  |  |  | |  | |  | |  | |  | |  | |
| Short Sleep | <6 |  |  | 0.20 | 0.05 | 1.23 | 1.11-1.36 | 7.86E-05 | 0.83 |  |  |  |  |  |  |  |  |  |  |  |  |  |  |  | |  | |  | |  | |  | |  | |
| Short Sleep | ≤6 |  |  | 0.23 | 0.03 | 1.26 | 1.18-1.34 | 1.98E-13 | 0.85 |  |  |  |  |  |  |  |  |  |  |  |  |  |  |  | |  | |  | |  | |  | |  | |
| Short Sleep | <7 |  |  | 0.15 | 0.03 | 1.16 | 1.09-1.23 | 3.04E-06 | 0.72 |  |  |  |  |  |  |  |  |  |  |  |  |  |  |  | |  | |  | |  | |  | |  | |
| Long Sleep | >8 |  |  | 0.06 | 0.02 | 1.07 | 1.02-1.12 | 5.08E-03 | 0.50 |  |  |  |  |  |  |  |  |  |  |  |  |  |  |  | |  | |  | |  | |  | |  | |
| Long Sleep | ≥9 |  |  | 0.12 | 0.03 | 1.12 | 1.07-1.18 | 1.63E-05 | 0.76 |  |  |  |  |  |  |  |  |  |  |  |  |  |  |  | |  | |  | |  | |  | |  | |
| Long Sleep | >9 |  |  | 0.09 | 0.03 | 1.10 | 1.03-1.17 | 6.77E-03 | 0.98 |  |  |  |  |  |  |  |  |  |  |  |  |  |  |  | |  | |  | |  | |  | |  | |
| Long Sleep | ≥10 |  |  | 0.10 | 0.04 | 1.11 | 1.02-1.2 | 0.02 | 0.25 |  |  |  |  |  |  |  |  |  |  |  |  |  |  |  | |  | |  | |  | |  | |  | |
| Insomnia WHIIRS | ≥9 |  |  | 0.15 | 0.03 | 1.16 | 1.1-1.23 | 3.03E-07 | 0.18 |  |  |  |  |  |  |  |  |  |  |  |  |  |  |  | |  | |  | |  | |  | |  | |
| Insomnia WHIIRS | ≥10 |  |  | 0.16 | 0.03 | 1.17 | 1.1-1.24 | 3.05E-07 | 0.24 |  |  |  |  |  |  |  |  |  |  |  |  |  |  |  | |  | |  | |  | |  | |  | |
| Insomnia WHIIRS 3Q | ≥5 |  |  | 0.17 | 0.02 | 1.18 | 1.13-1.23 | 9.46E-15 | 0.78 |  |  |  |  |  |  |  |  |  |  |  |  |  |  |  | |  | |  | |  | |  | |  | |
| Insomnia WHIIRS 3Q | ≥6 |  |  | 0.17 | 0.02 | 1.18 | 1.13-1.23 | 5.99E-14 | 0.65 |  |  |  |  |  |  |  |  |  |  |  |  |  |  |  | |  | |  | |  | |  | |  | |
| Insomnia WHIIRS 5Q, 3Q | ≥9, ≥5 |  |  | 0.15 | 0.02 | 1.16 | 1.12-1.22 | 1.32E-12 | 0.60 |  |  |  |  |  |  |  |  |  |  |  |  |  |  |  | |  | |  | |  | |  | |  | |
| Insomnia WHIIRS 5Q, 3Q | ≥10, ≥6 |  |  | 0.17 | 0.02 | 1.18 | 1.13-1.23 | 8.64E-14 | 0.51 |  |  |  |  |  |  |  |  |  |  |  |  |  |  |  | |  | |  | |  | |  | |  | |
| Insomnia Dr Dx | Yes |  |  | 0.14 | 0.04 | 1.15 | 1.06-1.24 | 1.06E-03 | 0.04 |  |  |  |  |  |  |  |  |  |  |  |  |  |  |  | |  | |  | |  | |  | |  | |
| Daytime Sleepiness ESS | ≥10 |  |  | 0.19 | 0.02 | 1.21 | 1.16-1.26 | 2.09E-18 | 0.69 |  |  |  |  |  |  |  |  |  |  |  |  |  |  |  | |  | |  | |  | |  | |  | |
| Daytime Sleepiness ESS | ≥11 |  |  | 0.21 | 0.02 | 1.23 | 1.18-1.29 | 5.45E-18 | 0.83 |  |  |  |  |  |  |  |  |  |  |  |  |  |  |  | |  | |  | |  | |  | |  | |
| Daytime Sleepiness ESS | ≥12 |  |  | 0.24 | 0.03 | 1.27 | 1.21-1.34 | 7.69E-20 | 0.69 |  |  |  |  |  |  |  |  |  |  |  |  |  |  |  | |  | |  | |  | |  | |  | |
| Daytime Sleepiness GenQ | >1 |  |  | 0.09 | 0.02 | 1.09 | 1.05-1.13 | 4.47E-05 | 0.25 |  |  |  |  |  |  |  |  |  |  |  |  |  |  |  | |  | |  | |  | |  | |  | |
| Daytime Sleepiness GenQ | >2 |  |  | 0.07 | 0.02 | 1.07 | 1.03-1.11 | 3.23E-04 | 0.88 |  |  |  |  |  |  |  |  |  |  |  |  |  |  |  | |  | |  | |  | |  | |  | |
| Daytime Sleepiness GenQ | >4 |  |  | 0.11 | 0.04 | 1.11 | 1.04-1.19 | 2.02E-03 | 0.48 |  |  |  |  |  |  |  |  |  |  |  |  |  |  |  | |  | |  | |  | |  | |  | |

Beta and SE (standard error) for log odds ratio, 95% CI (confidence interval) for OR (odds ratio)

3Q = 3 questions, Dr Dx = Self-reported doctor diagnosis, GenQ = General question

**Supplemental Table 4**. BMI-Unadjusted and -Adjusted Study-Specific and Meta-Analysis Results for the Association between Sleep

PRSs and Sleep Phenotype Definitions for Short Sleep Long Sleep Insomnia and Daytime Sleepiness, ARIC (Visit 4), HCHS/SOL (Visit 1)

MESA (Visits 4 and 5), and BHS

|  |  | **ARIC** | | **HCHS/SOL** | | **MESA 4** | | **MESA 5** | | **BHS** | |
| --- | --- | --- | --- | --- | --- | --- | --- | --- | --- | --- | --- |
| **Trait** | **Outcome** | **OR** | **OR Adj BMI** | **OR** | **OR Adj BMI** | **OR** | **OR Adj BMI** | **OR** | **OR Adj BMI** | **OR** | **OR Adj BMI** |
| Short Sleep | ≤5 | 1.17 | 1.16 | 1.41 | 1.38 | 1.40 | 1.38 | 1.23 | 1.24 | 1.15 | 1.15 |
| Short Sleep | <6 | 1.25 | 1.24 | 1.24 | 1.22 | NA | NA | 1.13 | 1.14 | 1.13 | 1.13 |
| Short Sleep | ≤6 | 1.25 | 1.24 | 1.22 | 1.20 | 1.29 | 1.28 | 1.23 | 1.23 | 1.30 | 1.30 |
| Short Sleep | <7 | 1.13 | 1.13 | 1.16 | 1.15 | NA | NA | 1.30 | 1.30 | 1.25 | 1.26 |
| Long Sleep | >8 | 1.00 | 1.00 | 1.08 | 1.08 | NA | NA | 1.05 | 1.05 | 1.09 | 1.09 |
| Long Sleep | ≥9 | 1.09 | 1.10 | 1.11 | 1.11 | 1.18 | 1.16 | 1.05 | 1.05 | 1.19 | 1.20 |
| Long Sleep | >9 | 1.07 | 1.09 | 1.10 | 1.10 | NA | NA | 0.98 | 0.98 | 1.07 | 1.08 |
| Long Sleep | ≥10 | 0.85 | 0.90 | 1.08 | 1.08 | 1.31 | 1.28 | 1.09 | 1.09 | 0.94 | 0.95 |
| Insomnia WHIIRS | ≥9 | NA | NA | 1.15 | 1.14 | NA | NA | 1.13 | 1.12 | 1.30 | 1.29 |
| Insomnia WHIIRS | ≥10 | NA | NA | 1.15 | 1.15 | NA | NA | 1.17 | 1.17 | 1.29 | 1.29 |
| Insomnia WHIIRS 3 Ques | ≥5 | 1.17 | 1.17 | 1.18 | 1.17 | 1.17 | 1.08 | 1.09 | 1.09 | 1.29 | 1.28 |
| Insomnia WHIIRS 3 Ques | ≥6 | 1.15 | 1.15 | 1.16 | 1.15 | 1.22 | 1.12 | 1.11 | 1.10 | 1.26 | 1.25 |
| Insomnia Self-Report Dr Dx | Yes | NA | NA | 1.07 | 1.07 | 1.38 | 1.17 | 1.34 | 1.33 | 1.16 | 1.17 |
| Daytime Sleepiness ESS | ≥10 | 1.21 | 1.20 | 1.18 | 1.18 | 1.24 | 1.24 | 1.23 | 1.23 | 1.28 | 1.28 |
| Daytime Sleepiness ESS | ≥11 | 1.18 | 1.17 | 1.26 | 1.26 | 1.24 | 1.25 | 1.23 | 1.24 | 1.25 | 1.24 |
| Daytime Sleepiness ESS | ≥12 | 1.19 | 1.19 | 1.30 | 1.30 | 1.27 | 1.28 | 1.22 | 1.23 | 1.31 | 1.30 |
| Daytime Sleepiness Gen Ques | >1 | 0.97 | 0.97 | 1.07 | 1.07 | 1.12 | 1.12 | 1.01 | 1.00 | 1.18 | 1.18 |
| Daytime Sleepiness Gen Ques | >2 | 1.04 | 1.03 | 1.06 | 1.06 | 1.08 | 1.08 | 0.96 | 0.95 | 1.11 | 1.11 |
| Daytime Sleepiness Gen Ques | >4 | 1.27 | 1.26 | 1.12 | 1.12 | 1.05 | 1.04 | 0.96 | 0.95 | 1.27 | 1.26 |

OR = Odds ratio

3Q = 3 questions, Dr Dx = Self-reported doctor diagnosis, GenQ = General question

**Supplemental Table 5.** Associations of Million Veteran Program (MVP) Sleep PRSs Constructed across Ancestries and Summed with Sleep Phenotypes Definitions

for Short Sleep Long Sleep Insomnia and Daytime Sleepiness in HCHS/SOL (Visit 1) and MESA (Visit 5)

|  |  |  | **HCHS/SOL** | | |  | **MESA 5** | | |
| --- | --- | --- | --- | --- | --- | --- | --- | --- | --- |
| **Data** | **Tool** | **Phenotype** | **Estimate** | **SE** | **P-value** |  | **Estimate** | **SE** | **P-value** |
| MVP EUR + AFR | PRS-CSx | shrtslp_le5 | 0.15 | 0.08 | 0.05 |  | 0.08 | 0.19 | 0.68 |
| MVP EUR + AFR | PRS-CSx | shrtslp_lt6 | 0.12 | 0.05 | 0.02 |  | 0.03 | 0.12 | 0.77 |
| MVP EUR + AFR | PRS-CSx | shrtslp_le6 | 0.10 | 0.04 | 0.01 |  | 0.12 | 0.09 | 0.20 |
| MVP EUR + AFR | PRS-CSx | shrtslp_lt7 | 0.09 | 0.03 | 0.005 |  | 0.09 | 0.07 | 0.21 |
| MVP EUR + AFR | PRS-CSx | longslp_gt8 | 0.04 | 0.03 | 0.10 |  | 0.08 | 0.05 | 0.09 |
| MVP EUR + AFR | PRS-CSx | longslp_ge9 | 0.04 | 0.03 | 0.21 |  | 0.10 | 0.06 | 0.07 |
| MVP EUR + AFR | PRS-CSx | longslp_gt9 | 0.03 | 0.03 | 0.45 |  | 0.06 | 0.06 | 0.36 |
| MVP EUR + AFR | PRS-CSx | longslp_ge10 | 0.02 | 0.05 | 0.73 |  | 0.16 | 0.08 | 0.06 |
| MVP EUR + AFR + AMR + EAS | PRS-CSx | whiirs_ge9 | 0.08 | 0.03 | 0.01 |  | 0.05 | 0.05 | 0.35 |
| MVP EUR + AFR + AMR + EAS | PRS-CSx | whiirs_ge10 | 0.09 | 0.03 | 0.003 |  | 0.03 | 0.05 | 0.58 |
| MVP EUR + AFR + AMR + EAS | PRS-CSx | whiirs_3var_ge5 | 0.07 | 0.03 | 0.02 |  | 0.01 | 0.05 | 0.87 |
| MVP EUR + AFR + AMR + EAS | PRS-CSx | whiirs_3var_ge6 | 0.06 | 0.03 | 0.06 |  | 0.00 | 0.06 | 1.00 |
| MVP EUR + AFR + AMR + EAS | PRS-CSx | dr_dx | 0.16 | 0.04 | 0.0003 |  | 0.06 | 0.10 | 0.56 |
| MVP EUR + AFR + AMR + EAS | PRS-CSx | ess_10 | 0.02 | 0.05 | 0.67 |  | 0.21 | 0.12 | 0.07 |
| MVP EUR + AFR + AMR + EAS | PRS-CSx | ess_11 | -0.01 | 0.06 | 0.84 |  | 0.16 | 0.13 | 0.21 |
| MVP EUR + AFR + AMR + EAS | PRS-CSx | ess_12 | -0.01 | 0.06 | 0.84 |  | 0.27 | 0.14 | 0.07 |
| MVP EUR + AFR + AMR + EAS | PRS-CSx | slpyday_gt1 | 0.02 | 0.04 | 0.62 |  | 0.23 | 0.09 | 0.01 |
| MVP EUR + AFR + AMR + EAS | PRS-CSx | slpyday_gt2 | 0.06 | 0.04 | 0.13 |  | 0.25 | 0.10 | 0.009 |
| MVP EUR + AFR + AMR + EAS | PRS-CSx | slpyday_gt4 | 0.02 | 0.06 | 0.73 |  | 0.28 | 0.16 | 0.08 |

Beta and SE (standard error) for log odds ratio

EUR=European, AFR=African, AMR=Admixed American (primarily Hispanic), EAS=East Asian

WHIIRS=Women’s Health Initiative Insomnia Rating Scale, ESS=Epworth Sleepiness Scale

3var=3 WHIIRS questions, Dr Dx = Self-reported doctor diagnosis of insomnia, slpday = General question for excessive daytime sleepiness

**Supplemental Table 6.** Comparison of Associations between UKB and MVP Sleep PRSs and Sleep Phenotypes Definitions for Short Sleep and Long Sleep

|  |  |  | **HCHS/SOL** | | |  | **MESA 5** | | |  | **ARIC** | | |  | **BHS** | | |  | **MetaAnalysis** | | |  |
| --- | --- | --- | --- | --- | --- | --- | --- | --- | --- | --- | --- | --- | --- | --- | --- | --- | --- | --- | --- | --- | --- | --- |
| **Data** | **Tool** | **Phenotype** | **Estimate** | **SE** | **P-value** |  | **Estimate** | **SE** | **P-value** |  | **Estimate** | **SE** | **P-value** |  | **Estimate** | **SE** | **P-value** |  | **Estimate** | **SE** | **P-value** | **Phet** |
| UKB | PRS-CS | shrtslp_le5 | 0.34 | 0.09 | 1.44E-04 |  | 0.20 | 0.28 | 4.59E-01 |  | 0.16 | 0.19 | 3.86E-01 |  | 0.14 | 0.17 | 4.31E-01 |  | 0.27 | 0.07 | 1.11E-04 | 0.66 |
| MVP EUR | PRS-CS | shrtslp_le5 | 0.16 | 0.10 | 1.03E-01 |  | -0.11 | 0.33 | 7.43E-01 |  | 0.05 | 0.19 | 7.77E-01 |  | 0.58 | 0.26 | 2.49E-02 |  | 0.17 | 0.08 | 3.90E-02 | 0.31 |
| MVP EUR | PRS-CSx | shrtslp_le5 | 0.25 | 0.10 | 1.37E-02 |  | 0.05 | 0.35 | 8.92E-01 |  | -0.02 | 0.19 | 9.16E-01 |  | 0.65 | 0.24 | 6.57E-03 |  | 0.23 | 0.08 | 3.72E-03 | 0.16 |
| UKB+MVP EUR (meta) | PRS-CS | shrtslp_le5 | 0.30 | 0.08 | 3.27E-04 |  | -0.02 | 0.23 | 9.24E-01 |  | -0.13 | 0.19 | 4.88E-01 |  | 0.15 | 0.16 | 3.46E-01 |  | 0.19 | 0.06 | 2.86E-03 | 0.14 |
| UKB + MVP EUR (sum) | PRS-CS | shrtslp_le5 | 0.34 | 0.10 | 9.14E-04 |  | 0.10 | 0.34 | 7.60E-01 |  | 0.14 | 0.19 | 4.50E-01 |  | 0.36 | 0.23 | 1.13E-01 |  | 0.29 | 0.08 | 3.08E-04 | 0.74 |
| UKB + MVP EUR (sum) | PRS-CS PRS-CSx | shrtslp_le5 | 0.36 | 0.10 | 4.18E-04 |  | 0.20 | 0.35 | 5.73E-01 |  | 0.10 | 0.19 | 6.09E-01 |  | 0.41 | 0.22 | 6.05E-02 |  | 0.31 | 0.08 | 1.24E-04 | 0.58 |
| UKB | PRS-CS | shrtslp_lt6 | 0.21 | 0.06 | 7.47E-04 |  | 0.13 | 0.17 | 4.64E-01 |  | 0.22 | 0.12 | 5.32E-02 |  | 0.12 | 0.14 | 3.94E-01 |  | 0.20 | 0.05 | 6.53E-05 | 0.90 |
| MVP EUR | PRS-CS | shrtslp_lt6 | 0.19 | 0.07 | 3.84E-03 |  | -0.04 | 0.21 | 8.58E-01 |  | -0.02 | 0.12 | 8.69E-01 |  | 0.45 | 0.21 | 3.53E-02 |  | 0.15 | 0.05 | 5.60E-03 | 0.16 |
| MVP EUR | PRS-CSx | shrtslp_lt6 | 0.24 | 0.07 | 4.64E-04 |  | -0.06 | 0.22 | 8.00E-01 |  | -0.12 | 0.12 | 2.84E-01 |  | 0.45 | 0.19 | 1.80E-02 |  | 0.16 | 0.05 | 3.36E-03 | 0.01 |
| UKB+MVP EUR (meta) | PRS-CS | shrtslp_lt6 | 0.16 | 0.06 | 8.88E-03 |  | 0.03 | 0.14 | 8.41E-01 |  | -0.01 | 0.12 | 9.64E-01 |  | 0.08 | 0.13 | 5.25E-01 |  | 0.11 | 0.05 | 2.12E-02 | 0.57 |
| UKB + MVP EUR (sum) | PRS-CS | shrtslp_lt6 | 0.26 | 0.07 | 1.74E-04 |  | 0.08 | 0.21 | 7.02E-01 |  | 0.14 | 0.12 | 2.41E-01 |  | 0.29 | 0.19 | 1.19E-01 |  | 0.22 | 0.05 | 4.08E-05 | 0.71 |
| UKB + MVP EUR (sum) | PRS-CS PRS-CSx | shrtslp_lt6 | 0.27 | 0.07 | 4.81E-05 |  | 0.08 | 0.22 | 7.24E-01 |  | 0.07 | 0.12 | 5.51E-01 |  | 0.31 | 0.18 | 8.17E-02 |  | 0.22 | 0.05 | 3.35E-05 | 0.38 |
| UKB | PRS-CS | shrtslp_le6 | 0.20 | 0.05 | 1.21E-04 |  | 0.21 | 0.14 | 1.32E-01 |  | 0.22 | 0.10 | 2.33E-02 |  | 0.26 | 0.11 | 2.27E-02 |  | 0.21 | 0.04 | 1.80E-07 | 0.97 |
| MVP EUR | PRS-CS | shrtslp_le6 | 0.16 | 0.06 | 5.61E-03 |  | 0.15 | 0.16 | 3.54E-01 |  | 0.10 | 0.10 | 3.30E-01 |  | 0.64 | 0.17 | 1.66E-04 |  | 0.18 | 0.04 | 8.37E-05 | 0.04 |
| MVP EUR | PRS-CSx | shrtslp_le6 | 0.19 | 0.05 | 4.43E-04 |  | 0.23 | 0.17 | 1.73E-01 |  | 0.09 | 0.10 | 3.89E-01 |  | 0.61 | 0.15 | 8.04E-05 |  | 0.21 | 0.04 | 2.36E-06 | 0.04 |
| UKB+MVP EUR (meta) | PRS-CS | shrtslp_le6 | 0.15 | 0.05 | 3.51E-03 |  | 0.10 | 0.11 | 3.94E-01 |  | 0.01 | 0.10 | 8.93E-01 |  | 0.23 | 0.10 | 2.61E-02 |  | 0.13 | 0.04 | 6.05E-04 | 0.47 |
| UKB + MVP EUR (sum) | PRS-CS | shrtslp_le6 | 0.23 | 0.06 | 4.62E-05 |  | 0.25 | 0.17 | 1.37E-01 |  | 0.21 | 0.10 | 3.32E-02 |  | 0.49 | 0.15 | 9.69E-04 |  | 0.25 | 0.04 | 1.99E-08 | 0.39 |
| UKB + MVP EUR (sum) | PRS-CS PRS-CSx | shrtslp_le6 | 0.24 | 0.05 | 9.75E-06 |  | 0.29 | 0.17 | 8.67E-02 |  | 0.20 | 0.10 | 3.75E-02 |  | 0.50 | 0.14 | 5.44E-04 |  | 0.26 | 0.04 | 2.14E-09 | 0.35 |
| UKB | PRS-CS | shrtslp_lt7 | 0.15 | 0.04 | 2.58E-04 |  | 0.26 | 0.10 | 5.57E-03 |  | 0.13 | 0.06 | 3.57E-02 |  | 0.22 | 0.10 | 3.19E-02 |  | 0.16 | 0.03 | 1.13E-07 | 0.57 |
| MVP EUR | PRS-CS | shrtslp_lt7 | 0.15 | 0.04 | 3.88E-04 |  | 0.16 | 0.11 | 1.67E-01 |  | 0.12 | 0.06 | 5.08E-02 |  | 0.47 | 0.15 | 1.67E-03 |  | 0.16 | 0.03 | 1.30E-06 | 0.18 |
| MVP EUR | PRS-CSx | shrtslp_lt7 | 0.11 | 0.04 | 7.74E-03 |  | 0.23 | 0.12 | 5.28E-02 |  | 0.05 | 0.06 | 4.27E-01 |  | 0.46 | 0.14 | 7.20E-04 |  | 0.12 | 0.03 | 1.54E-04 | 0.03 |
| UKB+MVP EUR (meta) | PRS-CS | shrtslp_lt7 | 0.12 | 0.04 | 2.10E-03 |  | 0.18 | 0.08 | 2.08E-02 |  | 0.09 | 0.06 | 1.38E-01 |  | 0.19 | 0.09 | 4.42E-02 |  | 0.13 | 0.03 | 8.93E-06 | 0.71 |
| UKB + MVP EUR (sum) | PRS-CS | shrtslp_lt7 | 0.19 | 0.04 | 1.58E-05 |  | 0.30 | 0.12 | 1.08E-02 |  | 0.16 | 0.06 | 8.26E-03 |  | 0.39 | 0.13 | 3.52E-03 |  | 0.20 | 0.03 | 8.45E-10 | 0.34 |
| UKB + MVP EUR (sum) | PRS-CS PRS-CSx | shrtslp_lt7 | 0.16 | 0.04 | 1.42E-04 |  | 0.34 | 0.12 | 4.54E-03 |  | 0.12 | 0.06 | 5.41E-02 |  | 0.40 | 0.13 | 1.99E-03 |  | 0.17 | 0.03 | 4.57E-08 | 0.11 |
| UKB | PRS-CS | longslp_gt8 | 0.08 | 0.03 | 3.60E-03 |  | 0.05 | 0.04 | 2.77E-01 |  | 0.00 | 0.06 | 9.56E-01 |  | 0.08 | 0.10 | 4.05E-01 |  | 0.06 | 0.02 | 2.82E-03 | 0.68 |
| MVP EUR | PRS-CS | longslp_gt8 | 0.02 | 0.03 | 3.87E-01 |  | 0.09 | 0.05 | 6.68E-02 |  | -0.05 | 0.06 | 4.74E-01 |  | 0.13 | 0.11 | 2.09E-01 |  | 0.03 | 0.02 | 1.22E-01 | 0.28 |
| MVP EUR | PRS-CSx | longslp_gt8 | 0.06 | 0.03 | 2.55E-02 |  | 0.12 | 0.05 | 2.83E-02 |  | -0.04 | 0.06 | 5.45E-01 |  | 0.14 | 0.11 | 2.15E-01 |  | 0.06 | 0.02 | 5.49E-03 | 0.26 |
| UKB+MVP EUR (meta) | PRS-CS | longslp_gt8 | 0.07 | 0.03 | 8.05E-03 |  | 0.08 | 0.04 | 7.20E-02 |  | 0.04 | 0.06 | 5.56E-01 |  | 0.11 | 0.09 | 2.36E-01 |  | 0.07 | 0.02 | 6.91E-04 | 0.91 |
| UKB + MVP EUR (sum) | PRS-CS | longslp_gt8 | 0.06 | 0.03 | 2.19E-02 |  | 0.08 | 0.05 | 7.77E-02 |  | -0.03 | 0.06 | 6.08E-01 |  | 0.14 | 0.11 | 2.01E-01 |  | 0.06 | 0.02 | 6.33E-03 | 0.42 |
| UKB + MVP EUR (sum) | PRS-CS PRS-CSx | longslp_gt8 | 0.08 | 0.03 | 1.89E-03 |  | 0.09 | 0.05 | 5.49E-02 |  | -0.03 | 0.06 | 6.64E-01 |  | 0.14 | 0.11 | 2.07E-01 |  | 0.07 | 0.02 | 5.31E-04 | 0.36 |
| UKB | PRS-CS | longslp_ge9 | 0.10 | 0.03 | 1.94E-03 |  | 0.05 | 0.05 | 3.50E-01 |  | 0.08 | 0.11 | 4.48E-01 |  | 0.18 | 0.14 | 2.16E-01 |  | 0.09 | 0.03 | 6.76E-04 | 0.77 |
| MVP EUR | PRS-CS | longslp_ge9 | 0.04 | 0.03 | 1.82E-01 |  | 0.11 | 0.06 | 5.80E-02 |  | -0.01 | 0.11 | 9.05E-01 |  | 0.18 | 0.15 | 2.31E-01 |  | 0.06 | 0.03 | 2.91E-02 | 0.56 |
| MVP EUR | PRS-CSx | longslp_ge9 | 0.06 | 0.03 | 4.86E-02 |  | 0.16 | 0.06 | 8.55E-03 |  | 0.08 | 0.11 | 4.44E-01 |  | 0.09 | 0.16 | 5.56E-01 |  | 0.08 | 0.03 | 2.05E-03 | 0.52 |
| UKB+MVP EUR (meta) | PRS-CS | longslp_ge9 | 0.09 | 0.03 | 6.59E-03 |  | 0.08 | 0.05 | 1.35E-01 |  | 0.12 | 0.11 | 2.69E-01 |  | 0.16 | 0.14 | 2.27E-01 |  | 0.09 | 0.03 | 5.46E-04 | 0.94 |
| UKB + MVP EUR (sum) | PRS-CS | longslp_ge9 | 0.09 | 0.03 | 4.38E-03 |  | 0.09 | 0.05 | 8.81E-02 |  | 0.05 | 0.11 | 6.53E-01 |  | 0.23 | 0.15 | 1.30E-01 |  | 0.09 | 0.03 | 3.92E-04 | 0.80 |
| UKB + MVP EUR (sum) | PRS-CS PRS-CSx | longslp_ge9 | 0.10 | 0.03 | 1.48E-03 |  | 0.11 | 0.05 | 3.91E-02 |  | 0.11 | 0.11 | 3.10E-01 |  | 0.18 | 0.16 | 2.49E-01 |  | 0.10 | 0.03 | 4.91E-05 | 0.95 |
| UKB | PRS-CS | longslp_gt9 | 0.09 | 0.04 | 8.69E-03 |  | -0.02 | 0.06 | 7.25E-01 |  | 0.07 | 0.14 | 6.11E-01 |  | 0.07 | 0.17 | 6.67E-01 |  | 0.06 | 0.03 | 3.05E-02 | 0.42 |
| MVP EUR | PRS-CS | longslp_gt9 | 0.05 | 0.04 | 1.38E-01 |  | 0.08 | 0.06 | 2.12E-01 |  | 0.01 | 0.14 | 9.69E-01 |  | 0.12 | 0.17 | 4.84E-01 |  | 0.06 | 0.03 | 4.93E-02 | 0.94 |
| MVP EUR | PRS-CSx | longslp_gt9 | 0.06 | 0.03 | 9.26E-02 |  | 0.13 | 0.07 | 6.64E-02 |  | 0.20 | 0.14 | 1.64E-01 |  | 0.09 | 0.18 | 6.16E-01 |  | 0.08 | 0.03 | 8.95E-03 | 0.67 |
| UKB+MVP EUR (meta) | PRS-CS | longslp_gt9 | 0.09 | 0.04 | 1.00E-02 |  | 0.01 | 0.06 | 8.55E-01 |  | 0.22 | 0.14 | 1.20E-01 |  | 0.07 | 0.16 | 6.63E-01 |  | 0.08 | 0.03 | 9.26E-03 | 0.46 |
| UKB + MVP EUR (sum) | PRS-CS | longslp_gt9 | 0.09 | 0.03 | 8.63E-03 |  | 0.03 | 0.06 | 6.11E-01 |  | 0.05 | 0.14 | 7.03E-01 |  | 0.13 | 0.18 | 4.84E-01 |  | 0.08 | 0.03 | 8.43E-03 | 0.83 |
| UKB + MVP EUR (sum) | PRS-CS PRS-CSx | longslp_gt9 | 0.09 | 0.03 | 7.33E-03 |  | 0.05 | 0.06 | 4.34E-01 |  | 0.18 | 0.14 | 2.09E-01 |  | 0.11 | 0.19 | 5.65E-01 |  | 0.09 | 0.03 | 2.87E-03 | 0.83 |
| UKB | PRS-CS | shrtslp_ge10 | 0.08 | 0.05 | 1.11E-01 |  | 0.09 | 0.08 | 2.59E-01 |  | -0.16 | 0.29 | 5.76E-01 |  | -0.06 | 0.22 | 7.80E-01 |  | 0.07 | 0.04 | 7.76E-02 | 0.78 |
| MVP EUR | PRS-CS | shrtslp_ge10 | 0.05 | 0.05 | 3.19E-01 |  | 0.18 | 0.09 | 3.65E-02 |  | 0.17 | 0.29 | 5.47E-01 |  | 0.02 | 0.23 | 9.37E-01 |  | 0.08 | 0.04 | 5.43E-02 | 0.57 |
| MVP EUR | PRS-CSx | shrtslp_ge10 | 0.04 | 0.05 | 4.20E-01 |  | 0.24 | 0.09 | 1.16E-02 |  | 0.28 | 0.30 | 3.48E-01 |  | 0.06 | 0.24 | 7.92E-01 |  | 0.08 | 0.04 | 5.14E-02 | 0.24 |
| UKB+MVP EUR (meta) | PRS-CS | shrtslp_ge10 | 0.09 | 0.05 | 8.03E-02 |  | 0.10 | 0.08 | 1.96E-01 |  | 0.02 | 0.29 | 9.46E-01 |  | -0.03 | 0.21 | 8.87E-01 |  | 0.09 | 0.04 | 3.72E-02 | 0.94 |
| UKB + MVP EUR (sum) | PRS-CS | shrtslp_ge10 | 0.08 | 0.04 | 6.57E-02 |  | 0.16 | 0.08 | 5.16E-02 |  | 0.00 | 0.30 | 9.98E-01 |  | -0.03 | 0.24 | 8.99E-01 |  | 0.09 | 0.04 | 1.37E-02 | 0.77 |
| UKB + MVP EUR (sum) | PRS-CS PRS-CSx | shrtslp_ge10 | 0.07 | 0.04 | 9.42E-02 |  | 0.18 | 0.08 | 3.28E-02 |  | 0.06 | 0.29 | 8.27E-01 |  | -0.01 | 0.24 | 9.80E-01 |  | 0.09 | 0.04 | 1.48E-02 | 0.69 |
| UKB | PRS-CS | sleep duration | 0.11 | 0.02 | 5.45E-08 |  | 0.14 | 0.04 | 6.13E-05 |  | 0.09 | 0.03 | 3.23E-04 |  | 0.11 | 0.05 | 4.41E-02 |  | 0.11 | 0.01 | 2.60E-15 | 0.69 |

Beta and SE (standard error) for log odds ratio

**Supplemental Table 7.** Comparison of Associations between UKB and MVP Sleep PRSs and Sleep Phenotypes Definitions for Insomnia

|  |  |  | **HCHS/SOL** | | |  | **MESA 5** | | |  | **ARIC** | | |  | **BHS** | | |  | **MetaAnalysis** | | |  |
| --- | --- | --- | --- | --- | --- | --- | --- | --- | --- | --- | --- | --- | --- | --- | --- | --- | --- | --- | --- | --- | --- | --- |
| **Data** | **Tool** | **Phenotype** | **Estimate** | **SE** | **P-value** |  | **Estimate** | **SE** | **P-value** |  | **Estimate** | **SE** | **P-value** |  | **Estimate** | **SE** | **P-value** |  | **Estimate** | **SE** | **P-value** | **Phet** |
| UKB | PRS-CS | whiirs_ge9 | 0.14 | 0.03 | 1.42E-05 |  | 0.12 | 0.07 | 6.52E-02 |  | NA | NA | NA |  | 0.26 | 0.09 | 2.82E-03 |  | 0.15 | 0.03 | 5.72E-08 | 0.37 |
| MVP EUR | PRS-CS | whiirs_ge9 | 0.19 | 0.07 | 5.04E-03 |  | 0.35 | 0.20 | 7.75E-02 |  | NA | NA | NA |  | 0.22 | 0.28 | 4.28E-01 |  | 0.21 | 0.06 | 8.80E-04 | 0.74 |
| MVP EUR | PRS-CSx | whiirs_ge9 | 0.18 | 0.05 | 6.95E-04 |  | 0.27 | 0.16 | 9.32E-02 |  | NA | NA | NA |  | 0.08 | 0.23 | 7.26E-01 |  | 0.19 | 0.05 | 1.80E-04 | 0.77 |
| UKB+MVP EUR (meta) | PRS-CS | whiirs_ge9 | 0.26 | 0.05 | 1.98E-06 |  | 0.36 | 0.15 | 2.09E-02 |  | NA | NA | NA |  | 0.62 | 0.21 | 3.73E-03 |  | 0.29 | 0.05 | 5.69E-09 | 0.24 |
| UKB + MVP EUR (sum) | PRS-CS | whiirs_ge9 | 0.09 | 0.04 | 2.40E-02 |  | 0.24 | 0.11 | 2.81E-02 |  | NA | NA | NA |  | 0.44 | 0.15 | 3.02E-03 |  | 0.13 | 0.04 | 4.25E-04 | 0.04 |
| UKB + MVP EUR (sum) | PRS-CS PRS-CSx | whiirs_ge9 | 0.21 | 0.04 | 7.77E-07 |  | 0.23 | 0.10 | 2.74E-02 |  | NA | NA | NA |  | 0.39 | 0.14 | 6.11E-03 |  | 0.23 | 0.04 | 2.08E-09 | 0.50 |
| UKB | PRS-CS | whiirs_ge10 | 0.14 | 0.03 | 1.23E-05 |  | 0.16 | 0.07 | 2.32E-02 |  | NA | NA | NA |  | 0.26 | 0.09 | 4.18E-03 |  | 0.16 | 0.03 | 2.13E-08 | 0.49 |
| MVP EUR | PRS-CS | whiirs_ge10 | 0.25 | 0.06 | 3.12E-05 |  | 0.14 | 0.21 | 5.10E-01 |  | NA | NA | NA |  | 0.41 | 0.29 | 1.53E-01 |  | 0.25 | 0.06 | 1.03E-05 | 0.74 |
| MVP EUR | PRS-CSx | whiirs_ge10 | 0.23 | 0.05 | 2.88E-05 |  | 0.15 | 0.17 | 3.72E-01 |  | NA | NA | NA |  | 0.26 | 0.23 | 2.56E-01 |  | 0.22 | 0.05 | 9.32E-06 | 0.91 |
| UKB+MVP EUR (meta) | PRS-CS | whiirs_ge10 | 0.32 | 0.05 | 1.49E-09 |  | 0.39 | 0.16 | 1.71E-02 |  | NA | NA | NA |  | 0.67 | 0.22 | 2.47E-03 |  | 0.34 | 0.05 | 1.54E-12 | 0.29 |
| UKB + MVP EUR (sum) | PRS-CS | whiirs_ge10 | 0.10 | 0.04 | 1.36E-02 |  | 0.25 | 0.11 | 2.42E-02 |  | NA | NA | NA |  | 0.46 | 0.15 | 2.45E-03 |  | 0.14 | 0.04 | 1.75E-04 | 0.04 |
| UKB + MVP EUR (sum) | PRS-CS PRS-CSx | whiirs_ge10 | 0.24 | 0.04 | 5.27E-08 |  | 0.25 | 0.11 | 2.12E-02 |  | NA | NA | NA |  | 0.42 | 0.15 | 3.79E-03 |  | 0.25 | 0.04 | 6.76E-11 | 0.48 |
| UKB | PRS-CS | whiirs_3var_ge5 | 0.16 | 0.03 | 1.21E-07 |  | 0.09 | 0.07 | 1.99E-01 |  | 0.16 | 0.06 | 3.22E-03 |  | 0.25 | 0.09 | 3.91E-03 |  | 0.16 | 0.02 | 1.89E-11 | 0.55 |
| MVP EUR | PRS-CS | whiirs_3var_ge5 | 0.20 | 0.07 | 3.02E-03 |  | 0.19 | 0.21 | 3.82E-01 |  | 0.13 | 0.06 | 2.34E-02 |  | 0.06 | 0.28 | 8.25E-01 |  | 0.16 | 0.04 | 1.79E-04 | 0.86 |
| MVP EUR | PRS-CSx | whiirs_3var_ge5 | 0.19 | 0.05 | 5.18E-04 |  | 0.15 | 0.18 | 3.89E-01 |  | 0.10 | 0.06 | 7.45E-02 |  | -0.04 | 0.22 | 8.60E-01 |  | 0.14 | 0.04 | 1.66E-04 | 0.57 |
| UKB+MVP EUR (meta) | PRS-CS | whiirs_3var_ge5 | 0.27 | 0.05 | 5.85E-07 |  | 0.23 | 0.17 | 1.73E-01 |  | 0.17 | 0.06 | 2.07E-03 |  | 0.51 | 0.21 | 1.57E-02 |  | 0.23 | 0.04 | 3.88E-10 | 0.31 |
| UKB + MVP EUR (sum) | PRS-CS | whiirs_3var_ge5 | 0.11 | 0.04 | 4.93E-03 |  | 0.16 | 0.12 | 1.54E-01 |  | 0.21 | 0.06 | 3.10E-04 |  | 0.40 | 0.15 | 6.67E-03 |  | 0.15 | 0.03 | 4.31E-07 | 0.19 |
| UKB + MVP EUR (sum) | PRS-CS PRS-CSx | whiirs_3var_ge5 | 0.25 | 0.04 | 1.07E-08 |  | 0.16 | 0.11 | 1.53E-01 |  | 0.18 | 0.06 | 1.07E-03 |  | 0.35 | 0.14 | 1.32E-02 |  | 0.22 | 0.03 | 1.25E-12 | 0.59 |
| UKB | PRS-CS | whiirs_3var_ge6 | 0.15 | 0.03 | 6.05E-06 |  | 0.10 | 0.07 | 1.69E-01 |  | 0.14 | 0.05 | 7.12E-03 |  | 0.23 | 0.09 | 8.90E-03 |  | 0.15 | 0.02 | 2.60E-09 | 0.73 |
| MVP EUR | PRS-CS | whiirs_3var_ge6 | 0.22 | 0.07 | 1.78E-03 |  | 0.31 | 0.22 | 1.68E-01 |  | 0.13 | 0.06 | 2.21E-02 |  | 0.10 | 0.28 | 7.12E-01 |  | 0.17 | 0.04 | 8.19E-05 | 0.66 |
| MVP EUR | PRS-CSx | whiirs_3var_ge6 | 0.21 | 0.06 | 3.74E-04 |  | 0.24 | 0.18 | 1.96E-01 |  | 0.11 | 0.05 | 3.65E-02 |  | -0.02 | 0.23 | 9.20E-01 |  | 0.15 | 0.04 | 4.72E-05 | 0.53 |
| UKB+MVP EUR (meta) | PRS-CS | whiirs_3var_ge6 | 0.28 | 0.06 | 1.11E-06 |  | 0.29 | 0.17 | 9.79E-02 |  | 0.11 | 0.05 | 3.59E-02 |  | 0.50 | 0.21 | 1.87E-02 |  | 0.20 | 0.04 | 5.13E-08 | 0.07 |
| UKB + MVP EUR (sum) | PRS-CS | whiirs_3var_ge6 | 0.12 | 0.04 | 3.15E-03 |  | 0.20 | 0.12 | 9.73E-02 |  | 0.19 | 0.06 | 5.70E-04 |  | 0.37 | 0.15 | 1.22E-02 |  | 0.16 | 0.03 | 2.69E-07 | 0.37 |
| UKB + MVP EUR (sum) | PRS-CS PRS-CSx | whiirs_3var_ge6 | 0.24 | 0.05 | 2.86E-07 |  | 0.20 | 0.12 | 9.65E-02 |  | 0.18 | 0.05 | 9.47E-04 |  | 0.32 | 0.14 | 2.35E-02 |  | 0.22 | 0.03 | 2.65E-11 | 0.74 |
| UKB | PRS-CS | dr_dx | 0.07 | 0.05 | 1.55E-01 |  | 0.29 | 0.13 | 2.34E-02 |  | NA | NA | NA |  | 0.15 | 0.16 | 3.32E-01 |  | 0.10 | 0.04 | 1.93E-02 | 0.27 |
| MVP EUR | PRS-CS | dr_dx | 0.40 | 0.09 | 6.46E-06 |  | 0.91 | 0.40 | 2.21E-02 |  | NA | NA | NA |  | 0.58 | 0.51 | 2.53E-01 |  | 0.43 | 0.09 | 4.21E-07 | 0.44 |
| MVP EUR | PRS-CSx | dr_dx | 0.33 | 0.08 | 2.44E-05 |  | 0.97 | 0.33 | 2.84E-03 |  | NA | NA | NA |  | 0.45 | 0.41 | 2.68E-01 |  | 0.37 | 0.07 | 7.39E-07 | 0.16 |
| UKB+MVP EUR (meta) | PRS-CS | dr_dx | 0.34 | 0.08 | 3.56E-05 |  | 0.82 | 0.30 | 6.81E-03 |  | NA | NA | NA |  | 0.65 | 0.38 | 9.15E-02 |  | 0.38 | 0.08 | 6.83E-07 | 0.24 |
| UKB + MVP EUR (sum) | PRS-CS | dr_dx | 0.02 | 0.06 | 7.81E-01 |  | 0.58 | 0.21 | 6.07E-03 |  | NA | NA | NA |  | 0.32 | 0.27 | 2.25E-01 |  | 0.07 | 0.05 | 2.27E-01 | 0.02 |
| UKB + MVP EUR (sum) | PRS-CS PRS-CSx | dr_dx | 0.20 | 0.07 | 4.61E-03 |  | 0.63 | 0.21 | 2.40E-03 |  | NA | NA | NA |  | 0.31 | 0.26 | 2.19E-01 |  | 0.25 | 0.06 | 1.13E-04 | 0.14 |
| UKB | PRS-CS | whiirs | 0.38 | 0.07 | 5.61E-08 |  | 0.33 | 0.14 | 1.64E-02 |  | NA | NA | NA |  | 0.71 | 0.22 | 1.57E-03 |  | 0.40 | 0.06 | 3.70E-11 | 0.32 |
| MVP EUR | PRS-CS | whiirs | 0.66 | 0.14 | 2.64E-06 |  | 0.36 | 0.41 | 3.80E-01 |  | NA | NA | NA |  | 0.51 | 0.74 | 4.84E-01 |  | 0.63 | 0.13 | 1.43E-06 | 0.78 |
| MVP EUR | PRS-CSx | whiirs | 0.63 | 0.12 | 3.13E-07 |  | 0.30 | 0.34 | 3.85E-01 |  | NA | NA | NA |  | 0.15 | 0.59 | 7.95E-01 |  | 0.57 | 0.11 | 3.10E-07 | 0.50 |
| UKB+MVP EUR (meta) | PRS-CS | whiirs | 0.79 | 0.12 | 2.56E-11 |  | 0.77 | 0.32 | 1.58E-02 |  | NA | NA | NA |  | 1.61 | 0.55 | 3.33E-03 |  | 0.82 | 0.11 | 2.09E-14 | 0.34 |
| UKB + MVP EUR (sum) | PRS-CS | whiirs | 0.64 | 0.10 | 3.49E-10 |  | 0.55 | 0.22 | 1.48E-02 |  | NA | NA | NA |  | 1.18 | 0.38 | 1.94E-03 |  | 0.66 | 0.09 | 1.89E-13 | 0.34 |
| UKB + MVP EUR (sum) | PRS-CS PRS-CSx | whiirs | 0.65 | 0.10 | 7.10E-11 |  | 0.52 | 0.22 | 1.59E-02 |  | NA | NA | NA |  | 1.04 | 0.36 | 4.21E-03 |  | 0.65 | 0.09 | 5.55E-14 | 0.47 |
| UKB | PRS-CS | whiirs_3var | 0.24 | 0.05 | 8.67E-07 |  | 0.10 | 0.10 | 3.36E-01 |  | 0.20 | 0.06 | 1.71E-03 |  | 0.46 | 0.15 | 1.74E-03 |  | 0.22 | 0.03 | 1.75E-10 | 0.20 |
| MVP EUR | PRS-CS | whiirs_3var | 0.41 | 0.10 | 2.74E-05 |  | 0.21 | 0.30 | 4.88E-01 |  | 0.17 | 0.07 | 7.11E-03 |  | 0.20 | 0.48 | 6.74E-01 |  | 0.25 | 0.05 | 3.56E-06 | 0.24 |
| MVP EUR | PRS-CSx | whiirs_3var | 0.37 | 0.09 | 1.99E-05 |  | 0.21 | 0.25 | 4.03E-01 |  | 0.12 | 0.06 | 6.72E-02 |  | 0.03 | 0.38 | 9.43E-01 |  | 0.20 | 0.05 | 4.20E-05 | 0.12 |
| UKB+MVP EUR (meta) | PRS-CS | whiirs_3var | 0.48 | 0.08 | 1.86E-08 |  | 0.32 | 0.23 | 1.67E-01 |  | 0.19 | 0.06 | 2.89E-03 |  | 1.01 | 0.36 | 4.98E-03 |  | 0.31 | 0.05 | 3.15E-10 | 0.009 |
| UKB + MVP EUR (sum) | PRS-CS | whiirs_3var | 0.41 | 0.07 | 1.22E-08 |  | 0.18 | 0.16 | 2.81E-01 |  | 0.26 | 0.06 | 5.16E-05 |  | 0.74 | 0.25 | 2.78E-03 |  | 0.33 | 0.04 | 2.22E-13 | 0.11 |
| UKB + MVP EUR (sum) | PRS-CS PRS-CSx | whiirs_3var | 0.40 | 0.07 | 8.33E-09 |  | 0.18 | 0.16 | 2.56E-01 |  | 0.22 | 0.06 | 5.66E-04 |  | 0.66 | 0.24 | 5.60E-03 |  | 0.30 | 0.04 | 3.60E-12 | 0.08 |
| UKB | PRS-CS | whiirs_ge9_3var_ge5 | NA | NA | NA |  | NA | NA | NA |  | NA | NA | NA |  | NA | NA | NA |  | 0.15 | 0.02 | 7.23E-10 | 0.56 |
| MVP EUR | PRS-CS | whiirs_ge9_3var_ge5 | NA | NA | NA |  | NA | NA | NA |  | NA | NA | NA |  | NA | NA | NA |  | 0.16 | 0.04 | 8.99E-05 | 0.69 |
| MVP EUR | PRS-CSx | whiirs_ge9_3var_ge5 | NA | NA | NA |  | NA | NA | NA |  | NA | NA | NA |  | NA | NA | NA |  | 0.15 | 0.04 | 6.65E-05 | 0.60 |
| UKB+MVP EUR (meta) | PRS-CS | whiirs_ge9_3var_ge5 | NA | NA | NA |  | NA | NA | NA |  | NA | NA | NA |  | NA | NA | NA |  | 0.24 | 0.04 | 1.66E-10 | 0.14 |
| UKB + MVP EUR (sum) | PRS-CS | whiirs_ge9_3var_ge5 | NA | NA | NA |  | NA | NA | NA |  | NA | NA | NA |  | NA | NA | NA |  | 0.15 | 0.03 | 9.32E-07 | 0.05 |
| UKB + MVP EUR (sum) | PRS-CS PRS-CSx | whiirs_ge9_3var_ge5 | NA | NA | NA |  | NA | NA | NA |  | NA | NA | NA |  | NA | NA | NA |  | 0.21 | 0.03 | 1.19E-11 | 0.60 |
| UKB | PRS-CS | whiirs_ge10_3var_ge6 | NA | NA | NA |  | NA | NA | NA |  | NA | NA | NA |  | NA | NA | NA |  | 0.15 | 0.02 | 5.51E-10 | 0.69 |
| MVP EUR | PRS-CS | whiirs_ge10_3var_ge6 | NA | NA | NA |  | NA | NA | NA |  | NA | NA | NA |  | NA | NA | NA |  | 0.19 | 0.04 | 2.41E-06 | 0.38 |
| MVP EUR | PRS-CSx | whiirs_ge10_3var_ge6 | NA | NA | NA |  | NA | NA | NA |  | NA | NA | NA |  | NA | NA | NA |  | 0.17 | 0.04 | 3.11E-06 | 0.49 |
| UKB+MVP EUR (meta) | PRS-CS | whiirs_ge10_3var_ge6 | NA | NA | NA |  | NA | NA | NA |  | NA | NA | NA |  | NA | NA | NA |  | 0.24 | 0.04 | 2.85E-11 | 0.006 |
| UKB + MVP EUR (sum) | PRS-CS | whiirs_ge10_3var_ge6 | NA | NA | NA |  | NA | NA | NA |  | NA | NA | NA |  | NA | NA | NA |  | 0.16 | 0.03 | 4.69E-07 | 0.08 |
| UKB + MVP EUR (sum) | PRS-CS PRS-CSx | whiirs_ge10_3var_ge6 | NA | NA | NA |  | NA | NA | NA |  | NA | NA | NA |  | NA | NA | NA |  | 0.23 | 0.03 | 5.08E-13 | 0.43 |

Beta and SE (standard error) for log odds ratio; WHIIRS=Women’s Health Study Initiative Insomnia Rating Scale, Dr Dx = Self-reported doctor diagnosis of insomnia , 3var= 3WHIIRS questions

**Supplemental Table 8.** Comparison of Associations between UKB and MVP Sleep PRSs and Sleep Phenotypes Definitions for Excessive Daytime Sleepiness

|  |  |  | **HCHS/SOL** | | |  | **MESA 5** | | |  | **ARIC** | | |  | **BHS** | | |  | **MetaAnalysis** | | |  |
| --- | --- | --- | --- | --- | --- | --- | --- | --- | --- | --- | --- | --- | --- | --- | --- | --- | --- | --- | --- | --- | --- | --- |
| **Data** | **Tool** | **Phenotype** | **Estimate** | **SE** | **P-value** |  | **Estimate** | **SE** | **P-value** |  | **Estimate** | **SE** | **P-value** |  | **Estimate** | **SE** | **P-value** |  | **Estimate** | **SE** | **P-value** | **Phet** |
| UKB | PRS-CS | ess_ge10 | 0.16 | 0.03 | 3.23E-06 |  | 0.21 | 0.06 | 4.44E-04 |  | 0.19 | 0.06 | 7.97E-04 |  | 0.25 | 0.08 | 3.06E-03 |  | 0.18 | 0.03 | 3.04E-13 | 0.77 |
| MVP EUR | PRS-CS | ess_ge10 | 0.11 | 0.05 | 1.47E-02 |  | 0.33 | 0.15 | 2.78E-02 |  | -0.06 | 0.06 | 3.27E-01 |  | 0.03 | 0.22 | 8.99E-01 |  | 0.06 | 0.03 | 8.11E-02 | 0.03 |
| MVP EUR | PRS-CSx | ess_ge10 | 0.16 | 0.05 | 2.23E-03 |  | 0.48 | 0.14 | 7.15E-04 |  | 0.05 | 0.06 | 3.71E-01 |  | 0.29 | 0.19 | 1.32E-01 |  | 0.14 | 0.04 | 1.14E-04 | 0.03 |
| UKB+MVP EUR (meta) | PRS-CS | ess_ge10 | 0.13 | 0.03 | 7.60E-05 |  | 0.07 | 0.06 | 2.79E-01 |  | 0.01 | 0.06 | 8.51E-01 |  | 0.08 | 0.08 | 3.06E-01 |  | 0.09 | 0.02 | 1.44E-04 | 0.30 |
| UKB + MVP EUR (sum) | PRS-CS | ess_ge10 | 0.20 | 0.04 | 1.44E-06 |  | 0.35 | 0.09 | 6.96E-05 |  | 0.10 | 0.06 | 8.30E-02 |  | 0.33 | 0.12 | 6.83E-03 |  | 0.20 | 0.03 | 6.58E-11 | 0.06 |
| UKB + MVP EUR (sum) | PRS-CS PRS-CSx | ess_ge10 | 0.22 | 0.04 | 5.89E-07 |  | 0.38 | 0.09 | 9.27E-06 |  | 0.17 | 0.06 | 2.33E-03 |  | 0.37 | 0.12 | 1.59E-03 |  | 0.24 | 0.03 | 1.84E-14 | 0.13 |
| UKB | PRS-CS | ess_ge11 | 0.23 | 0.04 | 2.12E-08 |  | 0.21 | 0.07 | 2.32E-03 |  | 0.16 | 0.06 | 5.56E-03 |  | 0.22 | 0.09 | 1.61E-02 |  | 0.21 | 0.03 | 1.83E-13 | 0.83 |
| MVP EUR | PRS-CS | ess_ge11 | 0.13 | 0.05 | 8.63E-03 |  | 0.18 | 0.17 | 2.92E-01 |  | -0.06 | 0.06 | 3.30E-01 |  | -0.03 | 0.24 | 9.07E-01 |  | 0.06 | 0.04 | 1.13E-01 | 0.09 |
| MVP EUR | PRS-CSx | ess_ge11 | 0.17 | 0.05 | 1.03E-03 |  | 0.34 | 0.16 | 3.58E-02 |  | 0.06 | 0.06 | 2.98E-01 |  | 0.21 | 0.21 | 3.33E-01 |  | 0.14 | 0.04 | 2.32E-04 | 0.29 |
| UKB+MVP EUR (meta) | PRS-CS | ess_ge11 | 0.16 | 0.03 | 5.77E-06 |  | 0.09 | 0.07 | 1.92E-01 |  | 0.00 | 0.06 | 9.68E-01 |  | 0.08 | 0.09 | 3.77E-01 |  | 0.11 | 0.03 | 2.94E-05 | 0.11 |
| UKB + MVP EUR (sum) | PRS-CS | ess_ge11 | 0.26 | 0.05 | 1.09E-08 |  | 0.31 | 0.10 | 1.67E-03 |  | 0.08 | 0.06 | 1.87E-01 |  | 0.28 | 0.13 | 3.39E-02 |  | 0.21 | 0.03 | 9.24E-11 | 0.05 |
| UKB + MVP EUR (sum) | PRS-CS PRS-CSx | ess_ge11 | 0.29 | 0.05 | 4.46E-09 |  | 0.35 | 0.10 | 4.11E-04 |  | 0.16 | 0.06 | 6.48E-03 |  | 0.32 | 0.13 | 1.36E-02 |  | 0.26 | 0.03 | 3.83E-14 | 0.24 |
| UKB | PRS-CS | ess_ge12 | 0.26 | 0.04 | 3.40E-10 |  | 0.20 | 0.08 | 7.02E-03 |  | 0.17 | 0.06 | 6.39E-03 |  | 0.27 | 0.10 | 8.27E-03 |  | 0.23 | 0.03 | 6.62E-15 | 0.65 |
| MVP EUR | PRS-CS | ess_ge12 | 0.11 | 0.05 | 3.05E-02 |  | 0.24 | 0.19 | 2.05E-01 |  | -0.08 | 0.07 | 2.06E-01 |  | -0.13 | 0.27 | 6.37E-01 |  | 0.04 | 0.04 | 2.79E-01 | 0.07 |
| MVP EUR | PRS-CSx | ess_ge12 | 0.17 | 0.06 | 3.55E-03 |  | 0.34 | 0.18 | 5.77E-02 |  | 0.04 | 0.07 | 5.33E-01 |  | 0.16 | 0.24 | 4.86E-01 |  | 0.13 | 0.04 | 2.39E-03 | 0.28 |
| UKB+MVP EUR (meta) | PRS-CS | ess_ge12 | 0.16 | 0.04 | 1.94E-05 |  | 0.07 | 0.08 | 3.23E-01 |  | 0.03 | 0.06 | 6.65E-01 |  | 0.15 | 0.10 | 1.24E-01 |  | 0.12 | 0.03 | 1.90E-05 | 0.29 |
| UKB + MVP EUR (sum) | PRS-CS | ess_ge12 | 0.29 | 0.05 | 4.76E-09 |  | 0.32 | 0.11 | 3.66E-03 |  | 0.07 | 0.07 | 2.77E-01 |  | 0.32 | 0.15 | 2.69E-02 |  | 0.23 | 0.04 | 1.29E-10 | 0.04 |
| UKB + MVP EUR (sum) | PRS-CS PRS-CSx | ess_ge12 | 0.32 | 0.05 | 6.12E-10 |  | 0.34 | 0.11 | 1.65E-03 |  | 0.16 | 0.07 | 1.62E-02 |  | 0.37 | 0.14 | 1.01E-02 |  | 0.27 | 0.04 | 5.06E-14 | 0.17 |
| UKB | PRS-CS | slpyday_gt1 | 0.06 | 0.03 | 2.89E-02 |  | 0.01 | 0.05 | 8.97E-01 |  | -0.03 | 0.09 | 7.63E-01 |  | 0.16 | 0.07 | 1.46E-02 |  | 0.06 | 0.02 | 1.38E-02 | 0.19 |
| MVP EUR | PRS-CS | slpyday_gt1 | 0.04 | 0.04 | 3.17E-01 |  | 0.28 | 0.12 | 1.43E-02 |  | -0.02 | 0.09 | 8.48E-01 |  | 0.19 | 0.18 | 2.80E-01 |  | 0.05 | 0.03 | 9.67E-02 | 0.15 |
| MVP EUR | PRS-CSx | slpyday_gt1 | 0.07 | 0.04 | 7.87E-02 |  | 0.23 | 0.11 | 3.36E-02 |  | 0.18 | 0.09 | 3.89E-02 |  | 0.08 | 0.16 | 6.31E-01 |  | 0.11 | 0.03 | 2.17E-03 | 0.44 |
| UKB+MVP EUR (meta) | PRS-CS | slpyday_gt1 | 0.06 | 0.03 | 2.79E-02 |  | -0.04 | 0.05 | 4.28E-01 |  | -0.07 | 0.09 | 4.46E-01 |  | 0.05 | 0.07 | 4.71E-01 |  | 0.03 | 0.02 | 1.60E-01 | 0.20 |
| UKB + MVP EUR (sum) | PRS-CS | slpyday_gt1 | 0.08 | 0.03 | 2.70E-02 |  | 0.07 | 0.07 | 3.19E-01 |  | -0.03 | 0.09 | 7.28E-01 |  | 0.25 | 0.10 | 1.03E-02 |  | 0.08 | 0.03 | 5.01E-03 | 0.19 |
| UKB + MVP EUR (sum) | PRS-CS PRS-CSx | slpyday_gt1 | 0.09 | 0.04 | 1.17E-02 |  | 0.06 | 0.07 | 3.59E-01 |  | 0.10 | 0.09 | 2.29E-01 |  | 0.22 | 0.10 | 2.00E-02 |  | 0.10 | 0.03 | 5.15E-04 | 0.57 |
| UKB | PRS-CS | slpyday_gt2 | 0.06 | 0.03 | 4.57E-02 |  | -0.04 | 0.05 | 3.58E-01 |  | 0.04 | 0.05 | 4.36E-01 |  | 0.10 | 0.07 | 1.32E-01 |  | 0.04 | 0.02 | 6.38E-02 | 0.23 |
| MVP EUR | PRS-CS | slpyday_gt2 | 0.08 | 0.03 | 2.09E-02 |  | 0.24 | 0.12 | 5.21E-02 |  | 0.01 | 0.05 | 9.24E-01 |  | 0.04 | 0.18 | 8.23E-01 |  | 0.07 | 0.03 | 1.67E-02 | 0.33 |
| MVP EUR | PRS-CSx | slpyday_gt2 | 0.13 | 0.04 | 1.29E-03 |  | 0.18 | 0.11 | 1.21E-01 |  | -0.02 | 0.05 | 6.49E-01 |  | -0.07 | 0.16 | 6.53E-01 |  | 0.08 | 0.03 | 1.13E-02 | 0.08 |
| UKB+MVP EUR (meta) | PRS-CS | slpyday_gt2 | 0.06 | 0.03 | 3.99E-02 |  | -0.05 | 0.05 | 3.19E-01 |  | -0.04 | 0.05 | 4.61E-01 |  | 0.06 | 0.07 | 3.82E-01 |  | 0.02 | 0.02 | 2.59E-01 | 0.15 |
| UKB + MVP EUR (sum) | PRS-CS | slpyday_gt2 | 0.09 | 0.03 | 6.40E-03 |  | -0.01 | 0.07 | 8.90E-01 |  | 0.03 | 0.05 | 5.35E-01 |  | 0.14 | 0.10 | 1.52E-01 |  | 0.07 | 0.03 | 7.28E-03 | 0.43 |
| UKB + MVP EUR (sum) | PRS-CS PRS-CSx | slpyday_gt2 | 0.11 | 0.04 | 2.12E-03 |  | -0.02 | 0.07 | 8.10E-01 |  | 0.01 | 0.05 | 8.03E-01 |  | 0.11 | 0.10 | 2.48E-01 |  | 0.07 | 0.03 | 9.33E-03 | 0.24 |
| UKB | PRS-CS | slpyday_gt4 | 0.11 | 0.05 | 1.37E-02 |  | -0.04 | 0.08 | 6.54E-01 |  | 0.24 | 0.18 | 1.89E-01 |  | 0.24 | 0.13 | 5.61E-02 |  | 0.10 | 0.04 | 8.16E-03 | 0.20 |
| MVP EUR | PRS-CS | slpyday_gt4 | 0.08 | 0.06 | 1.55E-01 |  | 0.57 | 0.21 | 6.99E-03 |  | -0.06 | 0.18 | 7.46E-01 |  | -0.12 | 0.34 | 7.29E-01 |  | 0.10 | 0.05 | 6.71E-02 | 0.10 |
| MVP EUR | PRS-CSx | slpyday_gt4 | 0.10 | 0.07 | 1.39E-01 |  | 0.58 | 0.20 | 3.11E-03 |  | -0.18 | 0.18 | 3.34E-01 |  | -0.23 | 0.29 | 4.36E-01 |  | 0.10 | 0.06 | 8.65E-02 | 0.02 |
| UKB+MVP EUR (meta) | PRS-CS | slpyday_gt4 | 0.09 | 0.04 | 5.20E-02 |  | -0.12 | 0.09 | 1.54E-01 |  | -0.12 | 0.18 | 5.03E-01 |  | 0.02 | 0.13 | 8.95E-01 |  | 0.03 | 0.04 | 3.54E-01 | 0.14 |
| UKB + MVP EUR (sum) | PRS-CS | slpyday_gt4 | 0.14 | 0.05 | 7.28E-03 |  | 0.07 | 0.12 | 5.77E-01 |  | 0.13 | 0.18 | 4.80E-01 |  | 0.29 | 0.18 | 1.08E-01 |  | 0.14 | 0.04 | 2.00E-03 | 0.78 |
| UKB + MVP EUR (sum) | PRS-CS PRS-CSx | slpyday_gt4 | 0.15 | 0.06 | 8.13E-03 |  | 0.08 | 0.12 | 4.76E-01 |  | 0.05 | 0.18 | 7.84E-01 |  | 0.25 | 0.18 | 1.65E-01 |  | 0.14 | 0.05 | 3.18E-03 | 0.84 |
| UKB | PRS-CS | ess | 0.47 | 0.06 | 1.20E-12 |  | 0.43 | 0.09 | 2.38E-06 |  | 0.50 | 0.11 | 1.06E-05 |  | 0.48 | 0.15 | 1.05E-03 |  | 0.47 | 0.05 | 1.24E-24 | 0.96 |
| MVP EUR | PRS-CS | ess | 0.26 | 0.08 | 1.75E-03 |  | 0.82 | 0.23 | 3.38E-04 |  | -0.10 | 0.12 | 3.69E-01 |  | -0.19 | 0.39 | 6.35E-01 |  | 0.18 | 0.06 | 4.68E-03 | 0.001 |
| MVP EUR | PRS-CSx | ess | 0.39 | 0.09 | 1.06E-05 |  | 0.94 | 0.22 | 1.31E-05 |  | 0.08 | 0.12 | 4.73E-01 |  | 0.22 | 0.35 | 5.27E-01 |  | 0.34 | 0.07 | 2.65E-07 | 0.004 |
| UKB+MVP EUR (meta) | PRS-CS | ess | 0.31 | 0.06 | 1.80E-07 |  | 0.15 | 0.10 | 1.14E-01 |  | 0.10 | 0.11 | 3.75E-01 |  | 0.27 | 0.15 | 8.11E-02 |  | 0.24 | 0.04 | 3.42E-08 | 0.29 |
| UKB + MVP EUR (sum) | PRS-CS | ess | 0.55 | 0.07 | 8.56E-13 |  | 0.74 | 0.13 | 2.44E-08 |  | 0.29 | 0.12 | 1.18E-02 |  | 0.59 | 0.21 | 5.24E-03 |  | 0.52 | 0.05 | 9.37E-22 | 0.07 |
| UKB + MVP EUR (sum) | PRS-CS PRS-CSx | ess | 0.61 | 0.08 | 4.63E-15 |  | 0.77 | 0.13 | 3.84E-09 |  | 0.42 | 0.11 | 2.38E-04 |  | 0.65 | 0.21 | 1.83E-03 |  | 0.60 | 0.06 | 1.27E-27 | 0.23 |

Beta and SE (standard error) for log odds ratio; ESS=Epworth Sleepiness Scale; Slpyday=General question for excessive daytime sleepiness

**Supplemental Table 9.** Study-Specific and Meta-Analysis Results for the Association between Sleep Phenotype Definitions for Short Sleep, Long Sleep, Insomnia and

Daytime Sleepiness and DSST

|  |  | **ARIC** | | | | **HCHS/SOL** | | | | **MESA** | | | | **BHS** | | | |
| --- | --- | --- | --- | --- | --- | --- | --- | --- | --- | --- | --- | --- | --- | --- | --- | --- | --- |
| **Trait** | **Exposure** | **N** | **Beta** | **SE** | **Pvalue** | **N** | **Beta** | **SE** | **Pvalue** | **N** | **Beta** | **SE** | **Pvalue** | **N** | **Beta** | **SE** | **Pvalue** |
| Short Sleep | ≤5 | 1640 | -0.05 | 0.15 | 0.73 | 8972 | 0.11 | 0.12 | 0.33 | 1924 | -0.09 | 0.14 | 0.53 | 991 | -0.28 | 0.10 | 5.14E-03 |
| Short Sleep | <6 | 1640 | -0.16 | 0.09 | 0.08 | 8972 | 0.11 | 0.07 | 0.10 | 1924 | -0.12 | 0.09 | 0.18 | 991 | -0.21 | 0.08 | 0.01 |
| Short Sleep | ≤6 | 1640 | -0.18 | 0.08 | 0.02 | 8972 | 0.05 | 0.05 | 0.36 | 1924 | -0.13 | 0.07 | 0.07 | 991 | -0.12 | 0.07 | 0.07 |
| Short Sleep | <7 | 1640 | -0.06 | 0.05 | 0.22 | 8972 | 0.13 | 0.04 | 1.83E-04 | 1924 | -0.12 | 0.05 | 0.02 | 991 | -0.06 | 0.06 | 0.32 |
| Long Sleep | >8 | 1640 | -0.12 | 0.05 | 0.02 | 8972 | -0.16 | 0.03 | 1.20E-07 | 1924 | -0.10 | 0.04 | 0.01 | 991 | -0.04 | 0.08 | 0.67 |
| Long Sleep | ≥9 | 1640 | -0.22 | 0.09 | 0.01 | 8972 | -0.24 | 0.03 | 6.67E-12 | 1924 | -0.21 | 0.05 | 5.27E-06 | 991 | -0.26 | 0.12 | 0.02 |
| Long Sleep | >9 | 1640 | -0.31 | 0.12 | 0.009 | 8972 | -0.25 | 0.04 | 9.00E-12 | 1924 | -0.24 | 0.05 | 3.44E-06 | 991 | -0.20 | 0.13 | 0.12 |
| Long Sleep | ≥10 | 1640 | -0.56 | 0.23 | 0.01 | 8972 | -0.33 | 0.05 | 9.26E-12 | 1924 | -0.23 | 0.07 | 0.002 | 991 | -0.31 | 0.17 | 0.07 |
| Insomnia WHIIRS | ≥9 | NA | NA | NA | NA | 9115 | -0.06 | 0.03 | 0.03 | 1927 | -0.08 | 0.04 | 0.07 | 1067 | -0.22 | 0.06 | 7.46E-05 |
| Insomnia WHIIRS | ≥10 | NA | NA | NA | NA | 9115 | -0.08 | 0.03 | 0.01 | 1927 | -0.09 | 0.04 | 0.05 | 1067 | -0.19 | 0.06 | 9.03E-04 |
| Insomnia WHIIRS 3Q | ≥5 | 1668 | -0.01 | 0.04 | 0.85 | 9126 | -0.09 | 0.03 | 0.003 | 1927 | -0.04 | 0.04 | 0.35 | 1071 | -0.16 | 0.06 | 3.96E-03 |
| Insomnia WHIIRS 3Q | ≥6 | 1668 | 0.04 | 0.04 | 0.39 | 9126 | -0.08 | 0.03 | 0.01 | 1927 | -0.08 | 0.05 | 0.08 | 1071 | -0.16 | 0.06 | 5.04E-03 |
| Insomnia Dr Dx | Yes | NA | NA | NA | NA | 9331 | -0.24 | 0.05 | 7.99E-07 | 1934 | -0.06 | 0.08 | 0.48 | 1024 | 0.44 | 0.10 | 1.11E-05 |
| Daytime Sleepiness ESS | ≥10 | 1642 | -0.06 | 0.04 | 0.15 | 9218 | -0.02 | 0.03 | 0.64 | 1932 | -0.16 | 0.05 | 0.002 | 1072 | -0.12 | 0.07 | 0.08 |
| Daytime Sleepiness ESS | ≥11 | 1642 | -0.08 | 0.05 | 0.11 | 9218 | -0.02 | 0.04 | 0.61 | 1932 | -0.19 | 0.06 | 8.2E-04 | 1072 | -0.14 | 0.08 | 0.08 |
| Daytime Sleepiness ESS | ≥12 | 1642 | -0.06 | 0.05 | 0.22 | 9218 | -0.02 | 0.04 | 0.64 | 1932 | -0.20 | 0.06 | 0.002 | 1072 | -0.10 | 0.09 | 0.25 |
| Daytime Sleepiness GenQ | >1 | 1678 | 0.10 | 0.07 | 0.12 | 9247 | -0.03 | 0.03 | 0.23 | 1939 | -0.04 | 0.04 | 0.36 | 1075 | -0.12 | 0.06 | 0.04 |
| Daytime Sleepiness GenQ | >2 | 1678 | -0.06 | 0.04 | 0.15 | 9247 | -0.05 | 0.03 | 0.09 | 1939 | -0.09 | 0.04 | 0.02 | 1075 | -0.13 | 0.06 | 0.02 |
| Daytime Sleepiness GenQ | >4 | 1678 | 0.08 | 0.15 | 0.61 | 9247 | -0.14 | 0.05 | 0.01 | 1939 | -0.20 | 0.07 | 0.005 | 1075 | -0.21 | 0.11 | 0.06 |
|  |  | **Meta-Analysis (Fixed Effects)** | | | | **Meta-Analysis (Random Effects)** | | |  |  |  |  |  |  |  |  |  |
|  |  | **Beta** | **SE** | **Pvalue** | **P Heterogeneity** | **Beta** | **SE** | **Pvalue** |  |  |  |  |  |  |  |  |  |
| Short Sleep | ≤5 | -0.10 | 0.06 | 0.10 | 0.08 | -0.08 | 0.09 | 0.36 |  |  |  |  |  |  |  |  |  |
| Short Sleep | <6 | -0.07 | 0.04 | 0.10 | 0.01 | -0.09 | 0.08 | 0.25 |  |  |  |  |  |  |  |  |  |
| Short Sleep | ≤6 | -0.07 | 0.03 | 0.03 | 0.04 | -0.09 | 0.05 | 0.10 |  |  |  |  |  |  |  |  |  |
| Short Sleep | <7 | 0.01 | 0.02 | 0.67 | 0.00 | -0.02 | 0.06 | 0.70 |  |  |  |  |  |  |  |  |  |
| Long Sleep | >8 | -0.13 | 0.02 | 8.03E-10 | 0.43 | -0.13 | 0.02 | 6.66E-09 |  |  |  |  |  |  |  |  |  |
| Long Sleep | ≥9 | -0.23 | 0.03 | 1.46E-19 | 0.96 | -0.23 | 0.03 | 1.46E-19 |  |  |  |  |  |  |  |  |  |
| Long Sleep | >9 | -0.25 | 0.03 | 9.52E-19 | 0.94 | -0.25 | 0.03 | 9.52E-19 |  |  |  |  |  |  |  |  |  |
| Long Sleep | ≥10 | -0.30 | 0.04 | 1.00E-15 | 0.45 | -0.30 | 0.04 | 2.36E-15 |  |  |  |  |  |  |  |  |  |
| Insomnia WHIIRS | ≥9 | -0.09 | 0.02 | 3.04E-05 | 0.04 | -0.11 | 0.05 | 0.02 |  |  |  |  |  |  |  |  |  |
| Insomnia WHIIRS | ≥10 | -0.10 | 0.02 | 2.01E-05 | 0.21 | -0.10 | 0.03 | 2.30E-04 |  |  |  |  |  |  |  |  |  |
| Insomnia WHIIRS 3Q | ≥5 | -0.07 | 0.02 | 3.75E-04 | 0.13 | -0.07 | 0.03 | 0.01 |  |  |  |  |  |  |  |  |  |
| Insomnia WHIIRS 3Q | ≥6 | -0.06 | 0.02 | 2.92E-03 | 0.03 | -0.07 | 0.04 | 0.08 |  |  |  |  |  |  |  |  |  |
| Insomnia | ≥9, ≥5 | -0.07 | 0.02 | 1.38E-04 | 0.02 | -0.09 | 0.04 | 0.03 |  |  |  |  |  |  |  |  |  |
| Insomnia | ≥10, ≥6 | -0.07 | 0.02 | 9.11E-04 | 0.01 | -0.08 | 0.04 | 0.08 |  |  |  |  |  |  |  |  |  |
| Insomnia Dr Dx | Yes | -0.19 | 0.04 | 2.49E-06 | 0.05 | -0.16 | 0.09 | 0.08 |  |  |  |  |  |  |  |  |  |
| Daytime Sleepiness ESS | ≥10 | -0.07 | 0.02 | 2.68E-03 | 0.11 | -0.08 | 0.03 | 0.02 |  |  |  |  |  |  |  |  |  |
| Daytime Sleepiness ESS | ≥11 | -0.08 | 0.03 | 1.25E-03 | 0.08 | -0.10 | 0.04 | 0.02 |  |  |  |  |  |  |  |  |  |
| Daytime Sleepiness ESS | ≥12 | -0.07 | 0.03 | 7.91E-03 | 0.13 | -0.08 | 0.04 | 0.03 |  |  |  |  |  |  |  |  |  |
| Daytime Sleepiness GenQ | >1 | -0.03 | 0.02 | 0.11 | 0.09 | -0.03 | 0.03 | 0.37 |  |  |  |  |  |  |  |  |  |
| Daytime Sleepiness GenQ | >2 | -0.07 | 0.02 | 3.19E-04 | 0.55 | -0.07 | 0.02 | 3.19E-04 |  |  |  |  |  |  |  |  |  |
| Daytime Sleepiness GenQ | >4 | -0.15 | 0.04 | 7.21E-05 | 0.39 | -0.15 | 0.04 | 7.21E-05 |  |  |  |  |  |  |  |  |  |

3Q = 3 questions, Dr Dx = Self-reported doctor diagnosis, GenQ = General question

**Supplemental Table 10.** Study-Specific and Meta-Analysis Results for the Association between Sleep Phenotype Definitions for Short Sleep, Long Sleep, Insomnia, and Daytime Sleepiness and Hypertension

|  |  | **ARIC** | | | | | | | **HCHS/SOL** | | | | | | | **MESA** | | | | | | | **BHS** | | | | | |  |
| --- | --- | --- | --- | --- | --- | --- | --- | --- | --- | --- | --- | --- | --- | --- | --- | --- | --- | --- | --- | --- | --- | --- | --- | --- | --- | --- | --- | --- | --- |
| **Trait** | **Exposure** | **N** | **N_Case** | **Beta** | **SE** | **OR** | **95% CI** | **Pvalue** | **N** | **N_Case** | **Beta** | **SE** | **OR** | **95% CI** | **Pvalue** | **N** | **N_Case** | **Beta** | **SE** | **OR** | **95% CI** | **Pvalue** | **N** | **N_Case** | **Beta** | **SE** | **OR** | **95% CI** | **Pvalue** |
| Short Sleep | ≤5 | 1781 | 712 | 0.13 | 0.36 | 1.13 | 0.55, 2.31 | 0.73 | 15489 | 4643 | 0.04 | 0.18 | 1.04 | 0.73, 1.48 | 0.83 | 2122 | 1276 | 0.36 | 0.35 | 1.43 | 0.71, 2.86 | 0.31 | 991 | 439 | 0.13 | 0.23 | 1.13 | 0.72-1.8 | 0.59 |
| Short Sleep | <6 | 1781 | 712 | -0.13 | 0.23 | 0.88 | 0.56, 1.37 | 0.58 | 15489 | 4643 | -0.09 | 0.11 | 0.91 | 0.73, 1.14 | 0.42 | 2122 | 1276 | 0.03 | 0.22 | 1.03 | 0.67, 1.57 | 0.89 | 991 | 439 | 0.10 | 0.19 | 1.11 | 0.76-1.61 | 0.60 |
| Short Sleep | ≤6 | 1781 | 712 | 0.05 | 0.19 | 1.05 | 0.73, 1.51 | 0.79 | 15489 | 4643 | -0.11 | 0.09 | 0.90 | 0.75, 1.08 | 0.25 | 2122 | 1276 | 0.02 | 0.17 | 1.02 | 0.73, 1.42 | 0.92 | 991 | 439 | 0.18 | 0.15 | 1.19 | 0.88-1.61 | 0.25 |
| Short Sleep | <7 | 1781 | 712 | -0.10 | 0.12 | 0.91 | 0.72, 1.14 | 0.42 | 15489 | 4643 | -0.06 | 0.07 | 0.94 | 0.82, 1.08 | 0.40 | 2122 | 1276 | -0.06 | 0.12 | 0.95 | 0.75, 1.2 | 0.65 | 991 | 439 | 0.10 | 0.14 | 1.11 | 0.84-1.46 | 0.46 |
| Long Sleep | >8 | 1781 | 712 | 0.41 | 0.12 | 1.51 | 1.19, 1.92 | 6.98E-04 | 15489 | 4643 | 0.13 | 0.06 | 1.14 | 1.01, 1.28 | 0.03 | 2122 | 1276 | 0.07 | 0.10 | 1.07 | 0.88, 1.3 | 0.49 | 991 | 439 | -0.20 | 0.20 | 0.82 | 0.55-1.22 | 0.32 |
| Long Sleep | ≥9 | 1781 | 712 | 0.90 | 0.21 | 2.46 | 1.62, 3.73 | 2.32E-05 | 15489 | 4643 | 0.22 | 0.08 | 1.25 | 1.07, 1.45 | 0.004 | 2122 | 1276 | 0.07 | 0.12 | 1.07 | 0.85, 1.35 | 0.54 | 991 | 439 | -0.27 | 0.28 | 0.76 | 0.44-1.32 | 0.34 |
| Long Sleep | >9 | 1781 | 712 | 0.99 | 0.30 | 2.70 | 1.51, 4.85 | 8.66E-04 | 15489 | 4643 | 0.18 | 0.08 | 1.20 | 1.02, 1.42 | 0.03 | 2122 | 1276 | 0.02 | 0.13 | 1.02 | 0.79, 1.32 | 0.85 | 991 | 439 | -0.20 | 0.32 | 0.82 | 0.44-1.53 | 0.53 |
| Long Sleep | ≥10 | 1781 | 712 | 1.24 | 0.61 | 3.44 | 1.03, 11.46 | 0.04 | 15489 | 4643 | 0.26 | 0.13 | 1.30 | 1.02, 1.67 | 0.04 | 2122 | 1276 | -0.05 | 0.18 | 0.95 | 0.67, 1.35 | 0.78 | 991 | 439 | -0.57 | 0.41 | 0.56 | 0.25-1.27 | 0.17 |
| Insomnia WHIIRS | ≥9 | NA | NA | NA | NA | NA | NA | NA | 15812 | 4732 | 0.19 | 0.06 | 1.21 | 1.06, 1.37 | 0.004 | 2128 | 1285 | 0.01 | 0.10 | 1.01 | 0.83, 1.24 | 0.89 | 1067 | 480 | 0.36 | 0.13 | 1.44 | 1.11-1.87 | 0.01 |
| Insomnia WHIIRS | ≥10 | NA | NA | NA | NA | NA | NA | NA | 15812 | 4732 | 0.24 | 0.06 | 1.27 | 1.12, 1.44 | 1.79E-04 | 2128 | 1285 | -0.04 | 0.11 | 0.96 | 0.78, 1.18 | 0.67 | 1067 | 480 | 0.27 | 0.13 | 1.31 | 1-1.7 | 0.05 |
| Insomnia WHIIRS 3Q | ≥5 | 1814 | 731 | -0.03 | 0.11 | 0.97 | 0.79, 1.20 | 0.78 | 15833 | 4738 | 0.17 | 0.07 | 1.19 | 1.04, 1.36 | 0.01 | 2128 | 1285 | -0.11 | 0.11 | 0.90 | 0.72, 1.11 | 0.31 | 1071 | 481 | 0.33 | 0.13 | 1.39 | 1.07-1.82 | 0.01 |
| Insomnia WHIIRS 3Q | ≥6 | 1814 | 731 | -0.01 | 0.10 | 0.99 | 0.81, 1.21 | 0.93 | 15833 | 4738 | 0.18 | 0.07 | 1.19 | 1.04, 1.37 | 0.01 | 2128 | 1285 | -0.02 | 0.11 | 0.98 | 0.78, 1.23 | 0.87 | 1071 | 481 | 0.32 | 0.14 | 1.37 | 1.05-1.8 | 0.02 |
| Insomnia Dr Dx | Yes | NA | NA | NA | NA | NA | NA | NA | 16242 | 4862 | 0.39 | 0.10 | 1.48 | 1.22, 1.8 | 8.53E-05 | 2134 | 1287 | 0.24 | 0.20 | 1.27 | 0.87, 1.88 | 0.22 | 1024 | 455 | -0.34 | 0.24 | 0.71 | 0.44-1.14 | 0.16 |
| Daytime Sleepiness ESS | ≥10 | 1788 | 723 | -0.06 | 0.11 | 0.95 | 0.77, 1.17 | 0.61 | 15989 | 4780 | -0.07 | 0.08 | 0.93 | 0.8, 1.08 | 0.36 | 2133 | 1290 | 0.20 | 0.13 | 1.22 | 0.95, 1.56 | 0.12 | 1072 | 481 | 0.08 | 0.17 | 1.08 | 0.78-1.5 | 0.63 |
| Daytime Sleepiness ESS | ≥11 | 1788 | 723 | -0.004 | 0.11 | 1.00 | 0.80, 1.25 | 0.97 | 15989 | 4780 | -0.08 | 0.09 | 0.92 | 0.78, 1.09 | 0.34 | 2133 | 1290 | 0.29 | 0.14 | 1.33 | 1, 1.77 | 0.05 | 1072 | 481 | 0.16 | 0.18 | 1.18 | 0.82-1.69 | 0.37 |
| Daytime Sleepiness ESS | ≥12 | 1788 | 723 | 0.06 | 0.12 | 1.07 | 0.84, 1.36 | 0.60 | 15989 | 4780 | -0.07 | 0.09 | 0.93 | 0.78, 1.11 | 0.45 | 2133 | 1290 | 0.29 | 0.16 | 1.33 | 0.97, 1.82 | 0.08 | 1072 | 481 | 0.14 | 0.20 | 1.15 | 0.77-1.72 | 0.48 |
| Daytime Sleepiness GenQ | >1 | 1825 | 733 | 0.14 | 0.16 | 1.15 | 0.84, 1.58 | 0.37 | 16032 | 4803 | 0.07 | 0.06 | 1.08 | 0.96, 1.21 | 0.21 | 2140 | 1292 | -0.05 | 0.10 | 0.95 | 0.78, 1.15 | 0.59 | 1075 | 482 | 0.19 | 0.14 | 1.21 | 0.93-1.59 | 0.16 |
| Daytime Sleepiness GenQ | >2 | 1825 | 733 | 0.03 | 0.10 | 1.03 | 0.85, 1.26 | 0.75 | 16032 | 4803 | 0.07 | 0.06 | 1.07 | 0.94, 1.21 | 0.30 | 2140 | 1292 | 0.09 | 0.10 | 1.09 | 0.89, 1.33 | 0.39 | 1075 | 482 | 0.14 | 0.14 | 1.15 | 0.87-1.51 | 0.32 |
| Daytime Sleepiness GenQ | >4 | 1825 | 733 | -0.01 | 0.37 | 0.99 | 0.48, 2.05 | 0.98 | 16032 | 4803 | 0.09 | 0.09 | 1.10 | 0.93, 1.3 | 0.29 | 2140 | 1292 | 0.29 | 0.18 | 1.33 | 0.93, 1.91 | 0.12 | 1075 | 482 | 0.31 | 0.26 | 1.37 | 0.82-2.3 | 0.23 |
|  |  |  |  | **Meta-Analysis (Fixed Effects)** | | | | | |  | **Meta-Analysis (Random Effects)** | | | | |  |  |  |  |  |  |  |  |  |  |  |  |  |  |
|  |  |  |  | **Beta** | **SE** | **OR** | **95% CI** | **Pvalue** | **P Het** |  | **Beta** | **SE** | **OR** | **95% CI** | **Pvalue** |  |  |  |  |  |  |  |  |  |  |  |  |  |  |
| Short Sleep | ≤5 |  |  | 0.11 | 0.12 | 1.12 | 0.88-1.43 | 0.36 | 0.89 |  | 0.11 | 0.12 | 1.12 | 0.88, 1.43 | 0.36 |  |  |  |  |  |  |  |  |  |  |  |  |  |  |
| Short Sleep | <6 |  |  | -0.04 | 0.08 | 0.96 | 0.82-1.13 | 0.61 | 0.81 |  | -0.04 | 0.08 | 0.96 | 0.82, 1.13 | 0.61 |  |  |  |  |  |  |  |  |  |  |  |  |  |  |
| Short Sleep | ≤6 |  |  | -0.01 | 0.07 | 0.99 | 0.87-1.13 | 0.85 | 0.44 |  | 0.00 | 0.07 | 1.00 | 0.86, 1.15 | 0.97 |  |  |  |  |  |  |  |  |  |  |  |  |  |  |
| Short Sleep | <7 |  |  | -0.04 | 0.05 | 0.96 | 0.87-1.06 | 0.39 | 0.71 |  | -0.04 | 0.05 | 0.96 | 0.87, 1.06 | 0.39 |  |  |  |  |  |  |  |  |  |  |  |  |  |  |
| Long Sleep | >8 |  |  | 0.14 | 0.05 | 1.15 | 1.05-1.26 | 2.61E-03 | 0.04 |  | 0.13 | 0.10 | 1.14 | 0.94, 1.39 | 0.19 |  |  |  |  |  |  |  |  |  |  |  |  |  |  |
| Long Sleep | ≥9 |  |  | 0.21 | 0.06 | 1.24 | 1.1-1.39 | 3.99E-04 | 0.002 |  | 0.24 | 0.22 | 1.27 | 0.82, 1.96 | 0.28 |  |  |  |  |  |  |  |  |  |  |  |  |  |  |
| Long Sleep | >9 |  |  | 0.17 | 0.07 | 1.18 | 1.04-1.35 | 0.01 | 0.02 |  | 0.23 | 0.22 | 1.26 | 0.82, 1.92 | 0.29 |  |  |  |  |  |  |  |  |  |  |  |  |  |  |
| Long Sleep | ≥10 |  |  | 0.14 | 0.10 | 1.16 | 0.95-1.4 | 0.14 | 0.04 |  | 0.11 | 0.21 | 1.11 | 0.74, 1.68 | 0.61 |  |  |  |  |  |  |  |  |  |  |  |  |  |  |
| Insomnia WHIIRS | ≥9 |  |  | 0.17 | 0.05 | 1.18 | 1.07-1.3 | 1.12E-03 | 0.14 |  | 0.17 | 0.08 | 1.18 | 1.02, 1.37 | 0.03 |  |  |  |  |  |  |  |  |  |  |  |  |  |  |
| Insomnia WHIIRS | ≥10 |  |  | 0.19 | 0.05 | 1.20 | 1.09-1.33 | 2.42E-04 | 0.04 |  | 0.17 | 0.10 | 1.18 | 0.96, 1.45 | 0.11 |  |  |  |  |  |  |  |  |  |  |  |  |  |  |
| Insomnia WHIIRS 3Q | ≥5 |  |  | 0.10 | 0.05 | 1.11 | 1.01-1.22 | 0.03 | 0.02 |  | 0.09 | 0.10 | 1.10 | 0.91, 1.33 | 0.34 |  |  |  |  |  |  |  |  |  |  |  |  |  |  |
| Insomnia WHIIRS 3Q | ≥6 |  |  | 0.11 | 0.05 | 1.12 | 1.01-1.23 | 0.02 | 0.18 |  | 0.10 | 0.06 | 1.11 | 0.98, 1.26 | 0.10 |  |  |  |  |  |  |  |  |  |  |  |  |  |  |
| Insomnia | ≥9, ≥5 |  |  | 0.13 | 0.05 | 1.14 | 1.04-1.24 | 4.79E-03 | 0.09 |  | 0.12 | 0.07 | 1.13 | 0.98, 1.3 | 0.10 |  |  |  |  |  |  |  |  |  |  |  |  |  |  |
| Insomnia | ≥10, ≥6 |  |  | 0.15 | 0.05 | 1.16 | 1.06-1.27 | 1.17E-03 | 0.03 |  | 0.12 | 0.09 | 1.13 | 0.96, 1.34 | 0.15 |  |  |  |  |  |  |  |  |  |  |  |  |  |  |
| Insomnia Dr Dx | Yes |  |  | 0.36 | 0.09 | 1.44 | 1.21-1.71 | 4.28E-05 | 0.50 |  | 0.36 | 0.09 | 1.44 | 1.21, 1.71 | 4.28E-05 |  |  |  |  |  |  |  |  |  |  |  |  |  |  |
| Daytime Sleepiness ESS | ≥10 |  |  | 0.00 | 0.05 | 1.00 | 0.9-1.11 | 0.95 | 0.28 |  | 0.01 | 0.06 | 1.01 | 0.89, 1.14 | 0.90 |  |  |  |  |  |  |  |  |  |  |  |  |  |  |
| Daytime Sleepiness ESS | ≥11 |  |  | 0.02 | 0.06 | 1.03 | 0.91-1.15 | 0.67 | 0.14 |  | 0.06 | 0.09 | 1.06 | 0.89, 1.25 | 0.50 |  |  |  |  |  |  |  |  |  |  |  |  |  |  |
| Daytime Sleepiness ESS | ≥12 |  |  | 0.04 | 0.06 | 1.04 | 0.92-1.18 | 0.51 | 0.26 |  | 0.06 | 0.08 | 1.07 | 0.91, 1.25 | 0.42 |  |  |  |  |  |  |  |  |  |  |  |  |  |  |
| Daytime Sleepiness GenQ | >1 |  |  | 0.06 | 0.05 | 1.07 | 0.98-1.17 | 0.15 | 0.46 |  | 0.06 | 0.05 | 1.07 | 0.98, 1.17 | 0.15 |  |  |  |  |  |  |  |  |  |  |  |  |  |  |
| Daytime Sleepiness GenQ | >2 |  |  | 0.07 | 0.04 | 1.07 | 0.98-1.17 | 0.12 | 0.94 |  | 0.07 | 0.04 | 1.07 | 0.98, 1.17 | 0.12 |  |  |  |  |  |  |  |  |  |  |  |  |  |  |
| Daytime Sleepiness GenQ | >4 |  |  | 0.14 | 0.07 | 1.15 | 0.99-1.32 | 0.06 | 0.67 |  | 0.14 | 0.07 | 1.15 | 0.99, 1.32 | 0.06 |  |  |  |  |  |  |  |  |  |  |  |  |  |  |

Beta and SE (standard error) for log odds ratio, 95% CI (confidence interval) for OR (odds ratio)

3Q = 3 questions, Dr Dx = Self-reported doctor diagnosis, GenQ = General question

**Supplemental Figure 1**. Study-Specific and Meta-Analysis Results for the Association between Sleep PRSs and Continuous Sleep Phenotype Definitions for

Sleep Duration, Insomnia, and Daytime Sleepiness in ARIC (Visit 4), BHS (BiCEPs Visit), HCHS/SOL (Visit 1), and MESA (Visit 5)

**Supplemental Figure 2**. Study-Specific and Meta-Analysis Results for the Association between Sleep PRSs and Sleep Phenotype Definitions for

Short Sleep Long Sleep Insomnia and Daytime Sleepiness, ARIC (Visit 4), BHS (BiCEPs Visit), HCHS/SOL (Visit 1) and MESA (Visit 4)

**Supplemental Figure 3**. Sex-Stratified Study-Specific and Meta-Analysis Results for the Association between Sleep PRSs and Sleep Phenotype Definitions for Short Sleep Long Sleep Insomnia and Daytime Sleepiness, ARIC (Visit 4), BHS (BiCEPs Visit), HCHS/SOL (Visit 1) and MESA (Visit 5). Red=Female and Blue=Male.
